# Combining automated kinetic model selection with robust optimization and application to maximize cell-free production of UDP-GalNAc

**DOI:** 10.1016/j.mec.2026.e00287

**Published:** 2026-09-08

**Authors:** Nicolas Huber, Tuan Son Hoang, Sebastián Espinel Ríos, Thomas F.T. Rexer, Udo Reichl, Steffen Klamt

**Affiliations:** a Max Planck Institute for Dynamics of Complex Technical Systems, Magdeburg, 39106, Germany; b Otto von Guericke University Magdeburg, Magdeburg, 39106, Germany; c School of Chemical and Bioprocess Engineering, University College Dublin, Dublin, D04 V1W8, Ireland; d Eversyn, Magdeburg, 39106, Germany

**Keywords:** Kinetic modeling, Model selection and identification, Structural uncertainty, Robust optimization, Cell-free systems, Multi-enzyme cascades, Nucleotide sugars, UDP-GalNAc

## Abstract

Cell-free multi-enzyme cascades enable precise biosynthesis, but using them for cost-efficient bioproduction remains challenging. This issue can be addressed through model-based process optimization using kinetic models. However, the predictive power of kinetic models is often limited by structural and parametric uncertainties. In this work, we present MoRSel, a novel method that tackles structural uncertainties through automated refinement and selection of kinetic models. MoRSel starts with a kinetic core model, a library of potential model changes, and available experimental data. The algorithm iteratively expands the model to achieve an optimal balance between fit quality and model complexity according to information-theoretic criteria.

We applied MoRSel to a kinetic model of a cell-free enzyme cascade for producing the nucleotide sugar UDP-GalNAc. An initial core model showed significant inconsistencies with measured experimental data. By providing candidates for potential stoichiometric and regulatory changes in the model, MoRSel identified a model that more accurately captured the observed behavior. We then used this model to inform a recently introduced robust (ensemble-based) process optimization strategy that accounts for parametric uncertainty. We addressed two economically relevant problems. First, we determined the optimal initial substrate and enzyme concentrations to maximize the product titer, resulting in a 45 % increase in product titer and yield in a validation experiment. In a second optimization, we minimized the enzyme load and tested the computed optimal enzyme concentrations in another validation experiment. With enzyme costs reduced by 53 %, the relative titer and yield could be improved by 80 % and 75 %, respectively, with small reductions in absolute titer and yield.

Overall, MoRSel advances the development of predictive models for complex biochemical systems, with particular relevance for the robust optimization of cell-free bioproduction processes.

## Highlights

• MoRSel: A novel automated pipeline for the structural refinement and selection of kinetic models.
• Input: kinetic core model, candidate model changes, experimental data; Output: expanded kinetic model best balancing fit quality and complexity.
• Integrating MoRSel with robust optimization effectively addresses structural and parametric uncertainties.
• First end-to-end application to optimize a cell-free enzyme cascade for UDP-GalNAc production.
• Rigorous experimental validation of predicted enzyme ratios demonstrates a 45 % increase in product titer.

## 1 Introduction

The central goal of bioprocess engineering is to design and optimize biochemical production processes. Two primary strategies exist for synthesizing compounds using biocatalysts: *in vivo*, employing whole cells (often genetically modified for product synthesis), or *in vitro*, utilizing isolated cellular components such as enzymes and metabolites in cell-free production systems. While cell-based systems have proven successful across diverse applications and remain the prevailing approach, cell-free systems offer a distinct advantage: by operating independently of cellular constraints (e.g., related to growth, pH, etc.), they enable a wider range of pathway design and operating parameters (Claassens et al., 2019). However, the high costs of cell-free biosynthesis, e.g., due to time-intensive preparatory steps for enzyme production and purification, necessitate robust and first-principles-based optimization methods to bridge the gap to cost-efficient, competitive, and predictable bioproduction (Siedentop et al., 2021).

To address these challenges, the dynamics of cell-free processes, which often involve fewer than a dozen enzymatic steps and thus far fewer components than a cell, can be described, analyzed, and optimized using kinetic models. These models are based on ordinary differential equations composed of kinetic rate laws that describe the kinetics of enzyme-catalyzed reactions in dependency of metabolite concentrations and enzyme abundances (Martin et al., 2023; Shen et al., 2020; Sigg et al., 2021; Silva et al., 2024; Sun et al., 2021; Zhong et al., 2017). For instance, Silva et al. (2024) developed a kinetic model for the cell-free production of fructose from lactose and used it to identify initial enzyme activity ratios that maximize volumetric productivity in batch processes and product titer in continuous setups. Similarly, Sun et al. (2021) and Sigg et al. (2021) used kinetic model simulations to improve the cell-free production of various disaccharides from starch and alpha-glycosyl glycerol from sucrose, respectively. Martin et al. (2023) developed a kinetic model of *E. coli*-based cell-free metabolism and used it to optimize butanol production through model-guided adjustments of cofactor and enzyme levels. Other examples of model-guided exploration and optimization of process dynamics, where optimal enzyme ratios or loadings are identified from kinetic models, include the production of alpha-ketoglutarate from alpha-xylose (Shen et al., 2020) and the cell-free production of cellobiose by Zhong et al. (2017). Notably, many of these model-based optimization studies do not formulate rigorous mathematical optimization problems, defined by explicit objective functions, decision variables, and constraints, that could then be solved using dedicated optimization algorithms. Instead, optimization often relies on heuristic trial-and-error simulations by varying selected parameter values. One central aspect of our recent work (Huber et al., 2024) was therefore to highlight and demonstrate the use of a rigorous optimization framework for identifying optimal process variables (e.g., enzyme ratios) by maximizing or minimizing a given economic objective function.

Another issue addressed in our previous work (Huber et al., 2024) was the adequate consideration of parametric uncertainty when using a kinetic model in a rigorous optimization approach. Rate laws of kinetic models involve (kinetic) parameters, some of which might be available from literature or databases. In many cases, however, parameter values must be estimated from experimental data. Depending on the problem definition, the available experimental data, and the specific parameter estimation methodology, kinetic parameters are often not uniquely identifiable and repeating the parameter estimation with different seeds can lead to different parameter sets that fit the data equally well. Different methods exist to quantify this parameter uncertainty (Heinrich et al., 2025; Mitra and Hlavacek, 2019; Vanlier et al., 2013; Wieland et al., 2021), including profile likelihoods (Kreutz et al., 2013; Raue et al., 2009), sensitivity analysis (Marino et al., 2008), and Bayesian approaches (Schmidt et al., 2020; Vernon et al., 2018). Many of these approaches employ ensemble strategies where, instead of trying to identify one unique set of model parameters, the focus is placed on exploring and capturing a variety of possible model behaviors. In particular, Bayesian approaches often rely on ensemble strategies (Groves et al., 2024; Linden et al., 2022; van Mourik et al., 2014), but they have also been applied in other contexts (Hu et al., 2025). In our previous work (Huber et al., 2024), we proposed a robust ensemble-based optimization strategy to tackle parametric uncertainty. By repeating a least-squares parameter estimation, we generated an ensemble of different parameter sets that all fit the data equally well within a defined tolerance, thus capturing the parameter uncertainty. Then, as a new approach for (robust) optimization on model ensembles, we solved the optimization problem for each parameter set in the ensemble, followed by cross-validation and scoring of all optimization results across the entire parameter ensemble.

However, in addition to parametric non-identifiabilities, there is another source of uncertainty when building enzyme-kinetic models, concerning the model structure itself. Structural (*epistemic*) uncertainties include, for example, the set of reactions to consider, the kinetic rate laws to use, and potentially relevant regulations of the enzymes. For example, apart from unknown enzymatic mechanisms, enzymes from different organisms, along with their associated substrates and cofactors, are often mixed at non-physiologically high concentrations in cell-free systems, potentially leading to unknown side reactions or alterations in enzyme activity. In such cases, especially when a constructed and fitted kinetic model cannot satisfactorily reflect experimental data, methods to identify missing (or irrelevant) components or interactions would be highly valuable. A variety of methods address this problem in different ways, which can broadly be categorized into three major classes: (1) model building via machine-learning methods involving no or only limited prior knowledge, (2) ensemble-based model selection, and (3) iterative improvement of an initial model candidate (and combinations of these three basic approaches).

Methods with a focus on building models purely from available data include DeepMoD, which uses deep learning for model discovery in noisy data (Both et al., 2021), and SINDy, a sparse regression-based method to find a linear combination of basis functions that best capture the behavior of the dynamical system (Brunton et al., 2016). Other methods in this category make use of symbolic regression for predicting dynamical systems (Quade et al., 2016) or automatic model construction via Gaussian processes (Duvenaud, 2014). Recently, generative machine learning was combined with symbolic regression to enable efficient kinetic model discovery (Medina et al., 2026). Additionally, hybrid modeling approaches, where data-driven machine-learning methods are combined with models from first principles, have also gained increasing interest (Espinel-Ríos et al., 2024; Pennington et al., 2024; Sansana et al., 2021).

The second class of model identification approaches uses model ensembles to identify the model variant that best describes the observed data. For this purpose, all models of the ensemble need to be evaluated by simulating them, which in turn requires parameterization, highlighting the connection between parameter and model structure uncertainty. A basic modeling guideline states that models should only be as large and complex as necessary (*Occam's Razor*). Therefore, when comparing different model variants, a trade-off between model size and descriptive power needs to be considered. Model selection criteria such as Akaike's Information Criterion (Akaike, 1973) or the Bayesian Information Criterion (Schwarz, 1978) address this issue by introducing penalty terms that scale with the amount of model parameters to promote sparsity during model selection. An example of an ensemble-based model selection approach is the modelMAGE framework (Flöttmann et al., 2008; Schaber et al., 2011). There, a master model is assembled which contains all reactions and associated regulations, including hypothetical candidate reactions and mechanisms. A specific set of user-defined candidate submodels (derived from the master model) is then fitted against experimental data and the best model is selected according to the corrected Akaike Information Criterion for small sample sizes (Hurvich and Chih-Ling, 1989; Sugiura, 1978). Other ensemble-based methods use Bayesian techniques to evaluate and select the most plausible model. Examples include the extension of GRASP (General Reaction Assembly and Sampling Platform) to include model selection via Approximate Bayesian Computation (Matos et al., 2022; Saa and Nielsen, 2016) and Bayesian Model Comparison using Nested Sampling (Pullen and Morris, 2014).

Model identification methods of the third class start with an initial model candidate, that is refined by incorporating additional information from experimental data and/or from first principles. One example of this class is the Numerical Matrices Method for nonlinear system identification (Karnaukhov et al., 2007). In its most general form with no prior knowledge of the reaction dynamics, it starts with a comprehensive model structure that contains all possible uni- and bimolecular reactions for a set of known process species (metabolites and enzymes). Then, by integrating information on observed reaction rates it seeks to identify, in one step, the combination of reactions that best describes the data.

Comparing advantages and disadvantages of the different approaches, data-driven machine-learning methods require no or only some (in case of hybrid models) prior information and assumptions of mechanistic properties of the underlying process. However, they usually require significantly larger datasets and the constructed models are often difficult to interpret biologically. Moreover, typically only simple mechanisms and/or simple combinations of candidate mechanisms (e.g., linear combination in SINDy) are possible. Ensemble-based methods search a broader solution space of possible and biologically motivated model structures and can therefore significantly reduce model structure uncertainty. However, parameterization of each model of the ensemble is required to rank the different model variants, which introduces an additional source of uncertainty. Finally, attempting to improve a single initial model candidate can lead to a model that precisely describes the specific process under investigation. However, completely different alternative model structures that might also describe the observed dynamics are not considered.

In this work, we present MoRSel (model refinement and selection), an automated model selection approach for kinetic models that addresses many of the limitations described above. Our approach starts with a biologically plausible core model and uses a library of possible (candidate) model changes (addition or removal of reactions and/or modifications of kinetic rate laws), which are iteratively introduced into the core model with the goal to identify the model structure that achieves the best balance between fit quality and model size. MoRSel complements our previously developed method of robust ensemble-based optimization under parametric uncertainty (Huber et al., 2024): it provides a model selection module to handle structural uncertainties during the construction of the kinetic model, which is later used for robust process optimization (Fig. 1).

**Fig. 1:**
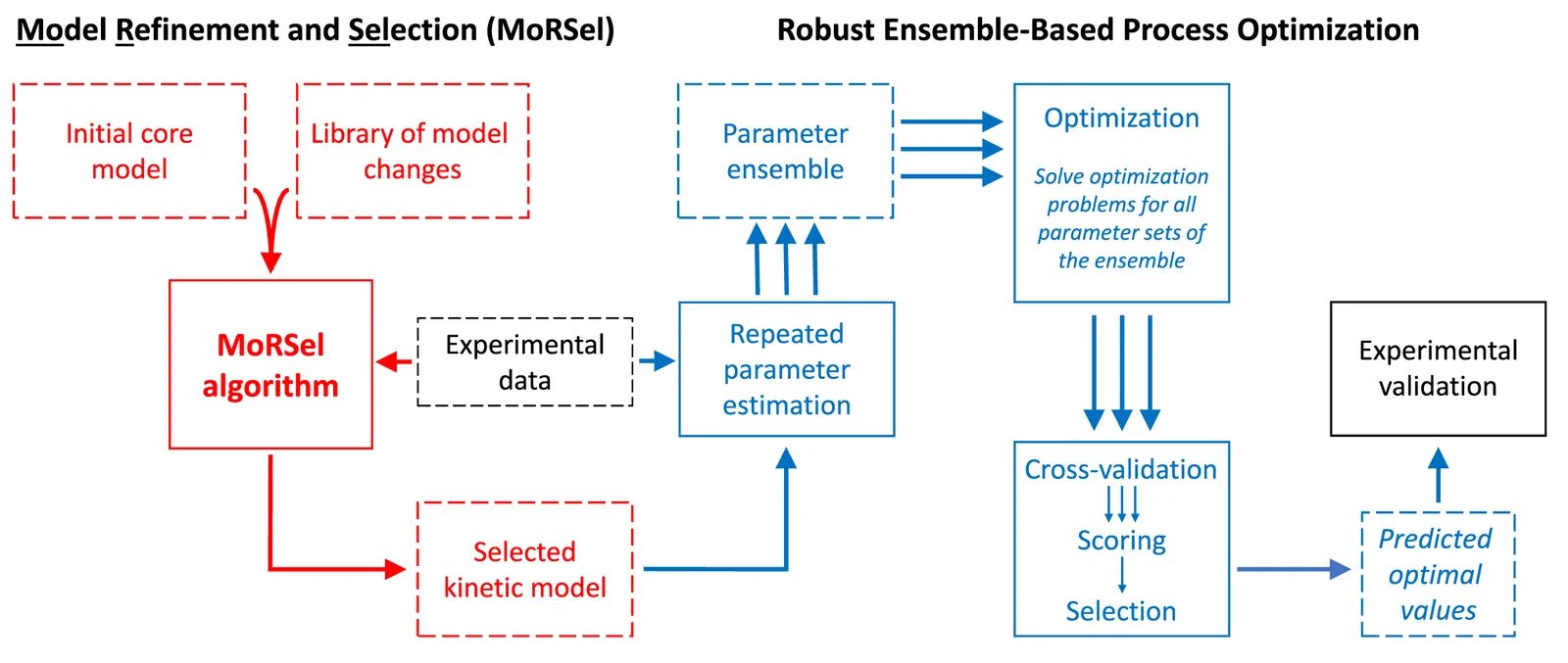
**Overview of the extended model-based optimization framework.** The original framework for robust process optimization (in blue) was presented by Huber et al. (2024). The new module developed in this work (MoRSel) is highlighted in red. An initial core model built from prior knowledge and first principles can be modified using a library of model changes. Different model variants are evaluated and ranked by the model selection algorithm using experimental time course data of the process. To account for parameter uncertainty, the kinetic parameters of all tested model variants are repeatedly estimated using a global estimation algorithm and the available experimental data. After model selection, the parameter ensemble of the selected model is further expanded and the defined optimization problem is solved for all parameter sets in the ensemble. Cross-validation and scoring of the optimization results identify the best-scoring prediction, which is then tested experimentally. The experimental steps (training data and validation) are depicted in black and the line type of the boxes differentiates between objects (dashed) and processes (solid).

To demonstrate the framework in Fig. 1 end-to-end, we applied it to modeling and optimization of a cell-free multi-enzyme cascade for the production of the nucleotide sugar UDP-GalNAc (Fig. 2). Nucleotide sugars are compounds where nucleoside di- or mono phosphates are bound to sugar molecules. In cells they serve as activated building blocks for the biosynthesis of polysaccharides, which in turn can be used to create glycoconjugates - compounds where proteins, peptides or lipids are covalently linked to glycans. Most of these glycoconjugates in mammals are built from only nine main monosaccharide subunits (del Val et al., 2016; Kightlinger et al., 2020) including *N*-acetyl-D-galactosamine (GalNAc), which, in its nucleotide sugar form, is bound to uridine diphosphate (UDP). Recent efforts to produce UDP-GalNAc were summarized by Frohnmeyer and Elling (2023), covering both cell-based and cell-free methods. Due to the importance of nucleotide sugars for the industrial production of therapeutic glycoproteins (Blondeel and Aucoin, 2018), glycoconjugate vaccines (Micoli et al., 2019), and human milk oligosaccharides (Walsh et al., 2020), we established a cell-free enzyme cascade for the production of UDP-GalNAc (shown in Fig. 2) in a previous work (Hoang et al., 2025). Apart from the main pathway synthesizing UDP-GalNAc from uridine and GalNAc, it includes a polyphosphate-based *in situ* ATP regeneration module previously used in other cell-free systems together with the inorganic pyrophosphatase (PPA) for degrading pyrophosphate, a potential inhibitor of the GLMU enzyme. Herein, we first built an initial kinetic model of the cascade, which, due to apparently missing components in the model structure, was refined and modified by applying MoRSel. Afterwards, we used the identified model to optimize the cell-free batch process with regard to different performance metrics (titer, yield, enzyme load).

**Fig. 2:**
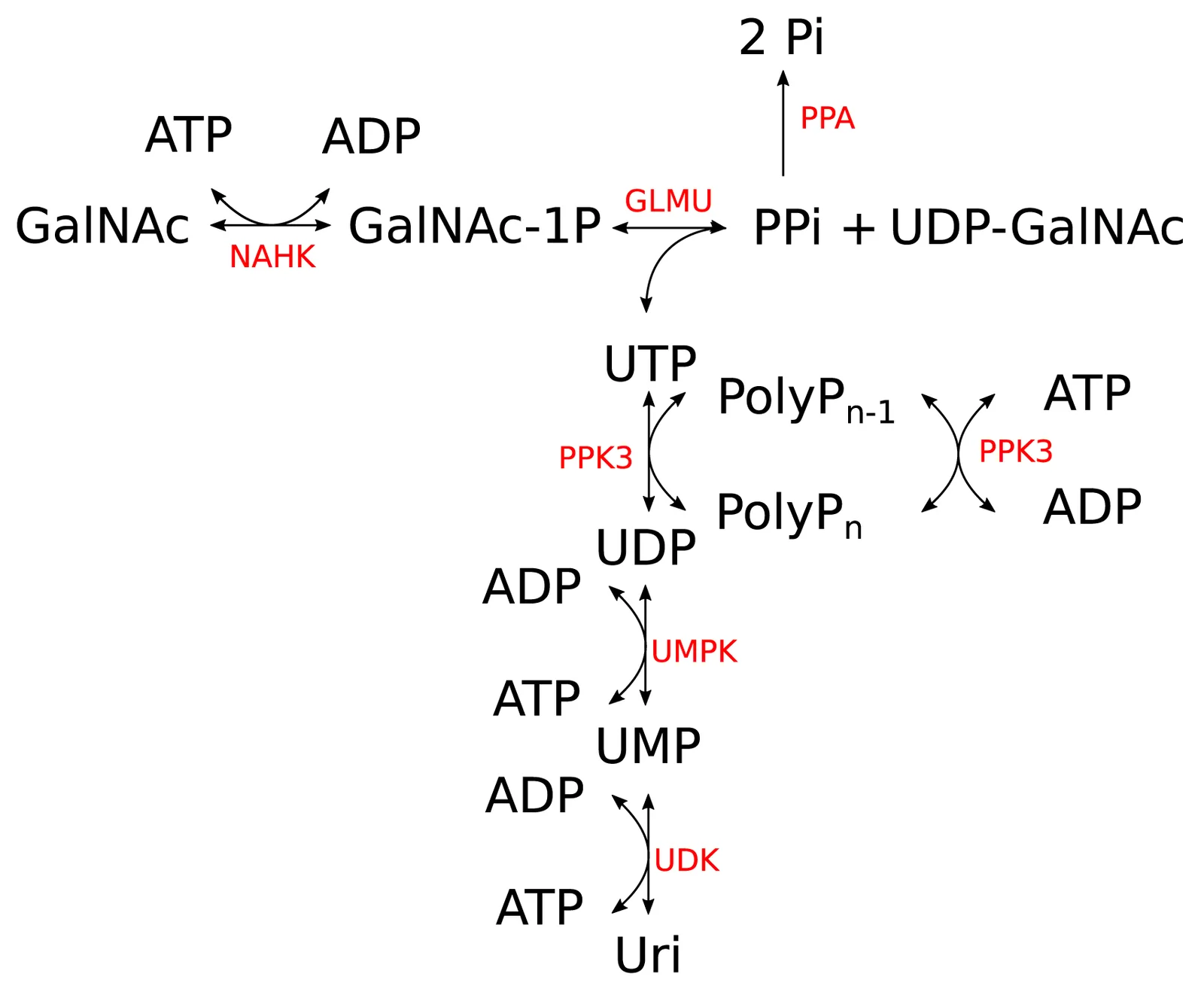
**Enzyme cascade for the production of UDP-GalNAc.** Enzymes are shown in red, metabolites in black. Abbreviations: GalNAc: N-acetylgalactosamine; GalNAc-1P: N-acetylgalactosamine-1-phosphate; ATP: adenosine triphosphate; ADP: adenosine diphosphate; PPi: inorganic pyrophosphate; Uri: uridine; UMP: uridine monophosphate; UDP: uridine diphosphate; UTP: uridine triphosphate; PolyP_n_: polyphosphate of chain length n; Pi: inorganic phosphate; NAHK (UniProt-ID E8MF12): N-acetylhexosamine 1-kinase; GLMU (UniProt-ID Q9CK29): bifunctional protein GlmU (N-acetylglucosamine-1-PO(4) uridyltransferase); PPA (UniProt-ID P57918): inorganic pyrophosphatase; UDK (UniProt-ID P0A8F4): uridine kinase; UMPK (UniProt-ID O04905): UMP-CMP kinase 3; PPK3 (UniProt-ID Q5LSN8): polyphosphate kinase.

## 2 Results

In the following, we first introduce our new kinetic model refinement and selection module (MoRSel). In the second part, we demonstrate its applicability as an initial module for the robust optimization of a cell-free multi-enzyme cascade for UDP-GalNAc synthesis (Fig. 1).

### 2.1 Development of a new model selection pipeline

MoRSel implements a new model selection approach based on a pipeline that starts from an initial kinetic (core) model, a library of (candidate) model changes (e.g., adding/removing reactions or changes in kinetic rate laws), and experimental datasets. It seeks to identify the model structure with the optimal balance between fit quality and model complexity. The pipeline starts with the construction of an initial core model, which usually contains all known reactions and their assumed kinetic rate laws of the system under study. The model is represented by a set of ordinary differential equations

(1) $$\begin{array}{c} \dot{x}\left(t\right)=f\left(x\left(t\right),t\right)=N\cdot r\left(x\left(t\right),P,t\right)\text{,} \end{array}$$

where x(t) is the concentration vector of all species (metabolites and enzymes), N the stoichiometric matrix of the reaction network, and r the vector of (net) reaction rates. The latter are defined by functions (rate laws) of the species concentrations x(t), reaction parameters P, and time t. For the rate laws of all enzyme-catalyzed reactions, we used the convenience kinetics formulation by Liebermeister and Klipp (2006), a generalized version of Michaelis-Menten kinetics, which we modified by integrating the Haldane relationship (leading to terms with the equilibrium constant of the respective reaction). Each rate law thus includes parameters for the affinities of participating compounds (Michaelis constants $K_{m}$), the turnover rate of the enzyme ($k_{cat}$), as well as the equilibrium constant of the respective reaction ($K_{eq}$). For example, for a reversible reaction catalyzed by an enzyme E that converts two substrates, $S_{1}$ and $S_{2}$, into two products, $P_{1}$ and $P_{2}$, the rate law would read (in the formula, E, $S_{1},S_{2},P_{1}$ and $P_{2}$ correspond to the respective concentrations):

(2) $$\begin{array}{c} \begin{array}{c} r=E\cdot \frac{k_{cat}^{+}\cdot \left(\frac{S_{1}}{K_{m,S_{1}}}\cdot \frac{S_{2}}{K_{m,S_{2}}}-\frac{1}{K_{eq}}\cdot \frac{P_{1}\cdot P_{2}}{K_{m,S_{1}}\cdot K_{m,S_{2}}}\right)}{\left(1+\frac{S_{1}}{K_{m,S_{1}}}\right)\cdot \left(1+\frac{S_{2}}{K_{m,S_{2}}}\right)+\left(1+\frac{P_{1}}{K_{m,P_{1}}}\right)\cdot \left(1+\frac{P_{2}}{K_{m,P_{2}}}\right)-1} \end{array}\;\text{.} \end{array}$$

$k_{cat}^{+}$ is the turnover rate of the forward reaction. Known regulatory interactions between metabolites and enzymes can be included via suitable terms in the rate laws. Generally, the core model should contain all (and only) the reactions and regulatory terms that are evident from biological knowledge; these are consequently treated as *ground truth*.

Initial fitting (parameter estimation) of the core model to experimental data may fail to adequately describe the dynamics of the given enzyme cascade. One typical reason for this is that the initial core model does not include all relevant regulatory interactions between metabolites and enzymes. Such regulatory effects may become relevant due to the mixing of all compounds in a one-pot process at concentrations that may exceed physiological values in cells. For similar reasons, unknown or unconsidered side reactions may occur and affect the overall dynamics of the cascade. Therefore, if the fitted initial core model does not adequately describe experimental data, the next step is to collect information on potential allosteric regulations and/or side reactions of the participating enzymes, resulting in a library of model changes. These model changes are thus either *stoichiometric* (addition or deletion of entire reactions, including their rate laws) or *kinetic* (changes in the respective rate law of the reaction). Regarding stoichiometric changes, all reactions (including those not in the core model) are already present in the stoichiometric matrix N**,** and implementing a stoichiometric change is equivalent to setting the corresponding rate laws either to zero or to the (new) rate law equation. Kinetic changes affect the rate law of a reaction in one of three ways: (a) by replacing it entirely by a new mechanistic description, (b) by incorporating competitive inhibition terms between different reactions catalyzed by the same enzyme, or (c) by combining the existing rate law with multiplicative terms related to allosteric enzyme regulation. Herein, for integrating allosteric inhibition, we multiply the rate law of the respective reaction rate $r_{i}$ with the term $\frac{K_{i}}{K_{i}+I}$ ,where $K_{i}$ is the inhibitory constant and I is the concentration of the metabolite acting as an inhibitor. For allosteric activation terms, we use the factor $\left(1+\left(K_{a}\cdot A\right)\right)$ where $K_{a}$ is the activation constant and A is the concentration of the metabolite acting as an activator. Since $K_{i}$ and $K_{a}$ are free parameters (to be estimated during model fitting), it is always possible to restore the behavior of the model before the inclusion of a regulatory term by choosing very high values for $K_{i}$ and zero for $K_{a}$. Hence, it is ensured that the best fit of a model structure modified via allosteric terms cannot be worse than that of a previous model structure when a regulation term is introduced. The same holds true when adding new reactions because the value of their $k_{cat}$ parameter can be set to 0 to restore the previous dynamics. Only the removal of reactions may lead to a worse best model fit.

Given an initial model and a list of possible model changes, our model selection algorithm aims to identify a sparse set of modifications to the model that improves the goodness of its fit as an indicator of its predictive power. Model fitting is carried out using a least-squares approach with the objective to minimize the residual sum of squares (RSS) between model simulation and experimental data:

(3) $$\begin{array}{c} \min\;\left\{\text{RSS}=\sum_{k}\sum_{i,j}\omega_{j,k}\cdot {\left(y_{i,j,k}-x_{i,j,k}\left(P\right)\right)}^{2}\right\}\text{,} \end{array}$$

where $y_{i,j,k}$ is the concentration of species j at time point i in experiment k and $x_{i,j,k}\left(P\right)$ is the concentration of the corresponding model species at the same time point, derived from a simulation of the model (based on a parameter set P and with initial concentrations used in experiment k). $\omega_{j,k}$ is the normalized weight of species j for experiment k and is calculated with the following equation:

(4) $$\begin{array}{c} \omega_{j,k}=\frac{1/\left\langle \;y_{j,k}^{2}\;\right\rangle }{\max_{j\in \left\{1\ldots n_{j}\right\}}1/\left\langle \;y_{j,k}^{2}\;\right\rangle }\text{.} \end{array}$$

Here, $\left\langle \;y_{j,k}^{2}\;\right\rangle$ expresses the mean square of all measurements y of species j across all time points of experiment k, and $n_{j}$ is the number of species measured in experiment k.

Even if the optimal parameter set minimizing the RSS were found, the RSS alone is not suitable for comparing models with different numbers of parameters. In particular, adding regulatory terms or reactions (and their parameters) to a model implies that, as explained above, a parametrization always exists that can at least reproduce the model behavior before introducing the modification (i.e., the RSS cannot become worse). To correct for this effect and to promote sparsity, the quality of a given model structure (i.e., its fitness) is evaluated by computing model selection criteria (MSC), which penalize the number of model parameters as an indicator of model complexity. An overview of the MSC used in this work is given in Table 1. Since MSC are originally defined based on a maximum likelihood estimation approach, we are using the reformulation for least-squares parameter estimations derived by Portet (2020), which is applicable if the measurement errors in the data are independent and identically as well as normally distributed with the same variance.

**Table 1:** List of model selection criteria (MSC). The log-likelihood terms used in the original criteria were reformulated for a least-squares estimation according to Portet (2020). RSS is the residual sum of squares of the parameter estimation, n is the number of experimental data points and k is the number of estimated parameters + 1. Generally, a model with a smaller MSC is considered better. In this study, the weighting factors were set to α=0.01 and β=200.

Name	Abbreviation	Equation	Reference
Akaike Information Criterion	AIC	$AIC=n\cdot \;\ln\left(\frac{RSS}{n}\right)+2\cdot \;k$	Akaike (1973)
Corrected Akaike Information Criterion	AICc	$AICc=n\cdot \;\ln\left(\frac{RSS}{n}\right)+\frac{2\cdot \;k\cdot \;n}{\;n-k-1}$	Sugiura (1978), Hurvich and Chih-Ling (1989)
Bayesian Information Criterion	BIC	$BIC=n\cdot \;\ln\left(\frac{RSS}{n}\right)+\ln\left(n\right)\cdot k$	Schwarz (1978)
Custom Information Criterion 1	CIC1	$CIC1=\left\{ \begin{array}{c} n\cdot \;\ln\left(\frac{RSS}{n}\right)\left(1+\frac{\alpha }{k}\right)\;\left(if\;\ln\left(\frac{RSS}{n}\right)\leq 0\right)\\ n\cdot \;\ln\left(\frac{RSS}{n}\right)\left(1+\alpha \cdot k\right)\;\left(if\;\ln\left(\frac{RSS}{n}\right)>0\right) \end{array}\right.$	This study.
Custom Information Criterion 2	CIC2	$CIC2=n\cdot \;\ln\left(\frac{RSS}{n}\right)+\beta \cdot k$	This study.
Custom Information Criterion 3	CIC3	$CIC3=\left\{ \begin{array}{c} n\cdot \;\ln\left(\frac{RSS}{n}\right)\cdot \frac{1}{k}\;\left(if\;\ln\left(\frac{RSS}{n}\right)\leq 0\right)\\ n\cdot \;\ln\left(\frac{RSS}{n}\right)\cdot k\;\left(if\;\ln\left(\frac{RSS}{n}\right)>0\right) \end{array}\right.$	This study.

A commonly used model selection criterion, which is based on information-theoretical considerations, is the Akaike Information Criterion (AIC) (Akaike, 1973). From a given set of different models, it aims to find the model that explains the data with the least loss of information. Assuming a large sample size (number of data points) n, a penalty term of 2k is included in the criterion to account for the number of model parameters k (Table 1). A corrected version of the AIC (AICc) was derived by Sugiura (1978) and Hurvich and Chih-Ling (1989). Compared to the traditional AIC, it features a more complex penalty term, imposing a heavier penalty for smaller n but approaching 2k when n grows larger, making the corrected AIC more robust for smaller sample sizes. Neither the AIC nor the AICc assumes that the true model is included in the set of models to be tested. This contrasts with the Bayesian Information Criterion (BIC) (Schwarz, 1978), which selects the model that maximizes its posterior probability given the available data, assuming the true model is among the set of different models variants. Similar to the AICc, but unlike the AIC, the penalty term of the BIC grows with the sample size n.

According to our experience, the effect of the penalty terms of AIC, AICc, and BIC is sometimes too low, leading to the selection of larger models even if they achieve only minor improvements in the fit. To test the impact of penalty terms that scale more severely with the number of model parameters, we consider herein three additional heuristic custom information criteria: (1) CIC1 with an adaptive penalty term that scales with the magnitude of the fitting error (here, one needs to differentiate based on the sign of the term $\ln\left(\frac{RSS}{n}\right)$), (2) CIC2 that uses the AIC penalty term but with a much stronger weight, and (3) CIC3, which uses a single term containing the fitting error and the number of parameters. These custom criteria are not rooted in any theoretical framework but were instead derived as variations of the basic structure of the traditional MSC (a combination of a log-likelihood term and a penalty term), which proved useful in our applications.

Fig. 3 shows the workflow of MoRSel, which makes use of the concepts introduced so far. The algorithm starts by evaluating the goodness of the initial core model. Generally, herein, the goodness of a model variant refers to computing and then aggregating all six MSC from Table 1 using the best-fitting parameter set (lowest RSS) of a parameter ensemble generated by repeating a least-squares parameter estimation with the respective model variant. When comparing different model variants, we rank them separately for each MSC and then calculate the overall mean rank to reduce all six MSC values to a single measure.

**Fig. 3:**
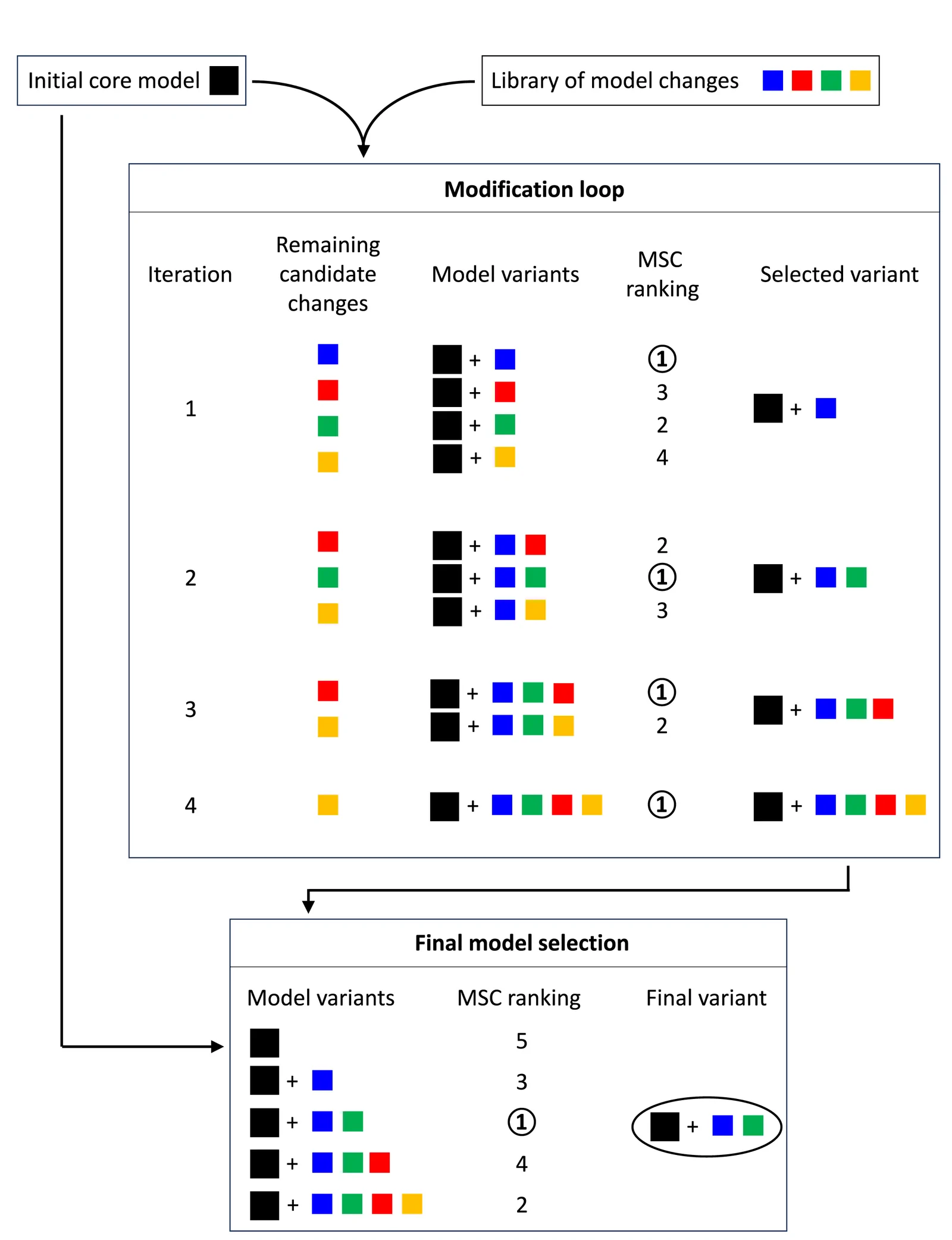
**Workflow of the proposed model refinement and selection algorithm (MoRSel).** The first iteration starts with the initial core model, where each candidate model change is separately integrated and the resulting model variant evaluated. The model variant with the best aggregate MSC ranking becomes the new model, and the introduced model change is removed from the library. The loop stops when all candidate model changes have been integrated. In this example, the library contains four candidate model changes. A total of 11 out of 16 possible model variants (including the core model) are evaluated during the modification loop across four iterations. During the final model selection, the best variant of each iteration and the initial core model are ranked to identify the overall best model variant.

After evaluating the initial core model, the first round of the iterative modification loop of the model refinement and selection algorithm begins. In its first iteration, each candidate from the library of model changes is individually integrated into the initial core model. For each of these generated model variants, several parameter estimation runs are performed (the parameters are initialized with the values of the best-fitting parameter set of the initial core model ensemble), which serve to assess the goodness of the model variants. Following a greedy strategy, among all model variants tested, the one with the highest goodness is used as the new core model for the subsequent iteration. It is also stored for a final ranking step (performed after the loop has concluded), and the model change introduced in this selected model variant is removed from the library. Additionally, the values of the best-fitting parameter set generated during the evaluation of the selected model variant are stored and used to initialize the parameter estimations performed in the next iteration.

The second and all subsequent iterations repeat the steps of the first, using the current (modified) model variant and the reduced library. Thus, in each iteration, many more model variants are evaluated than are stored in the final output list, which collects the selected best model variant from each iteration. The modification loop stops when no model changes are left in the library. A final model selection step then ranks all stored model variants (including the initial core model) based on the aggregated MSC metrics. This time, both the mean and the median rank are computed across all MSC values. If a model variant achieves both the best mean and median rank, it is directly selected. If the best mean- and median-ranked model variants differ, the sparser one is selected (i.e., the one with fewer model parameters). A new ensemble of parameter sets is computed for the selected final model variant, which is used for the subsequent (robust) optimization steps in the pipeline shown in Fig. 1.

We implemented MoRSel as a Python-based program that utilizes functions for model building, time course simulation, and parameter estimation from COPASI (Hoops et al., 2006) through its Python API, BasiCO (Bergmann, 2023). The program, together with all scripts, data, and models used herein, is available at the repository https://github.com/klamt-lab/MoRSel_UDP-GalNAc_Paper and archived at https://doi.org/10.5281/zenodo.19098183.

### 2.2 Testing MoRSel with an example model and synthetic data

We used the kinetic toy model shown in Fig. 4 to test whether MoRSel can "rediscover" the true model structure and dynamics from a given (incomplete) core model, a library with five potential model changes, and data generated from simulating the true model. The true model consists of a substrate S, a product P, and an inhibitor X. S is converted to P by reaction 1, which is inhibited by X. X itself is produced from S and P by reaction 4. The ODEs, reaction rate laws (for sake of simplicity, we use mass-action kinetics), and all parameter values of this toy model are shown in Fig. 4. We simulated the true model (with $S_{0}=10$; $X_{0}=P_{0}=0$ and all kinetic parameters set to 1) to generate time course data, to which we added Gaussian noise. We then constructed an initial (incomplete) core model, which only included reaction 1, assuming it was the only reaction known to be part of the system. Experimental tests would then reveal that a compound X appears in the process, which cannot be explained by the initial core model alone. We provided a library of candidate model changes containing three new reactions representing different hypotheses for the production of X (reactions 2, 3, and 4; the latter being part of the true model) and two candidate regulatory mechanisms (X as either an activator or an inhibitor of reaction 1; the latter again being part of the true model).

**Fig. 4:**
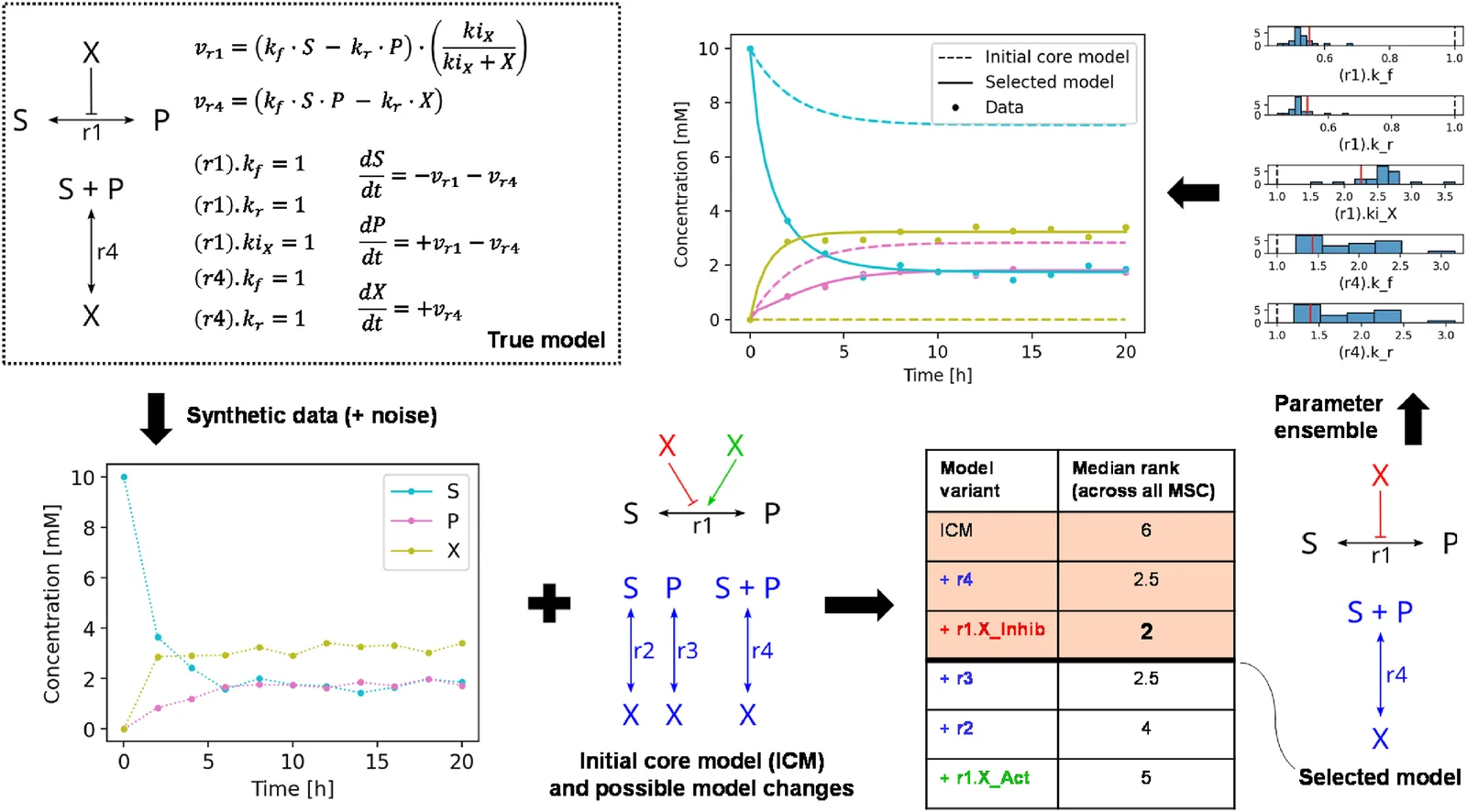
**Overview of the rediscovery test with a simple toy model**. Synthetic time course data with a 2-h step were generated by simulating the true model. Gaussian noise (mean: 0, standard deviation: 0.2) was added to the data, which were then used as input for MoRSel, along with an initial (incomplete) core model and a library of five potential model changes. Model selection results show that model variant 3 (the initial core model with the addition of reaction 4 and the allosteric inhibition of reaction 1 by X) best describes the synthetic data, as it achieves the best median rank across all model selection criteria (MSC). Then, a parameter ensemble was calculated for the selected model variant by repeating a least-squares estimation. The parameter histograms show that the estimated values of the optimal parameter set of the ensemble (red lines) are close to the true parameter values (black dashed lines). Also, the time course simulation of the selected model (solid lines) closely matches the synthetic data points, unlike the initial core model (dashed lines). This is to be expected, as both the model structure and the kinetic parameter values were identified successfully.

Applying MoRSel, as described in the previous section, leads to the successful identification of the true model structure. Additionally, the parameter values of the best fit of the parameter ensemble calculated for the identified model structure are close to their true values. Accordingly, the time course simulations of the selected model are much closer to the data compared to the initial core model (Fig. 4).

### 2.3 Using MoRSel to improve a model for the cell-free UDP-GalNAc production system

We applied our model selection pipeline to systematically improve an initial kinetic model describing the dynamics of the cell-free enzyme cascade producing UDP-GalNAc from uridine and GalNAc (see Fig. 2). As the first step, an initial model of the UDP-GalNAc cascade was constructed (see Materials and Methods). We then performed 12 experiments with different initial substrate and enzyme concentrations and measured the time course of metabolites of the cascade (Supplementary File S1). Analysis of the mass balances of the metabolite pools related to adenosine and uridine, respectively, showed only small violations across all experiments (mean deviations of 5.68 % and 0.54 % respectively).

We then fitted the initial core model to these data and found larger discrepancies between the available measurements and the simulations of the fitted core model (Supplementary File S2). This prompted us to compile a list of potentially relevant model changes (Fig. 5, Table 2), which were considered plausible and relevant to improve the model fit of the initial model. The model changes include several potential enzyme regulations (kinetic changes), for which some hints can be found in the literature (Table 2). The library also includes new reactions, which are not directly based on literature information but on inspection of the experimental data. In particular, the core model did not include any reaction that could produce adenosine monophosphate (AMP), adenosine tetraphosphate (ATPP), or adenosine pentaphosphate (ATPPP), although these species appeared with relevant concentrations in all experiments (Supplementary File S1). We therefore included different candidates for possible stoichiometric model changes that enable the formation of AMP, ATPP, and/or ATPPP (the latter two were grouped in the model as one variable: ATPP). One possible explanation for the accumulation of AMP observed in all experiments could be a spontaneous hydrolysis of ADP, leading to the loss of a phosphate group and thereby the formation of AMP. Therefore, two different types of ADP decay reactions with simple mass action (instead of enzyme-kinetic) rate laws were added to the library of model changes. However, it is also possible that AMP is created by kinase reactions (such as UDK, UMPK, and NAHK) if they use adenosine diphosphate (ADP) as a phosphate donor instead of ATP. The formation of adenosine nucleotides with more than three phosphate groups (summarized in the ATPP model variable) could be the result of PPK3 catalyzing the transfer of additional phosphate groups onto ATP. This additional side reaction was also included in the library of model changes, alongside alternative versions of all kinase reactions, enabling them to use ATPP as a phosphate donor, resulting in the production of ATP. If additional enzyme-catalyzed (side) reactions are added to the model, it is ensured that necessary competitive inhibition terms are included in the rate laws of all reactions catalyzed by the same enzyme.

**Fig. 5:**
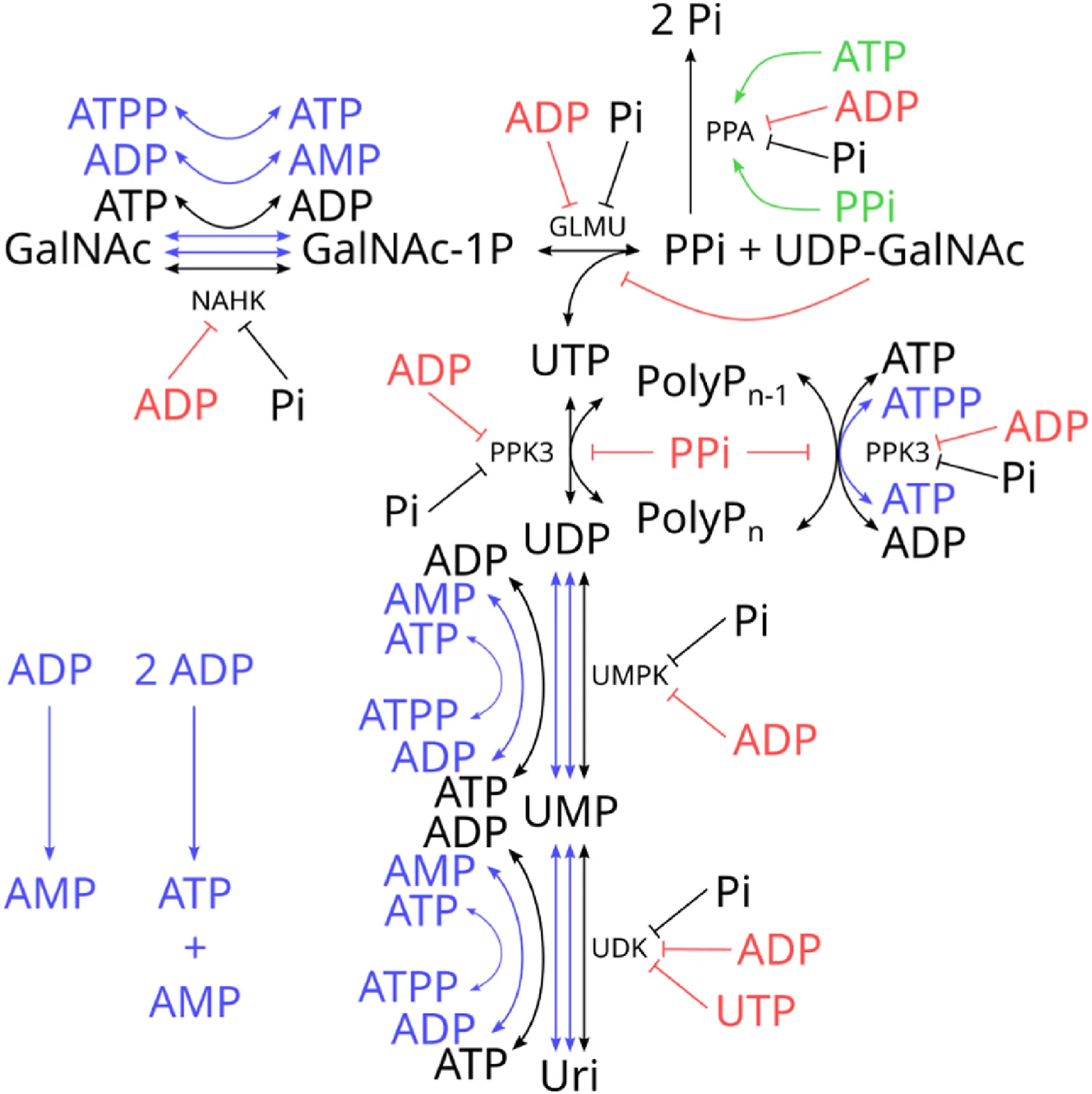
**Network graph of the initial core model with all candidate model changes contained in the library.** Black: initial core model (compare with Fig. 2), blue: stoichiometric candidate changes (addition of reaction variants or new reactions), red/green: kinetic candidate changes (addition of inhibitory/activating enzyme regulations). The library of model changes is also listed in Table 2.

**Table 2:** **Library of possible model changes for the kinetic model of the UDP-GalNAc system.** Candidate changes without a literature reference are hypotheses based on biochemical knowledge and evaluation of the available experimental data. See also Fig. 5 and text for further explanations.

ID	Reaction	Description of candidate model change	Reference
1	ADP Decay Version 1	Addition of a new reaction: ADP → AMP + P	Hypothesis (this study)
2	ADP Decay Version 2	Addition of a new reaction: ADP + ADP → ATP + AMP	Hypothesis (this study)
3	GLMU	Enzyme regulation (ADP Inhibition)	Hypothesis (this study)
4	GLMU	Enzyme regulation (UDP-GalNAc Inhibition)	Mengin-Lecreulx and van Heijenoort (1994)
5	NAHK (ADP)	Addition of a new reaction: GalNAc + ADP ⇌ GalNAc1P + AMP	Hypothesis (this study)
6	NAHK (ATPP)	Addition of a new reaction: GalNAc + ATPP ⇌ GalNAc1P + ATP	Hypothesis (this study)
7	NAHK (ADP and ATPP)	Combination of 5 and 6	Hypothesis (this study)
8	NAHK (all substrates)	Enzyme regulation (ADP Inhibition)	Hypothesis (this study)
9	PPA	Enzyme regulation (ADP Inhibition)	Hypothesis (this study)
10	PPA	Enzyme regulation (ATP Activation)	Rodina et al. (2007)
11	PPA	Enzyme regulation (PP Activation)	Chao et al. (2006)
12	PPK3 (ATP)	Addition of a new reaction: ATP + PolyP ⇌ ATPP	Hypothesis (this study)
13	PPK3 (all substrates)	Enzyme regulation (ADP Inhibition)	Hypothesis (this study)
14	PPK3 (all substrates)	Enzyme regulation (PP Inhibition)	Tzeng and Kornberg (2000)
15	UDK (ADP)	Addition of a new reaction: Uri + ADP ⇌ UMP + AMP	Hypothesis (this study)
16	UDK (ATPP)	Addition of a new reaction: Uri + ATPP ⇌ UMP + ATP	Hypothesis (this study)
17	UDK (ADP and ATPP)	Combination of 15 and 16	Hypothesis (this study)
18	UDK (all substrates)	Enzyme regulation (ADP Inhibition)	Hypothesis (this study)
19	UDK (all substrates)	Enzyme reglation (UTP Inhibition)	Valentin-Hansen (1978)
20	UMPK (ADP)	Addition of a new reaction: UMP + ADP ⇌ UDP + AMP	Hypothesis (this study)
21	UMPK (ATPP)	Addition of a new reaction: UMP + ATPP ⇌ UDP + ATP	Hypothesis (this study)
22	UMPK (ADP and ATPP)	Combination of 20 and 21	Hypothesis (this study)
23	UMPK (all substrates)	Enzyme regulation (ADP Inhibition)	Hypothesis (this study)

Using the initial model and the library of potential model changes, we repeated the entire MoRSel framework five times using data from 12 different experiments (see Supplementary File S1 for detailed information on the different initial conditions). The five runs were computed in parallel, requiring a total computation time of 4 days and 19 h on a High-Performance Computing cluster (CPU: 2x Intel Xeon Silver 4110, 8 Cores per CPU, 2.1 GHz, RAM: 192 GB), and the results are summarized in Table 3. The five runs identified different model variants, due to parameter uncertainty and because a parameter estimation algorithm with stochastic elements was used. However, significant overlaps and shared patterns between the selected model variants of each run can be seen. Additionally, we performed test runs where different subsets of MSC were used during the MoRSel calculation (e.g., only AIC or a combination of AIC, AICc, and BIC). These runs show that the choice of the model selection criteria affects the structure of the selected model variant, with AIC, AICc, and BIC (as well as their combination) clearly favoring larger models, which motivated the introduction of the three custom information criteria The selected model variants of the five main runs were each ranked for all MSC, and their mean and median ranks were calculated. In this way, the model selected by run 3 was identified as the overall best final model variant (Fig. 6). It consists of 52 kinetic parameters (initial core model: 43), and the best parameter set of its ensemble reaches an RSS of 0.951 (initial core model: 3.881). It achieves the best mean and median MSC ranks among all of the main MoRSel runs, whereas the initial core model ranks at the bottom in both categories.

**Table 3:** **Overview of all MoRSel runs.** For the selected model variant of each run, a parameter ensemble (n = 50) was calculated (k: number of (estimated) parameters). The model selection criteria (MSC) were calculated based on the parameter set with the lowest RSS (residual sum of squares). All five runs were ranked based on their MSC values. Run 3 is highlighted as it achieves the best mean and median rank. The selected model changes are referred to by the numbers assigned to them in Table 2. Runs 6-12 were additional test runs where only some of the MSC were used during the MoRSel calculation (6: AIC; 7: AICc; 8: BIC; 9: CIC1; 10: CIC2; 11: CIC3; 12: AIC, AICc, BIC). ICM: initial core model.

Run ID	k	RSS	AIC	AICc	BIC	CIC1	CIC2	CIC3	MSC Mean Rank	MSC Median Rank	Order of Selected Model Changes
ICM	43	3.881	−5990.9	−6075.1	−5771.6	−3404.2	2721.1	−138.2	4.5	5	-
1	52	0.968	−7472.9	−7573.3	−7208.7	−3562.1	3021.1	−143	3.17	3.5	1, 12, 11, 19, 18
2	53	0.987	−7449.8	−7552	−7180.7	−3476.6	3242.2	−140	4.17	4.5	1, 12, 23, 11, 10, 8
**3**	**52**	**0.951**	**−7491.5**	**−7591.9**	**−7227.3**	**−3570.8**	**3002.5**	**−143.4**	**2.17**	**2.5**	**2, 12, 19, 10, 3**
4	58	0.793	−7675.5	−7786.6	−7381.4	−3195.3	4006.5	−132.1	3	3	22, 19, 10, 11
5	60	0.799	−7663.4	−7777.9	−7359.3	−3036.3	4414.6	−127.6	4	4	22, 4, 11, 19, 18, 9
6	98	0.795	−7593.2	−7771	−7099.7	−77.91	12008.8	−78.7	-	-	22, 1, 6, 15, 18, 2, 9, 10, 11, 4, 3, 19, 7, 13, 12, 8, 14
7	93	0.774	−7631.9	−7801.7	−7163.3	−469.19	10980.2	−83.2	-	-	22, 15, 11, 3, 10, 17, 19, 8, 5, 14, 4, 9, 7, 23, 6, 13, 18
8	77	0.807	−7619.6	−7763.2	−7230.7	−1710.6	7824.45	−99.7	-	-	22, 12, 10, 11, 4, 18, 19, 9, 8, 3, 13, 14, 23, 20, 7, 6
9	49	0.999	−7444.8	−7539.8	−7195.5	−3772.4	2455.22	−150.9	-	-	2, 12
10	49	1.014	−7428.6	−7523.6	−7179.4	−3764.3	2471.41	−150.6	-	-	1, 12
11	49	1.003	−7440.3	−7535.4	−7191.1	−3770.2	2459.68	−150.8	-	-	1, 12
12	78	0.732	−7722.4	−7867.8	−7328.6	−1654.9	7919.61	−99.8	-	-	22, 15, 11, 19, 8, 10, 3, 6, 9, 4, 13

**Fig. 6:**
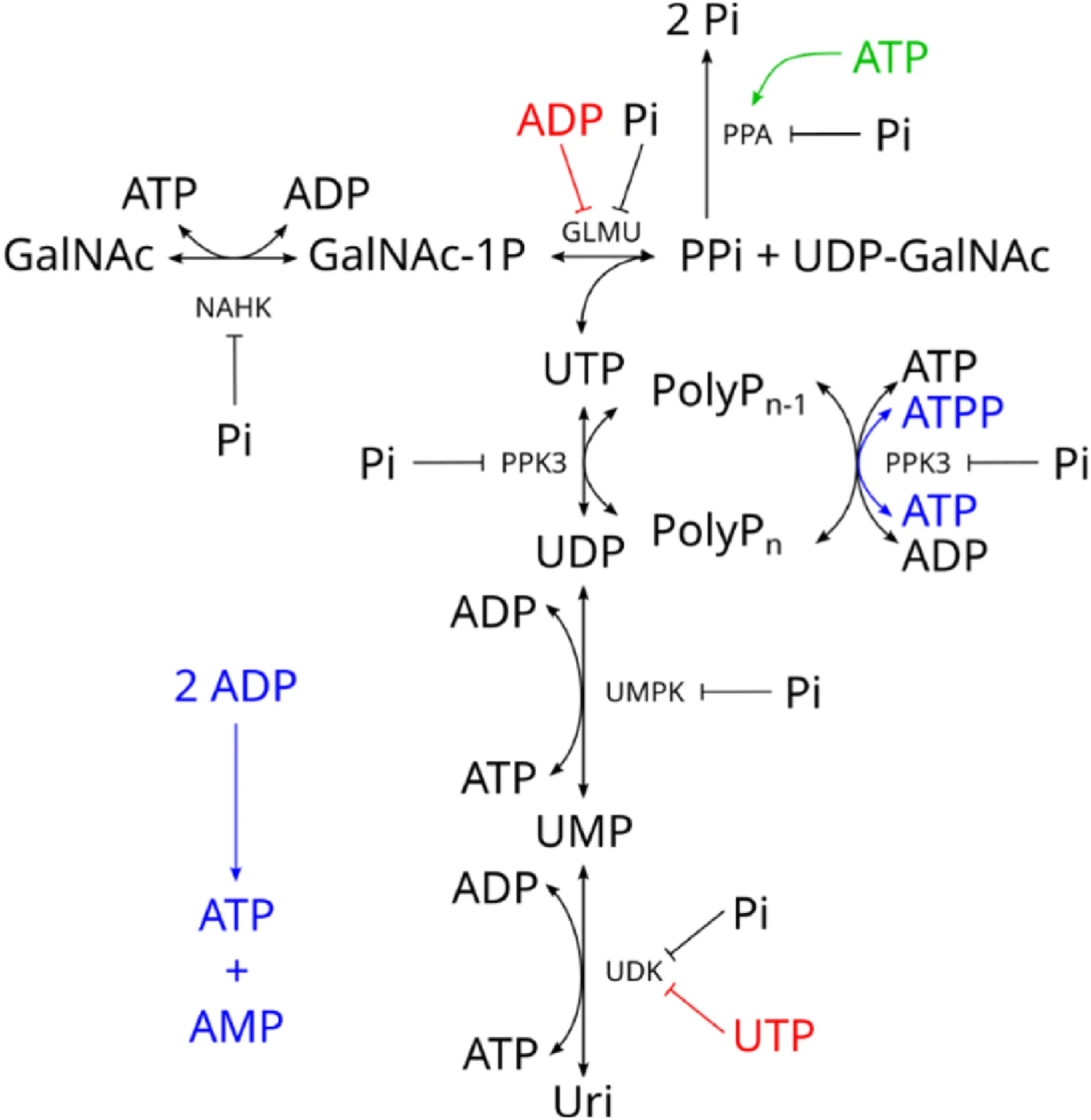
**Network map of the selected final model variant**. Black: initial core model, blue: selected stoichiometric model changes (additions of reactions), red/green: selected kinetic model changes (red: enzyme inhibitions, green: enzyme activations).

A total of five candidate model changes (two stoichiometric and three kinetic ones) were included in the selected model variant in the following order (Fig. 6): ADP Decay Version 2, PPK3 reaction variant using ATP as substrate, UTP inhibition of UDK, ATP activation of PPA, and ADP inhibition of GLMU. It is reasonable that the two stoichiometric model changes, ADP Decay Version 2 and PPK3 (ATP), were added first because a mechanism for producing AMP and ATPP was apparently needed for the model to better explain the data. The two kinetic model changes introduced subsequently (UTP inhibition of UDK and ATP activation of PPA) were described in the literature, and therefore their inclusion also aligns with our expectations. The last model change included (ADP inhibition of GLMU) was originally added to the library as a hypothesis based on analyzing the observed dynamics of the available experimental data.

A new ensemble of 50 parameter sets was calculated for the selected final model variant. Fig. 7 shows the differences in the simulated dynamics between the initial core model and the final model variant with respect to one selected experimental data set (figures showing the fit to all experimental data sets can be found in Supplementary File S2). Although the fit of the selected model variant against the experimental data is still not perfect, it is significantly better than that of the initial core model. Most notably, uridine and UDP-GalNAc (the target product), as well as AMP and ATPP, align much better with the observed data in the final model variant compared to the initial core model. Smaller improvements are also visible for other species. The significance of these improvements is especially highlighted in regions where there is no overlap of the 95 % confidence intervals. The parameter ensemble of the final model variant was then used for all subsequent optimization calculations.

**Fig. 7:**
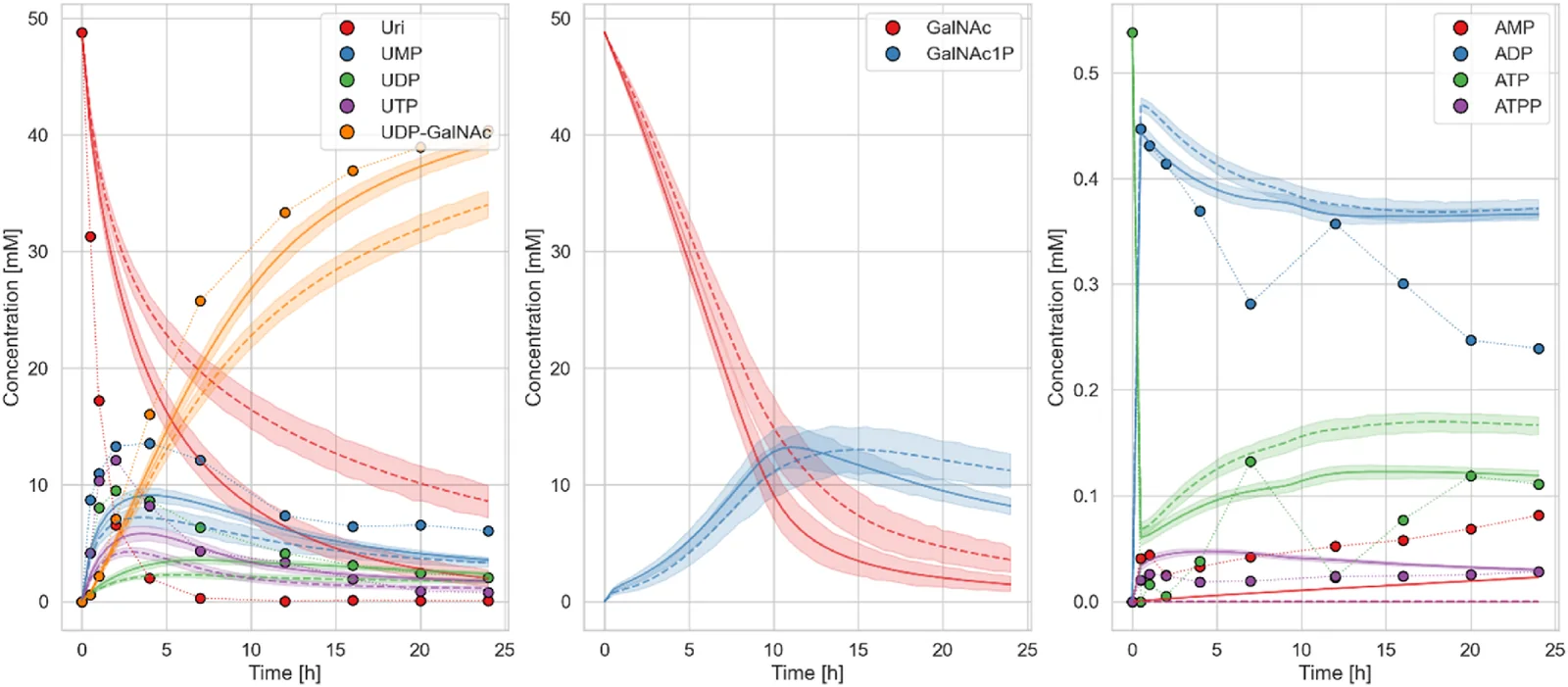
**Comparison of the model fits of the initial core model (dashed lines) and the selected model variant (solid lines) for experiment 4.** Trajectories show the average behavior of the ensemble generated for the respective model (n = 50) across all parameter sets; shaded regions show the 95 % confidence interval. The data points are from experiment 4 as listed in Supplementary File S1 (Configuration 36, 50 mM). This experiment was chosen because it is used as the (unoptimized) baseline behavior for process optimization (see below). Comparisons of model fits for all other experiments can be found in Supplementary File S2.

### 2.4 Model-based optimization of the UDP-GalNAc enzyme cascade under parametric uncertainty and experimental validation

We used the parameter ensemble of the selected final model variant to improve the performance of the cell-free UDP-GalNAc batch process compared to a defined baseline behavior (see Supplementary File S1; experiment 4; see also Fig. 7) by applying our methodology of optimization under parametric uncertainty developed in Huber et al. (2024) (see also Fig. 1). The approach consists of three steps: (1) the optimization problem is defined and solved for each parameter set of the ensemble (for this step, we used the genetic algorithm implemented in COPASI), (2) additional model simulations are computed to test how each optimization result performs across all remaining parameter sets in the ensemble (cross-validation), and (3) the performance of each optimization result is scored, and the best result is selected for experimental validation. Two economically relevant process objectives were considered, as outlined below.

#### 2.4.1 Optimization of the UDP-GalNAc titer

The objective of the first model-based optimization problem (Equations (5a), (5b), (5c), (5d), (5e)) is to identify initial enzyme and substrate concentrations $x_{E}^{0}$ and $x_{S}^{0}$ (decision variables) that maximize the concentration of the product UDP-GalNAc after 7 h. The problem is constrained by a maximal enzyme load $E_{tot,\max}$, which is set to that of the baseline experiment (experiment 4 in Supplementary File S1; see also Table 4). Initial enzyme and substrate concentrations are constrained by lower and upper bounds lb and ub, which are specific for all enzymes $i=1,\ldots ,n_{E}$ and all substrates $j=1,\ldots ,n_{S}$, and were derived from physical and practical considerations (e.g., solubility). The selected kinetic model variant is included as a constraint in the optimization problem, with initial concentrations denoted as $x\left(t_{0}\right)=x^{0}$ ($x_{E}^{0},x_{S}^{0}$ are included in $x^{0}$). The start values for the concentrations $x^{0}$ in the optimization were set to the initial concentrations of the baseline experiment. The overall optimization problem reads:

(5a) $$\begin{array}{c} \max_{x_{E}^{0},x_{S}^{0}}\;x_{UDP-GalNAc}\left(t=7\;h\right)\text{,} \end{array}$$

s.t.

(5b) $$\begin{array}{c} \dot{x}\left(t\right)=f\left(x\left(t\right),t\right),\forall t\in \left[t_{0},t_{end}\right],x\left(t_{0}\right)=x^{0}\text{,} \end{array}$$

(5c) $$\begin{array}{c} {lb}_{E_{i}}\leq x_{E_{i}}^{0}\leq {ub}_{E_{i}},\forall \;i=1,\ldots ,n_{E}\text{,} \end{array}$$

(5d) $$\begin{array}{c} {lb}_{S_{j}}\leq x_{S_{j}}^{0}{\leq ub}_{S_{j}},\forall \;j=1,\ldots ,n_{S}\text{,} \end{array}$$

(5e) $$\begin{array}{c} E_{tot}=\sum_{i=1}^{n_{E}}k_{i}x_{E_{i}}^{0}\leq E_{tot,\max}\text{.} \end{array}$$

**Table 4:** Bounds and start values for the metabolite and enzyme concentrations used for the optimization of the product titer of the UDP-GalNAc enzyme cascade.

	Lower bound	Start values	Upper bound	Unit
Uridine	0.1	50	50	mM
GalNAc	0.1	50	50	mM
ATP	0.01	0.5	2.5	mM
GLMU	0.01	0.1	2	g/l
NAHK	0.01	0.1	2	g/l
PPA	0.01	0.03	2	g/l
PPK3	0.01	0.1	1	g/l
UDK	0.01	0.1	2	g/l
UMPK	0.004	0.1	0.8	g/l
$E_{tot,\max}$	-	0.53	0.53	g/l

Solving the optimization problem for each parameter set of the ensemble results in a distribution of optimal initial enzyme and substrate concentrations (Supplementary File S3), which shows that the initial concentrations of the enzymes GLMU, NAHK, and PPK3 are in most cases increased compared to the baseline. As the total enzyme load is fixed, the concentrations of other enzymes need to be reduced to compensate. Here, the initial concentrations of the enzymes UDK, UMPK, and especially PPA are decreased in most cases. The main substrates uridine and GalNAc were limited to their baseline concentrations to exclude the trivial solution of adding more substrate to achieve a higher titer. Regarding the initial concentration of ATP, as expected, the optimizer chose to set it to its upper limit in most solutions, since ATP is the most important phosphate donor during the initial dynamic phase of the process.

All optimization results were cross-validated and scored across all parameter sets of the ensemble to identify the best prediction (Supplementary File S3), which was then tested experimentally (Fig. 8). Comparing the data points of the validation experiment to the average trajectories of the prediction (simulated across the entire parameter ensemble) shows that both agree well, though some errors remain (and some metabolites could not be measured). The simulated trajectories of one of the main substrates, uridine, and notably of the final product, UDP-GalNAc, match the data of the validation experiment especially well.

**Fig. 8:**
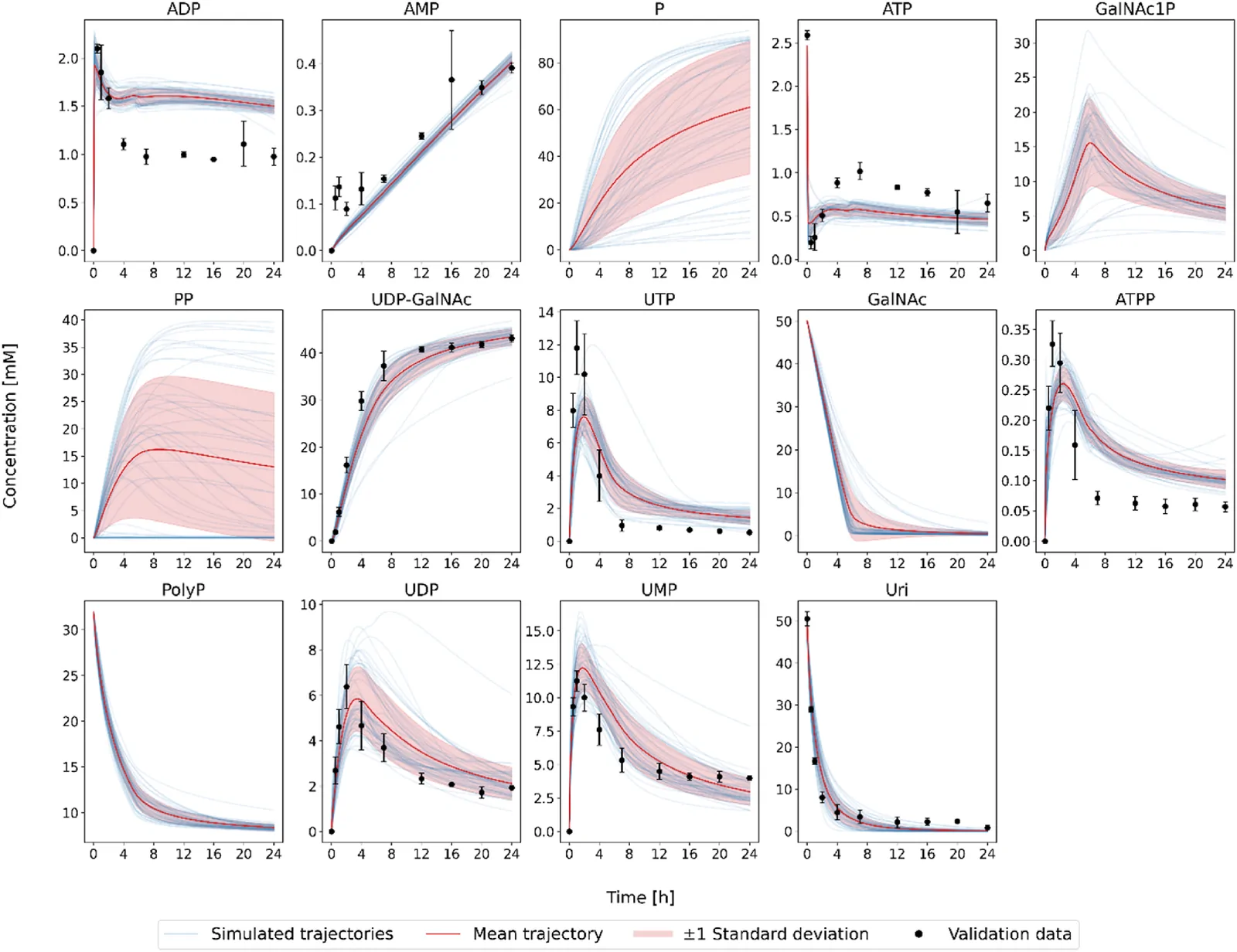
**Simulated trajectories of the predicted optimal initial enzyme and substrate concentrations for titer optimization with data from the experimental validation.** The simulation was repeated for each parameter set of the ensemble (average trajectories ± standard deviation and individual trajectories are shown). Data points from the validation experiment are shown as averages of triplicates.

A comparison between the original and predicted (optimal) initial substrate and enzyme concentrations and the resulting performance of the baseline vs. the optimized process is summarized in Fig. 9. Compared to the baseline, the UDP-GalNAc titer at 7 h could be improved by 44.72 % from 25.76 to 37.28 mmol_UDP-GalNAc_/l and the conversion yield by 44.72 % (from 51.52 to 74.56 % of the maximum conversion yield [mmol_UDP-GalNAc_/mmol_Uridine_ · 100 %]), although the same enzyme load and the same initial concentrations of the main substrates uridine and GalNAc were used in both cases.

**Fig. 9:**
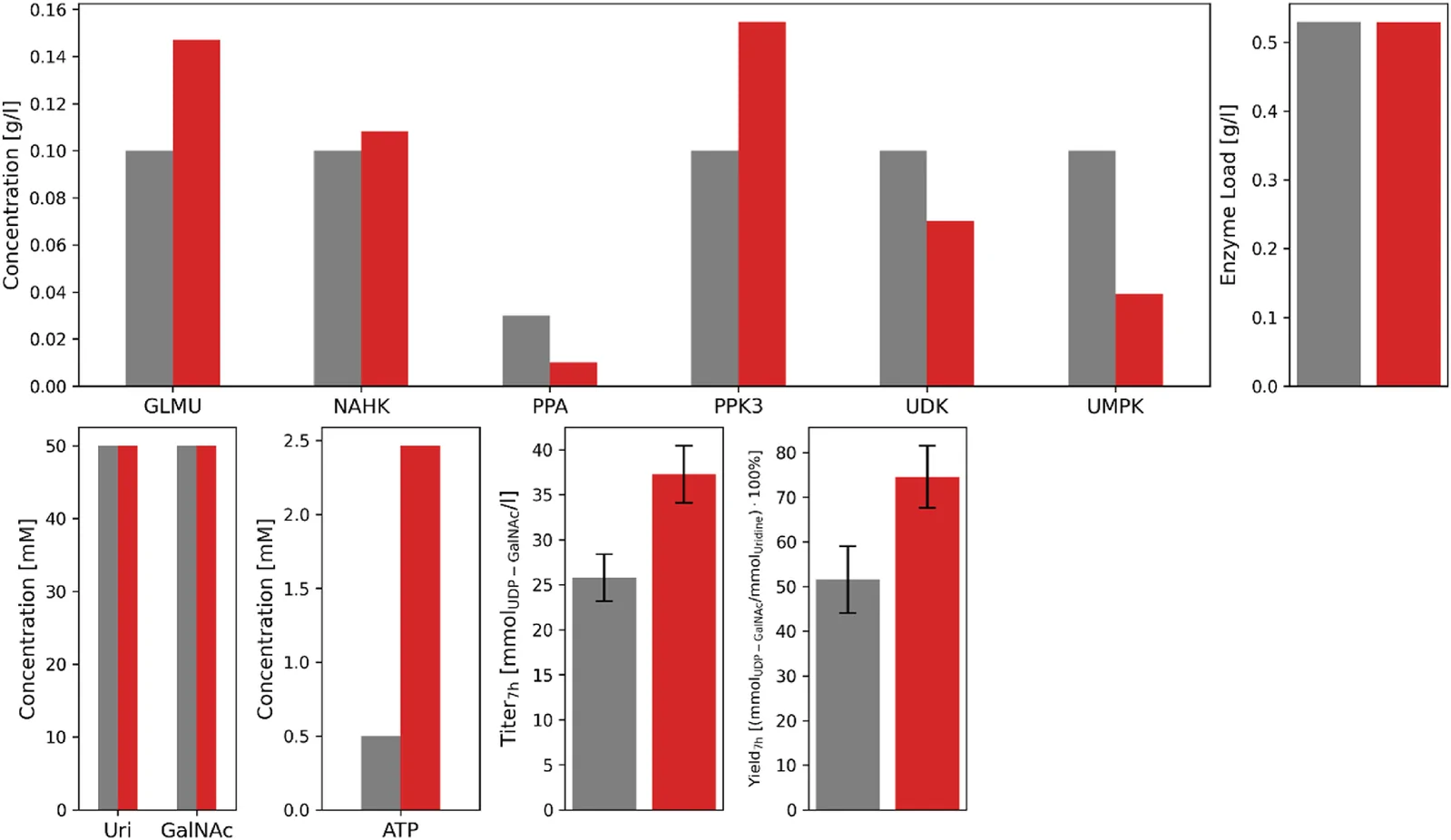
**Initial concentrations and performance of the baseline process (gray) compared to the process optimized for maximum product titer at 7 h (red).** The enzyme load is the sum of all initial enzyme concentrations. The error bars show the standard deviation across three biological replicates.

#### 2.4.2 Optimization of the enzyme load

The second model-based optimization problem (Equations (6a), (6b), (6c), (6d), (6e), (6f)) aims to identify initial enzyme and substrate concentrations that minimize the necessary enzyme load $E_{tot}$ for a given minimum titer and yield of UDP-GalNAc, both measured at $t_{end}=24\;h$ (the yield referring to the substrate uridine). As in the previous section, the kinetic model and the bounds for all optimization variables are included as constraints (Equations (6b), (6c), (6d) respectively; see also Table 5).

(6a) $$\begin{array}{c} \min_{x_{E}^{0},x_{S}^{0}\;}\;E_{tot}=\sum_{i=1}^{n_{E}}x_{E_{i}}^{0} \end{array}$$

s.t.

(6b) $$\begin{array}{c} \dot{x}\left(t\right)=f\left(x\left(t\right),t\right),\forall t\in \left[t_{0},t_{end}\right],x\left(t_{0}\right)=x^{0}\text{,} \end{array}$$

(6c) $$\begin{array}{c} {lb}_{E_{i}}\leq x_{E_{i}}^{0}\leq {ub}_{E_{i}},\forall \;i=1,\ldots ,n_{E}\text{,} \end{array}$$

(6d) $$\begin{array}{c} {lb}_{S_{j}}\leq x_{S_{j}}^{0}{\leq ub}_{S_{j}},\forall \;j=1,\ldots ,n_{S}\text{,} \end{array}$$

(6e) $$\begin{array}{c} x_{UDP-GalNAc}\left(t_{end}\right)\geq x_{UDP-GalNAc,\min}\text{,} \end{array}$$

(6f) $$\begin{array}{c} Y_{P/S\;}=\frac{x_{UDP-GalNAc}\left(t_{end}\right)}{x_{Uridine}^{0}}\geq Y_{P/S,\min}\text{.} \end{array}$$

**Table 5:** **Bounds and start values for the metabolite and enzyme concentrations used for the optimization of the enzyme load.** ATP is not included in the optimization and instead set to a constant value (0.5 mM). The values of the minimum titer and minimum conversion yield constraints are based on the respective values of the baseline process.

	Lower bound	Start value	Upper bound	Unit
Uridine	0.1	50	100	mM
GalNAc	0.1	50	100	mM
GLMU	0.01	0.1	2	g/l
NAHK	0.01	0.1	2	g/l
PPA	0.01	0.03	2	g/l
PPK3	0.01	0.1	1	g/l
UDK	0.01	0.1	2	g/l
UMPK	0.004	0.1	0.8	g/l
$x_{UDP-GalNAc,min}\left(t_{24\;h}\right)$	40.37	-	-	mM
$Y_{P/S,min}$	82.74	-	-	%

Solving the optimization problem for each parameter set of the ensemble results in a distribution of optimal initial enzyme and substrate concentrations (Supplementary File S3), which shows that the concentrations of most enzymes are reduced compared to the baseline. The only exceptions are GLMU and PPK3, where the median concentrations are slightly above the respective baseline levels. Increasing the concentration of these enzymes while trying to achieve an overall lower enzyme load highlights their importance to the performance of the cascade. Additionally, the optimizer slightly reduced the concentrations of the main substrates uridine and GalNAc compared to the baseline.

All optimization results were again cross-validated and scored across all parameter sets of the ensemble to identify the best prediction (Supplementary File S3), which was then tested experimentally (Fig. 10). Comparing the data points of the validation experiment to the average trajectories of the prediction (simulated across the entire parameter ensemble) shows that, despite some mismatches, the trajectories of the main substrate uridine, as well as UMP, and especially the final product UDP-GalNAc, are in good agreement with the validation data.

**Fig. 10:**
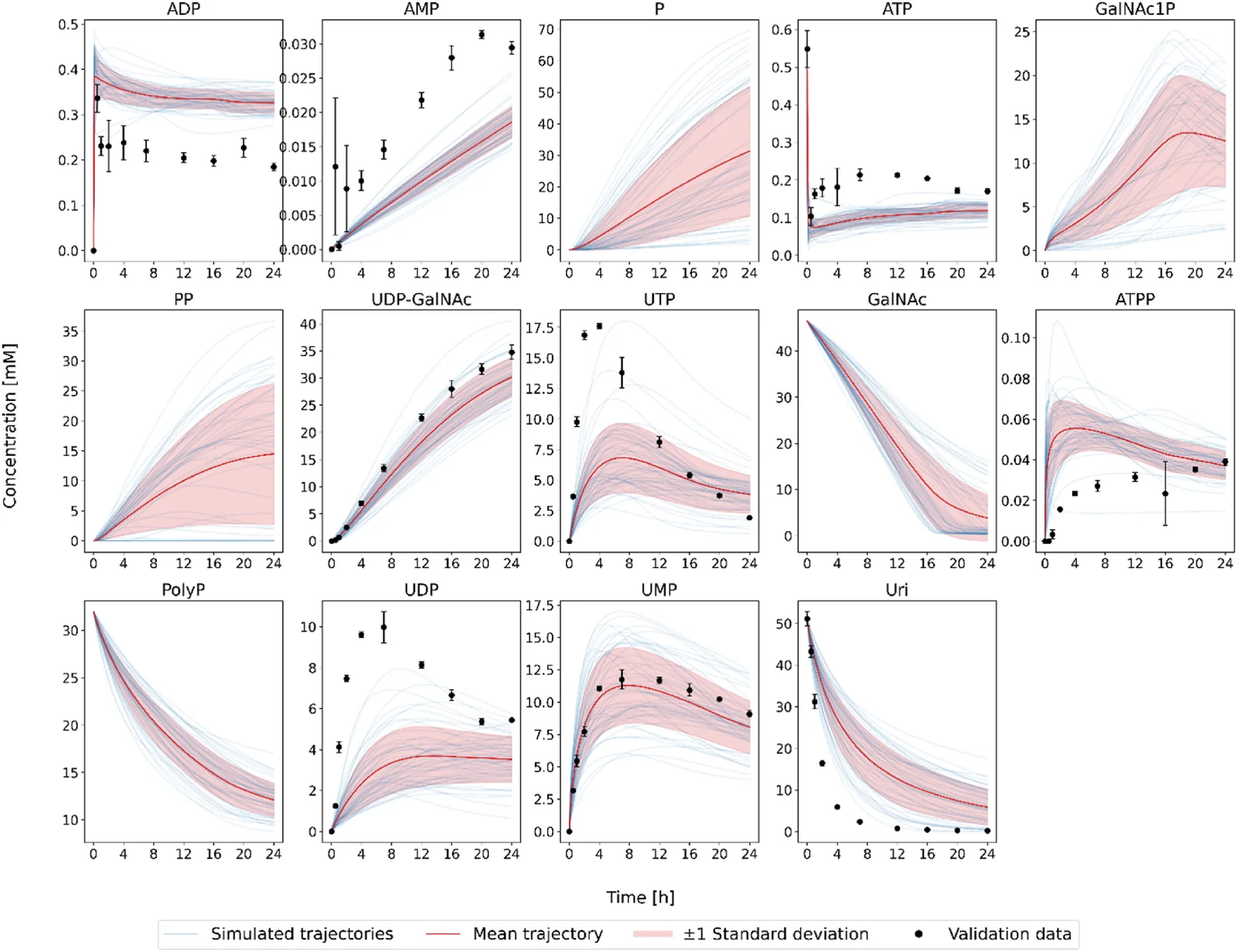
**Simulated trajectories of the predicted optimal initial enzyme and substrate concentrations of the enzyme load optimization with data from the experimental validation.** The simulation was repeated for each parameter set in the ensemble (average trajectories ± standard deviation and individual trajectories are shown). Data points of the validation experiment are shown as averages of triplicates.

A comparison between the original and predicted (optimal) initial enzyme and substrate concentrations and the resulting performance of the baseline and the optimized process is shown in Fig. 11. Compared to the baseline, the enzyme load (and thus related costs) could be significantly reduced by 52.83 % from 0.53 to 0.25 g_enzyme_/l. With the reduced enzyme load, the absolute titer and yield ended up slightly lower (−13.95 % and −12.32 %, respectively) than those of the baseline, thereby violating the minimum titer and yield constraints set in the optimization problem. This can happen because satisfaction of these constraints depends on the selected kinetic parameter set, unlike constraints on initial conditions (e.g. upper bound for the enzyme load). Therefore, when simulations with the other parameter sets of the ensemble are performed during the cross-validation, the titer and yield values can potentially violate the original constraints. For the enzyme load optimization, we found that this applies to 71 % of the titer values and 72 % of the yield values in the respective cross-validation tables. This potential deviation in the cross-validation cannot be taken into account when optimizing the model for a given parameter set (only for the latter the constraints are guaranteed to be fulfilled). Since we are interested in the performance of each optimization result across the entire parameter ensemble, all values (regardless of whether they adhere to the constraints or not) are aggregated and used for the scoring. In this way, the inherent parameter uncertainty is incorporated into the optimization procedure. Although the original values of the absolute titer and yield could not be fully reached, calculating the relative titer and yield (by dividing the absolute titer and yield by the enzyme load) shows that both could be significantly improved. The relative titer was increased by 80.34 % from 76.16 to 137.35 mmol_UDP-GalNAc_/g_enzyme_ and the relative yield was increased by 75.40 % from 152.32 to 267.17 [100 % · mmol_UDP-GalNAc_/(mmol_Uridine_ · g_enzyme_/l)].

**Fig. 11:**
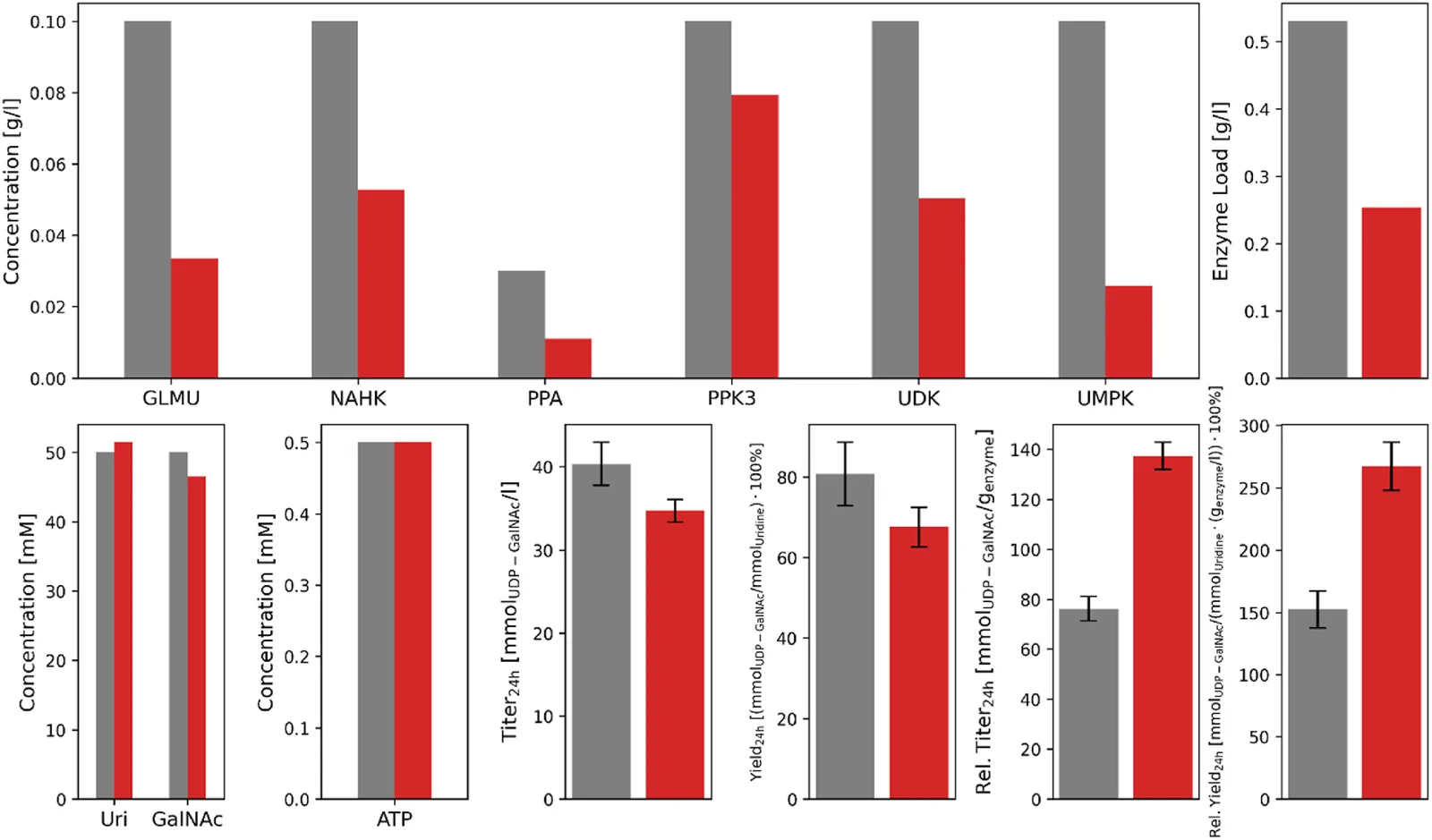
**Initial concentrations and performance of the baseline process (gray) compared to the process optimized for minimum enzyme load (red).** The enzyme load is the sum of all initial enzyme concentrations. Titer and conversion yield refer to the product UDP-GalNAc and the substrate uridine. The relative titer and yield of UDP-GalNAc are calculated by dividing the absolute titer and yield values by the enzyme load. Here, the absolute and relative values show different trends between baseline and optimized performance because the enzyme load is significantly lower in the optimized scenario. For the sake of completeness, the initial ATP concentrations are shown, although ATP was not used as an optimization variable. The error bars show the standard deviation across three biological replicates.

## 3 Discussion

In this work, we presented MoRSel, a new model refinement and selection approach for building kinetic models under structural uncertainties. Starting from an initial core model, a library of possible model changes, and experimental data, the candidate changes are iteratively integrated into the core model, and their respective effects on the model goodness are evaluated by aggregating different model selection criteria. In this way, a curated space of a limited set of initially defined hypotheses is explored using a greedy search algorithm. MoRSel seamlessly integrates with our previously developed framework for robust model-based optimization under parametric uncertainty (Huber et al., 2024), which we applied here to successfully optimize a cell-free enzyme cascade for the production of the nucleotide sugar UDP-GalNAc with respect to product titer and enzyme load.

Different methods have been proposed to solve the problem of selecting the best model from an assortment of different possible variants (see the Introduction). Within this space, MoRSel can be classified as a hypothesis-library-driven refinement of an initial model structure. This increases the biological interpretability of the results and is particularly suited to problems where different structural hypotheses are well defined. Among other available methods, the modelMAGE framework (Flöttmann et al., 2008; Schaber et al., 2011) shares the most similarities with our approach. Their method starts with a master model that contains all possible model components and a set of directives specifying changes to the master model (removing reactions and/or changing their kinetics), which are used to generate model variants. Crucially, the structures of all model variants to be tested need to be explicitly defined by the user and are then subsequently built, evaluated, and ranked by modelMAGE. This requires prior knowledge of which model variants are most promising (from the entirety of models that can be constructed by integrating all combinations of possible mechanistic changes) and makes modelMAGE a method focused exclusively on model selection. By contrast, MoRSel does not require prior definition of specific model variants and instead attempts to explore the entire space of possible model structures emerging from all possible combinations of an initial core model and a given library of model changes. However, the size of this solution space scales exponentially with the number of potential model changes that can be introduced into a given core model: for n model changes, there are $2^{n}$ possible model structures that can be constructed. This makes evaluating all possible model variants infeasible for larger n, due to combinatorial explosion (for example, in our UDP-GalNAc enzyme cascade with 19 candidate model changes, we would have had to test more than 500,000 model variants). For this reason, MoRSel uses a greedy search algorithm, which tests, in the first iteration, the addition of n individual model changes (from which the best resulting model is selected), in the second iteration the addition of n−1 (remaining) individual model changes to the best model from the first iteration, and so forth. In total, this requires the evaluation of only $\frac{n\cdot \left(n+1\right)}{2}$ different model variants. Applying this strategy of iterative refinement of an initial core model in our UDP-GalNAc case, only 190 (instead of 500,000) model variants needed to be tested, from which the overall best model variant could then be selected. In this way, MoRSel combines the advantages of model refinement and model selection approaches. A possible disadvantage of utilizing such a greedy search strategy is that it cannot capture potential synergies between candidate model changes, since model changes are included and tested individually in each iteration of MoRSel's modification loop. While it would be possible to adjust our algorithm to also test (smaller) combinations of candidate changes in each iteration, this would again increase the computational costs in larger networks and may then again become combinatorially intractable. We believe that MoRSel achieves a good balance between search space coverage and computational cost. It is most naturally suited to small-to medium-scale mechanistic kinetic models with a manageable number of plausible structural hypotheses, such as (cell-free) enzyme cascades, pathway modules, or other well-characterized biochemical systems.

The selection of kinetic models is complicated by parameter uncertainty due to practical and structural non-identifiability. Practical non-identifiability, arising from limited or low-quality experimental data, can be mitigated by increasing the amount and/or quality of available data or by subdividing the system into smaller modules. For example, Shen et al. employed a modular approach to first characterize individual enzymes in isolation using initial rate kinetics, which provided a foundation for modeling the full Weimberg pathway cascade (Shen et al., 2020). However, this approach inherently omits interactions between enzymes and metabolites from different reactions, such as substrate channeling or synergistic effects, which can significantly influence the overall cascade performance. As a result, kinetic parameters measured in isolation often require adjustment when applied to the full system. Structural non-identifiability arises due to correlations between parameters, preventing a unique estimation of their values (and thus the goodness of the entire model). This can lead to cases where the same model structure is evaluated differently depending on the underlying parameterization. To handle this kind of uncertainty, any parameter estimation calculated as part of MoRSel is repeated multiple times, and the parameter set with the lowest error is chosen for further computations. Depending on the dimensionality of the parameter space, the number of repetitions can be adjusted, although the same trade-off between higher coverage and higher computational cost also applies here. Furthermore, the opposite problem also needs to be considered: namely, that different model structures can lead to very similar (or even identical) evaluations because combinations of different model changes can compensate for or reinforce each other. Repeating the entire MoRSel *framework* multiple times and choosing the best result across all runs, as practiced herein, can help alleviate this problem since a larger area of the space of possible model structures is explored. Due to the influence of structural and parametric uncertainties as well as stochastic elements of the chosen numerical solvers, it is very unlikely that multiple runs follow exactly the same path through the solution space.

Model-based optimization requires models with sufficient predictive power, which MoRSel can help to identify. We demonstrated this by using the selected kinetic model for the UDP-GalNAc multi-enzyme cascade to successfully generate predictions of optimal initial enzyme and substrate concentrations that led to improvements in the cell-free process, as validated experimentally. While we applied MoRSel to specifically select a kinetic model of a cell-free enzyme cascade, its approach of combining a greedy search strategy and robust ranking across multiple different model selection criteria makes it a general tool that can be applied to other model types and processes.

In this work, all experiments with the UDP-GalNAc cascade were exclusively performed in batch mode with purified enzymes and all necessary substrates and cofactors mixed in a one-pot reaction. Different process designs could be beneficial, such as a fed-batch approach, where additional amounts of substrates and/or cofactors are added over time (Frohnmeyer et al., 2024). This holds potential for increasing the cost efficiency of cell-free processes, as purified enzymes (the most expensive components) can be utilized for longer periods. Furthermore, since reaction rates depend on substrate concentrations, a feeding strategy can maintain high substrate levels to prevent kinetic rate limitation and sustain maximum productivity. However, feeding also results in enzyme and metabolite dilution, which must be accounted for in fed-batch process optimization. The advantages of fed-batch operation are most evident in processes limited by substrate inhibition or where high initial concentrations are impractical due to solubility issues. In addition, continuous operation, where the reaction volume remains constant, would require the net inflow to equal the outflow. In such cases, enzyme immobilization techniques could be employed to prevent enzymes from being washed out from the reactor (Maghraby et al., 2023). Regardless of the configuration, optimal feeding profiles for cell-free fed-batch processes can again be determined using dynamic optimization (Espinel-Ríos et al., 2022). Our presented modeling and optimization framework can be applied in these cases as well, as it only requires identifying the suitable decision variables for the specific process design.

Combining MoRSel and our robust model-based optimization framework to improve the specific cell-free process that we chose proved to be successful. Nonetheless, some parts of the methodology could benefit from further development and analysis. Aspects for future work include analyzing the impact of our custom model selection criteria on the evaluation of model variants, incorporating hybrid machine-learning-supported approaches, and extending our optimization approach to other process designs or optimization problems.

## 4 Material and methods

### 4.1 Recombinant enzyme production

For the cultivation of *E. coli* BL21(DE3) as expression host, a complex growth media (Terrific Broth, Lessard, 2013) was used. Recombinant protein expression was carried out using the standard T7 expression system, with the pET-28a(+) vector for the enzymes UDK, UMPK, PPK3, NAHK, and PPA and the pET-15b vector for the enzyme GLMU. Cultivation took place at 37 °C and 200 rpm until an OD of 0.8-1.2 is reached. Subsequent induction was initiated by 0.4 mM IPTG, followed by incubation at 16 °C and 200 rpm for 20 h. Protein purification was facilitated by immobilized metal affinity chromatography (IMAC), using an Äkta Pure 25 system (GE Healthcare, SE) with a 5 mL HisTrap FF column (Cytiva, DE). The equilibration buffer consisted of 50 mM Tris pH 7.5, 500 mM NaCl, 10 mM MgCl2, 20 mM imidazole and 5 % (v/v) glycerol, while the elution buffer contained 500 mM imidazole. The final enzyme stock solutions were stored in 50 % (v/v) glycerol at −20 °C until use. The purity of the protein samples was determined by SDS-PAGE (Supplementary File S4) using the ImageJ software (National Institutes of Health, USA).

### 4.2 Reaction conditions

The reaction conditions for the multi-enzyme cascade synthesizing UDP-GalNAc were as follows: Synthesis reactions were performed in a total volume of 500 μL in 150 mM Tris (pH 9) containing 120 mM MgCl_2_ and 32 mM PolyP_n_. Unless otherwise stated, the initial enzyme concentrations were 0.1 g/L for UDK, GLMU, UMPK, PPK3, and NAHK, and 0.03 g/L for PPA. Initial concentrations of uridine, GalNAc, and ATP were set to 50 mM, 50 mM, and 0.5 mM, respectively. Enzyme, substrate, and ATP concentrations were adjusted according to the optimization results. The UDP-GalNAc synthesis was initiated by adding the intermediates to the enzyme mixture. The reactions were incubated at 39 °C and 500 rpm for 24 h. For quantification, samples were quenched with 90 °C hot water and diluted with ddH_2_O.

### 4.3 Quantification of UV active components with high performance anion-exchange chromatography (HPAEC)

UDP-GalNAc reaction samples were analyzed by HPAEC-UV (260 nm), using a setup of a CarboPac PA200 analytical and guard column (Thermo Fisher Scientific, USA) and the HPAEC unit ICS-5000+ (Thermo Fisher Scientific, USA). The aqueous system consisted of 100 mM NaOH, ddH_2_O water and 1 M NaOAc. For optimal peak separation, a gradient (Fig. 12) was used at a flow rate of 0.5 ml/min with a column temperature of 30 °C.

**Fig. 12:**
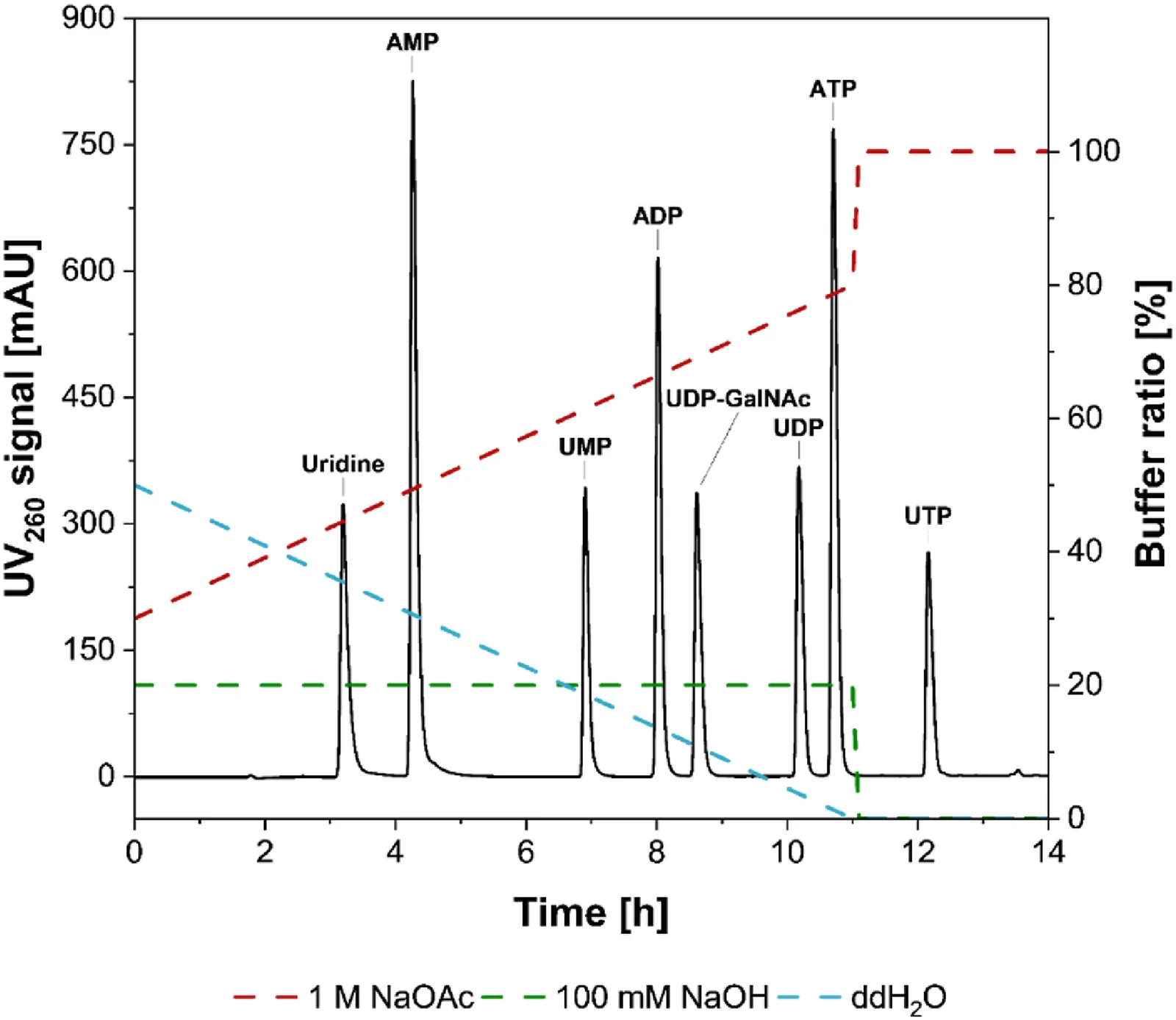
UV signal at 260 nm from the high-performance anion-exchange chromatographic separation of a 70 μM UDP-GalNAc calibration standard solution.

### 4.4 Initial core model of the UDP-GalNAc process

The biochemical network underlying the initial core model is shown in Fig. 5 (all parts drawn in black in this figure are implemented in the initial core model). This includes all known reactions shown in Fig. 2 as well as inhibitions of all enzyme-catalyzed reactions by inorganic phosphate (P). All other parts of Fig. 5 that are not drawn in black are candidate model changes, which are not part of the initial core model. The kinetic rate laws of the initial core model are listed in Table 6.

**Table 6:** Kinetic rate laws of the initial core model.

$r_{GLMU}$	$\begin{array}{c} E_{GLMU}\cdot \frac{k_{cat}^{+}\cdot \frac{GalNAc1P}{K_{m,GalNAc1P}}\cdot \frac{UTP}{K_{m,UTP}}-k_{cat}^{+}\cdot \frac{1}{K_{eq}}\cdot \frac{UDPGalNAc\cdot PP}{K_{m,GalNAc1P}\cdot K_{m,UTP}}}{\left(1+\frac{GalNAc1P}{K_{m,GalNAc1P}}\right)\cdot \left(1+\frac{UTP}{K_{m,UTP}}\right)+\left(1+\frac{UDP_{GalNAc}}{K_{m,UDPGalNAc}}\right)\cdot \left(1+\frac{PP}{K_{m,PP}}\right)-1}\cdot \end{array}\frac{K_{i,P}}{K_{i,P}+P}$

$r_{NAHK}$	$\begin{array}{c} E_{NAHK}\cdot \frac{k_{cat}^{+}\cdot \frac{GalNAc}{K_{m,GalNAc}}\cdot \frac{ATP}{K_{m,ATP}}-k_{cat}^{+}\cdot \frac{1}{K_{eq}}\cdot \frac{GalNAc1P\cdot ADP}{K_{m,GalNAc}\cdot K_{m,ATP}}}{\left(1+\frac{GalNAc}{K_{m,GalNAc}}\right)\cdot \left(1+\frac{ATP}{K_{m,ATP}}\right)+\left(1+\frac{GalNAc1P}{K_{m,GalNAc1P}}\right)\cdot \left(1+\frac{ADP}{K_{m,ADP}}\right)-1}\cdot \end{array}\frac{K_{i,P}}{K_{i,P}+P}$

$r_{PPA}$	$E_{PPA}\cdot k_{cat}^{+}\cdot \frac{1}{1+\frac{K_{m,PP}}{PP}}\cdot \frac{K_{i,P}}{K_{i,P}+P}$

$r_{PPK3_{A}}$	$E_{PPK3}\cdot \;\frac{k_{cat}^{+}\cdot \;\frac{ADP}{K_{m,ADP}\cdot \left(1+\frac{UDP}{K_{m,UDP}}+\frac{UTP}{K_{m,UTP}}\right)}\cdot \;\frac{PolyP}{K_{m,PolyP}}-k_{cat}^{+}\cdot \frac{1}{K_{eq}}\cdot \frac{ATP}{K_{m,ADP}\cdot \left(1+\frac{UDP}{K_{m,UDP}}+\frac{UTP}{K_{m,UTP}}\right)\cdot K_{m,PolyP}}}{\left(1+\frac{ADP}{K_{m,ADP}\cdot \left(1+\frac{UDP}{K_{m,UDP}}+\frac{UTP}{K_{m,UTP}}\right)}\right)\cdot \left(1+\frac{PolyP}{K_{m,PolyP}}\right)+\left(1+\frac{ATP}{K_{m,ATP}\cdot \left(1+\frac{UDP}{K_{m,UDP}}+\frac{UTP}{K_{m,UTP}}\right)}\right)-1}\cdot \frac{K_{i,P}}{K_{i,P}+P}$

$r_{PPK3_{U}}$	$E_{PPK3}\cdot \;\frac{k_{cat}^{+}\cdot \;\frac{UDP}{K_{m,UDP}\cdot \left(1+\frac{ADP}{K_{m,ADP}}+\frac{ATP}{K_{m,ATP}}\right)}\cdot \;\frac{PolyP}{K_{m,PolyP}}-k_{cat}^{+}\cdot \frac{1}{K_{eq}}\cdot \frac{UTP}{K_{m,UDP}\cdot \left(1+\frac{ADP}{K_{m,ADP}}+\frac{ATP}{K_{m,ATP}}\right)\cdot K_{m,PolyP}}}{\left(1+\frac{UDP}{K_{m,UDP}\cdot \left(1+\frac{ADP}{K_{m,ADP}}+\frac{ATP}{K_{m,ATP}}\right)}\right)\cdot \left(1+\frac{PolyP}{K_{m,PolyP}}\right)+\left(1+\frac{UTP}{K_{m,UTP}\cdot \left(1+\frac{ADP}{K_{m,ADP}}+\frac{ATP}{K_{m,ATP}}\right)}\right)-1}\cdot \frac{K_{i,P}}{K_{i,P}+P}$

$r_{UDK}$	$\begin{array}{c} E_{UDK}\cdot \frac{k_{cat}^{+}\cdot \frac{Uri}{K_{m,Uri}}\cdot \frac{ATP}{K_{m,ATP}}-k_{cat}^{+}\cdot \frac{1}{K_{eq}}\cdot \frac{UMP\cdot ADP}{K_{m,Uri}\cdot K_{m,ATP}}}{\left(1+\frac{Uri}{K_{m,Uri}}\right)\cdot \left(1+\frac{ATP}{K_{m,ATP}}\right)+\left(1+\frac{UMP}{K_{m,UMP}}\right)\cdot \left(1+\frac{ADP}{K_{m,ADP}}\right)-1}\cdot \frac{K_{i,P}}{K_{i,P}+P} \end{array}$

$r_{UMPK}$	$\begin{array}{c} E_{UMPK}\cdot \frac{k_{cat}^{+}\cdot \frac{UMP}{K_{m,UMP}}\cdot \frac{ATP}{K_{m,ATP}}-k_{cat}^{+}\cdot \frac{1}{K_{eq}}\cdot \frac{UDP\cdot ADP}{K_{m,UMP}\cdot K_{m,ATP}}}{\left(1+\frac{UMP}{K_{m,UMP}}\right)\cdot \left(1+\frac{ATP}{K_{m,ATP}}\right)+\left(1+\frac{UDP}{K_{m,UDP}}\right)\cdot \left(1+\frac{ADP}{K_{m,ADP}}\right)-1}\cdot \frac{K_{i,P}}{K_{i,P}+P} \end{array}$

The ordinary differential equations (ODEs) of the initial core model are assembled from the rate laws in Table 6 according to the stoichiometry of the network (Fig. 5). Since enzymes are neither consumed nor produced (and because a significant loss of enzyme activity could not be observed for the relevant time period of 24 h), their ODEs were set to 0.

$$d\left[ADP\right]dt=+r_{NAHK}-r_{PPK3_{A}}+r_{UDK}+r_{UMPK}$$

$$d\left[ATP\right]dt=-{\;r}_{NAHK}+r_{PPK3_{A}}-r_{UDK}-r_{UMPK}$$

$$d\left[GalNAc\right]dt=-{\;r}_{NAHK}$$

$$d\left[GalNAc1P\right]dt=-r_{GLMU}+r_{NAHK}$$

$$d\left[P\right]dt=+2\cdot r_{PPA}$$

$$d\left[PolyP\right]dt=-r_{PPK3_{A}}-r_{PPK3_{U}}$$

$$d\left[PP\right]dt=+r_{GLMU}-r_{PPA}$$

$$d\left[UDP\right]dt=-{\;r}_{PPK3_{U}}+r_{UMPK}$$

$$d\left[UDP\_GalNAc\right]dt=+r_{GLMU}$$

$$d\left[UMP\right]dt=+{\;r}_{UDK}-r_{UMPK}$$

$$d\left[Uri\right]dt=-{\;r}_{UDK}$$

$$d\left[UTP\right]dt=-r_{GLMU}+r_{PPK3_{U}}$$

### 4.5 Parameter estimation

Kinetic model parameters were estimated using data from 12 experiments (Supplementary File S1). The start values (initial guesses) of some parameters for the estimations were obtained from the literature, and the upper and lower bounds were also derived from these literature values. Equilibrium constants were sourced from eQuilibrator with pH set to 9 and pMg set to 0.9. The start values and bounds of the remaining parameters were set to different default values, depending on their type. The start values for inhibition and activation constants were chosen such that the associated regulation terms are initialized in a deactivated state (meaning no inhibitory or activating effect on the reaction rate) at the beginning of each estimation. All parameter ensembles throughout this work were generated by repeating parameter estimations with the same setup according to Table 7. The estimations were calculated using the Particle Swarm algorithm implemented in COPASI (iteration limit: 800, swarm size: 50, standard deviation: 1e-06).

**Table 7:** Setup for all parameter estimations.

	Parameter Name	Start Value	Start Value Reference	Lower Bound	Upper Bound	Unit
Start values taken from literature	(GLMU). $K_{eq}$	210	eQuilibrator (Beber et al., 2022)	50 % of start value	150 % of start value	-
	(NAHK). $k_{cat}^{+}$	2707.2	Mamoru and Motomitsu (2007)			1/h
	(NAHK). $K_{m,GalNAc}$	0.065				mM
	(NAHK). $K_{m,ATP}$	0.172				mM
	(NAHK). $K_{eq}$	60	eQuilibrator (Beber et al., 2022)			-
	(UDK_ATP). $K_{eq}$	12000				-
	(UMPK_ATP). $K_{eq}$	5				-
Start values used for unknown parameter values	$k_{cat}^{+}$ Catalytic constants	3.6e+04	-	3.6e+02	3.6e+06	1/h
	$K_{eq}$ Equilibrium constants	1		1e-03	1e+03	-
	$K_{m}$ Michaelis-Menten constants	1e-01		1e-02	1e+02	mM
	$K_{i}$ Inhibition constants	1e+04		1e-02	1e+04	mM
	$K_{a}$ Reciprocal activation constants	0		0	1e+03	1/mM
	$K_{MA}$ Mass action constants	1		1e-06	1e+06	1/h

### 4.6 Selected model of the UDP-GalNAc process

A network of the selected model variant is shown in Fig. 6 (black: parts of the initial core model, blue: selected stoichiometric model changes, red/green: selected kinetic model changes). Table 8 lists the kinetic rate laws of the selected model variant. Bold text highlights the differences compared to the rate laws of the initial core model, which are the result of integrating the selected kinetic model changes.

**Table 8:** Kinetic rate laws of the selected model variant.

$r_{ADPDecayV2}$	$K_{MA}\cdot ADP^{2}$

$r_{GLMU}$	$E_{GLMU}\cdot \frac{k_{cat}^{+}\cdot \frac{GalNAc1P}{K_{m,GalNAc1P}}\cdot \frac{UTP}{K_{m,UTP}}-k_{cat}^{+}\cdot \frac{1}{K_{eq}}\cdot \frac{UDPGalNAc\cdot PP}{K_{m,GalNAc1P}\cdot K_{m,UTP}}}{\left(1+\frac{GalNAc1P}{K_{m,GalNAc1P}}\right)\cdot \left(1+\frac{UTP}{K_{m,UTP}}\right)+\left(1+\frac{UDP_{GalNAc}}{K_{m,UDPGalNAc}}\right)\cdot \left(1+\frac{PP}{K_{m,PP}}\right)-1}\cdot \frac{K_{i,P}}{K_{i,P}+P}\cdot \frac{K_{i,ADP}}{K_{i,ADP}+ADP}$

$r_{NAHK}$	$\begin{array}{c} E_{NAHK}\cdot \frac{k_{cat}^{+}\cdot \frac{GalNAc}{K_{m,GalNAc}}\cdot \frac{ATP}{K_{m,ATP}}-k_{cat}^{+}\cdot \frac{1}{K_{eq}}\cdot \frac{GalNAc1P\cdot ADP}{K_{m,GalNAc}\cdot K_{m,ATP}}}{\left(1+\frac{GalNAc}{K_{m,GalNAc}}\right)\cdot \left(1+\frac{ATP}{K_{m,ATP}}\right)+\left(1+\frac{GalNAc1P}{K_{m,GalNAc1P}}\right)\cdot \left(1+\frac{ADP}{K_{m,ADP}}\right)-1}\cdot \end{array}\frac{K_{i,P}}{K_{i,P}+P}$

$r_{PPA}$	$E_{PPA}\cdot k_{cat}^{+}\cdot \frac{1}{1+\frac{K_{m,PP}}{PP}}\cdot \frac{K_{i,P}}{K_{i,P}+P}\cdot \left(1+\left(K_{a,ATP}\cdot ATP\right)\right)$

$r_{PPK3_{A}}$	$E_{PPK3}\cdot \;\frac{k_{cat}^{+}\cdot \;\frac{ADP}{K_{m,ADP}\cdot \left(1+\frac{UDP}{K_{m,UDP}}+\frac{UTP}{K_{m,UTP}}+\frac{ATP}{K_{m,ATP}}+\frac{ATPP}{K_{m,ATPP}}\right)}\cdot \;\frac{PolyP}{K_{m,PolyP}}-k_{cat}^{+}\cdot \frac{1}{K_{eq}}\cdot \frac{ATP}{K_{m,ADP}\cdot \left(1+\frac{UDP}{K_{m,UDP}}+\frac{UTP}{K_{m,UTP}}+\frac{ATP}{K_{m,ATP}}+\frac{ATPP}{K_{m,ATPP}}\right)\cdot K_{m,PolyP}}}{\left(1+\frac{ADP}{K_{m,ADP}\cdot \left(1+\frac{UDP}{K_{m,UDP}}+\frac{UTP}{K_{m,UTP}}+\frac{ATP}{K_{m,ATP}}+\frac{ATPP}{K_{m,ATPP}}\right)}\right)\cdot \left(1+\frac{PolyP}{K_{m,PolyP}}\right)+\left(1+\frac{ATP}{K_{m,ATP}\cdot \left(1+\frac{UDP}{K_{m,UDP}}+\frac{UTP}{K_{m,UTP}}+\frac{ATP}{K_{m,ATP}}+\frac{ATPP}{K_{m,ATPP}}\right)}\right)-1}\cdot \frac{K_{i,P}}{K_{i,P}+P}$

$r_{PPK3_{ATP}}$	$E_{PPK3}\cdot \frac{k_{cat}^{+}\cdot \frac{ATP}{K_{m,ATP}\cdot \left(1+\frac{ADP}{K_{m,ADP}}+\frac{UDP}{K_{m,UDP}}+\frac{ATP}{K_{m,ATP}}+\frac{UTP}{K_{m,UTP}}\right)}\cdot \frac{PolyP}{K_{m,PolyP}}-k_{cat}^{+}\cdot \frac{1}{K_{eq}}\cdot \frac{ATPP}{K_{m,\;ATP}\cdot \left(1+\frac{ADP}{K_{m,ADP}}+\frac{UDP}{K_{m,UDP}}+\frac{ATP}{K_{m,ATP}}+\frac{UTP}{K_{m,UTP}}\right)\cdot K_{m,PolyP}}}{\left(1+\frac{ATP}{K_{m,ATP}\cdot \left(1+\frac{ADP}{K_{m,ADP}}+\frac{UDP}{K_{m,UDP}}+\frac{ATP}{K_{m,ATP}}+\frac{UTP}{K_{m,UTP}}\right)}\right)\cdot \dot{\left(1+\frac{PolyP}{K_{m,PolyP}}\right)}+\left(1+\frac{ATPP}{K_{m,ATPP}\cdot \left(1+\frac{ADP}{K_{m,ADP}}+\frac{UDP}{K_{m,UDP}}+\frac{ATP}{K_{m,ATP}}+\frac{UTP}{K_{m,UTP}}\right)\;}\right)-1}\cdot \frac{K_{i,P}}{K_{i,P}+P}$

$r_{PPK3_{U}}$	$E_{PPK3}\cdot \;\frac{k_{cat}^{+}\cdot \;\frac{UDP}{K_{m,UDP}\cdot \left(1+\frac{ADP}{K_{m,ADP}}+2\cdot \frac{ATP}{K_{m,ATP}}+\frac{ATPP}{K_{m,ATPP}}\right)}\cdot \;\frac{PolyP}{K_{m,PolyP}}-k_{cat}^{+}\cdot \frac{1}{K_{eq}}\cdot \frac{UTP}{K_{m,UDP}\cdot \left(1+\frac{ADP}{K_{m,ADP}}+2\cdot \frac{ATP}{K_{m,ATP}}+\frac{ATPP}{K_{m,ATPP}}\right)\cdot K_{m,PolyP}}}{\left(1+\frac{UDP}{K_{m,UDP}\cdot \left(1+\frac{ADP}{K_{m,ADP}}+2\cdot \frac{ATP}{K_{m,ATP}}+\frac{ATPP}{K_{m,ATPP}}\right)}\right)\cdot \left(1+\frac{PolyP}{K_{m,PolyP}}\right)+\left(1+\frac{UTP}{K_{m,UTP}\cdot \left(1+\frac{ADP}{K_{m,ADP}}+2\cdot \frac{ATP}{K_{m,ATP}}+\frac{ATPP}{K_{m,ATPP}}\right)}\right)-1}\cdot \frac{K_{i,P}}{K_{i,P}+P}$

$r_{UDK}$	$\begin{array}{c} E_{UDK}\cdot \frac{k_{cat}^{+}\cdot \frac{Uri}{K_{m,Uri}}\cdot \frac{ATP}{K_{m,ATP}}-k_{cat}^{+}\cdot \frac{1}{K_{eq}}\cdot \frac{UMP\cdot ADP}{K_{m,Uri}\cdot K_{m,ATP}}}{\left(1+\frac{Uri}{K_{m,Uri}}\right)\cdot \left(1+\frac{ATP}{K_{m,ATP}}\right)+\left(1+\frac{UMP}{K_{m,UMP}}\right)\cdot \left(1+\frac{ADP}{K_{m,ADP}}\right)-1}\cdot \frac{K_{i,P}}{K_{i,P}+P}\cdot \frac{K_{i,UTP}}{K_{i,UTP}+UTP} \end{array}$

$r_{UMPK}$	$\begin{array}{c} E_{UMPK}\cdot \frac{k_{cat}^{+}\cdot \frac{UMP}{K_{m,UMP}}\cdot \frac{ATP}{K_{m,ATP}}-k_{cat}^{+}\cdot \frac{1}{K_{eq}}\cdot \frac{UDP\cdot ADP}{K_{m,UMP}\cdot K_{m,ATP}}}{\left(1+\frac{UMP}{K_{m,UMP}}\right)\cdot \left(1+\frac{ATP}{K_{m,ATP}}\right)+\left(1+\frac{UDP}{K_{m,UDP}}\right)\cdot \left(1+\frac{ADP}{K_{m,ADP}}\right)-1}\cdot \frac{K_{i,P}}{K_{i,P}+P} \end{array}$

The ODEs are assembled from the kinetic rate laws according to the stoichiometry of the selected model network (Fig. 6). As enzymes are neither consumed nor produced, their rates of change over time are 0. Bold text highlights the differences compared to the ODEs of the initial core model, which are the result of integrating the selected stoichiometric model changes.

$$d\left[ADP\right]dt=-2\cdot r_{ADPDecayV2}+r_{NAHK}-r_{PPK3_{A}}+r_{UDK}+r_{UMPK}$$

$$d\left[AMP\right]dt=+r_{ADPDecayV2}$$

$$d\left[ATP\right]dt=+r_{ADPDecayV2}-r_{NAHK}+r_{PPK3_{A}}-r_{PPK3_{ATP}}-r_{UDK}-r_{UMPK}$$

$$d\left[ATPP\right]dt=+r_{PPK3_{ATP}}$$

d[E_GLMU]dt=0

d[E_NAHK]dt=0

d[E_PPA]dt=0

d[E_PPK3]dt=0

d[E_UDK]dt=0

d[E_UMPK]dt=0

$$d\left[GalNAc\right]dt=-r_{NAHK}$$

$$d\left[GalNAc1P\right]dt=-r_{GLMU}+r_{NAHK}$$

$$d\left[P\right]dt=+2\cdot r_{PPA}$$

$$d\left[PolyP\right]dt=-r_{PPK3_{A}}-r_{PPK3_{U}}-r_{PPK3_{ATP}}$$

$$d\left[PP\right]dt=+r_{GLMU}-r_{PPA}$$

$$d\left[UDP\right]dt=-r_{PPK3_{U}}+r_{UMPK}$$

$$d\left[UDP\_GalNAc\right]dt=+r_{GLMU}$$

$$d\left[UMP\right]dt=+r_{UDK}-r_{UMPK}$$

$$d\left[Uri\right]dt=-r_{UDK}$$

$$d\left[UTP\right]dt=-r_{GLMU}+r_{PPK3_{U}}$$

## CRediT authorship contribution statement

**Nicolas Huber:** Writing – review & editing, Writing – original draft, Visualization, Software, Methodology, Investigation, Formal analysis, Conceptualization. **Tuan Son Hoang:** Writing – review & editing, Visualization, Validation, Investigation, Data curation. **Sebastián Espinel Ríos:** Writing – review & editing, Methodology, Formal analysis. **Thomas F.T. Rexer:** Writing – review & editing, Resources, Project administration. **Udo Reichl:** Writing – original draft, Supervision, Resources, Funding acquisition. **Steffen Klamt:** Writing – review & editing, Writing – original draft, Supervision, Project administration, Methodology, Formal analysis, Conceptualization.

## Declaration of competing interest

Thomas Rexer reports financial support was provided by eversyn GmbH. Thomas Rexer reports a relationship with eversyn GmbH that includes: board membership, employment, and equity or stocks. The other authors declare that they have no known competing financial interests or personal relationships that could have appeared to influence the work reported in this paper.

## Data Availability

All scripts, data and models used herein are publicly available via GitHub (https://github.com/klamt-lab/MoRSel_UDP-GalNAc_Paper) and archived on Zenodo (https://doi.org/10.5281/zenodo.19098183).
