# Supplementary material for: Combining automated kinetic model selection with robust optimization and application to maximize cell-free production of UDP-GalNAc
Source: Metab Eng Commun. 2026 Sep 8;23:e00287. doi: 10.1016/j.mec.2026.e00287 (PMC13634411; doi:10.1016/j.mec.2026.e00287)

Supplementary File S1

Combining Automated Kinetic Model Selection with Robust Optimization and Application to Maximize Cell-free Production of UDP-GalNAc

Nicolas Huber[1,#], Tuan Son Hoang[1,2, #], Sebastián Espinel Ríos[3], Thomas F.T. Rexer[1,4], Udo Reichl[1,2], Steffen Klamt[1, *]

[1] Max Planck Institute for Dynamics of Complex Technical Systems, 39106 Magdeburg, Germany

[2] Otto von Guericke University Magdeburg, 39106 Magdeburg, Germany

[3] School of Chemical and Bioprocess Engineering, University College Dublin, D04 V1W8, Dublin, Ireland

[4] eversyn, 39106 Magdeburg, Germany

*Corresponding author: klamt@mpi-magdeburg.mpg.de

#Equal contribution

Supplementary File S1: Experimental time course data of the UDP-GalNAc cascade

Table A: Overview of the experimental data. 14 experiments were performed with different initial substrate and enzyme concentrations. The concentrations mentioned in the configurations 36 (experiments 1-4) and 37 (experiments 5-8) are the initial concentrations set for the main substrates: uridine and GalNAc (5, 10, 20, or 50 mM). For configuration 36 all initial enzyme concentrations were set to 1 g/l except PPA, which was set to 0.3 g/l. For configuration 37, different distributions of initial enzyme concentrations (A and B) were set. The initial concentration of ATP was set to 0.5 mM in both configurations 36 and 37. The predictions of the optimizations presented in this work were tested in experiments 13 and 14. L: enzyme load optimization, T: titer optimization (at 7 h), O: selected (extended) model.

Experiment	Configuration Data Set	Usage
1	UDP-GalNAc Conf. 36 (5 mM)	Training Data (Parameter Estimations as part of the Model Selection)
2	UDP-GalNAc Conf. 36 (10 mM)	
3	UDP-GalNAc Conf. 36 (20 mM)	
4	UDP-GalNAc Conf. 36 (50 mM)	
5	UDP-GalNAc Conf. 37 (20 mM, A)	
6	UDP-GalNAc Conf. 37 (50 mM, A)	
7	UDP-GalNAc Conf. 37 (20 mM, B)	
8	UDP-GalNAc Conf. 37 (50 mM, B)	
9	UDP-GalNAc Conf. 39 (V1)	
10	UDP-GalNAc Conf. 39 (V2)	
11	UDP-GalNAc Conf. 39 (V3)	
12	UDP-GalNAc Conf. 39 (V4)	
13	UDP-GalNAc Conf. 40 (TO, 7h)	Validation Exp. (Titer Optimization)
14	UDP-GalNAc Conf. 40 (LO)	Validation Exp. (Enzyme Load Optimization)

	Initial Enzyme Concentrations [g/l]						Initial Substrate Concentrations [mM]		
#	GLMU	NAHK	PPA	PPK3	UDK	UMPK	GalNAc	Uridine	ATP
1	0.1	0.1	0.03	0.1	0.1	0.1	5	5	0.5
2	0.1	0.1	0.03	0.1	0.1	0.1	10	10	0.5
3	0.1	0.1	0.03	0.1	0.1	0.1	20	20	0.5
4	0.1	0.1	0.03	0.1	0.1	0.1	50	50	0.5
5	0.080	0.120	0.036	0.1	0.1	0.080	20	20	0.5
6	0.080	0.120	0.036	0.1	0.1	0.080	50	50	0.5
7	0.120	0.080	0.030	0.080	0.12	0.1	20	20	0.5
8	0.120	0.080	0.030	0.080	0.12	0.1	50	50	0.5
9	0.117	0.035	0.051	0.248	0.037	0.039	50	50	2.5
10	0.097	0.061	0.014	0.259	0.045	0.052	50	50	2.5
11	0.046	0.021	0.011	0.072	0.013	0.011	47.220	48.866	2.5
12	0.035	0.025	0.010	0.088	0.019	0.011	49.509	48.662	2.5
13	0.147	0.108	0.010	0.155	0.070	0.039	50	50	2.47
14	0.034	0.053	0.011	0.079	0.050	0.026	46.486	51.410	0.5

Experiment 1: UDP-GalNAc Conf. 36 (5 mM)

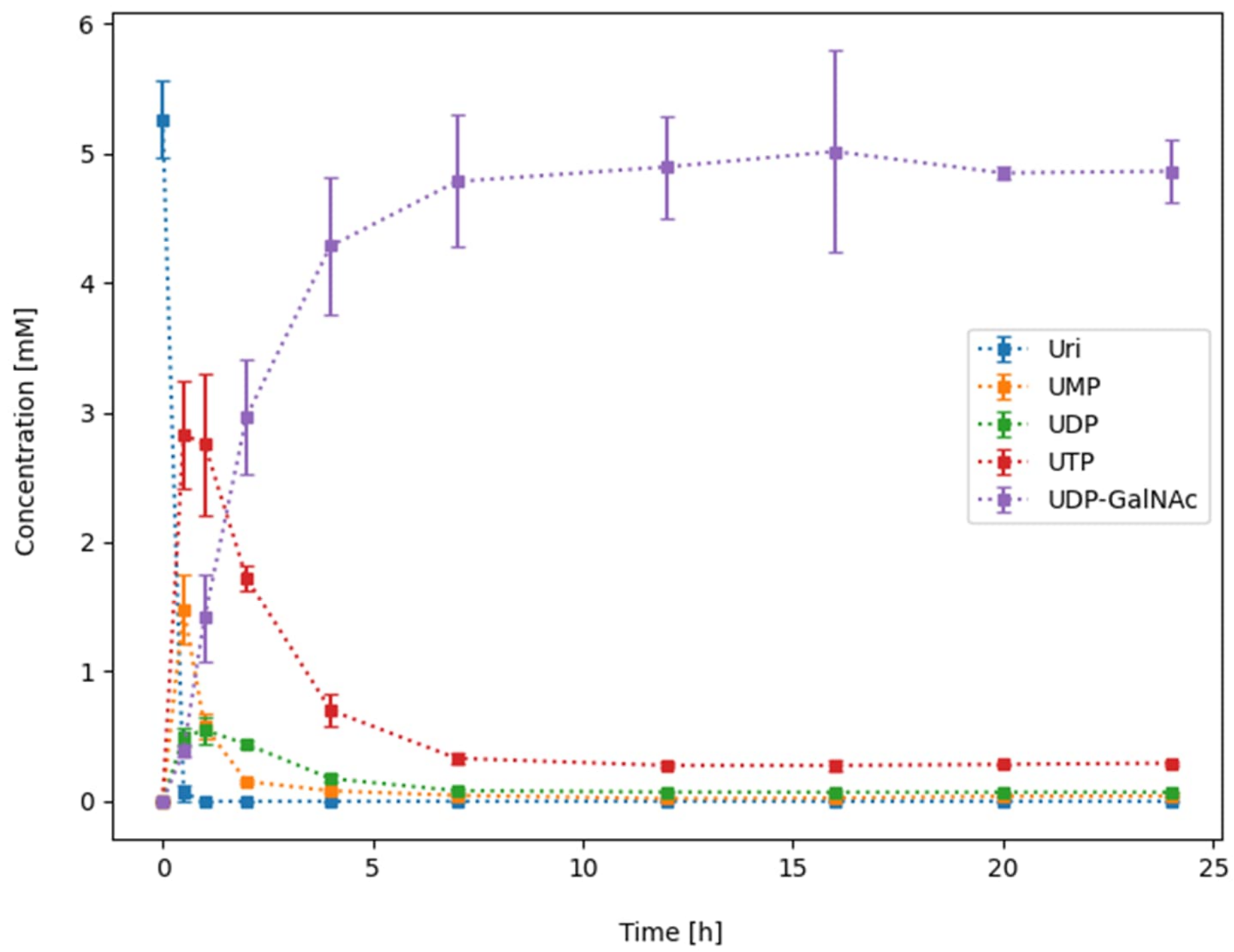

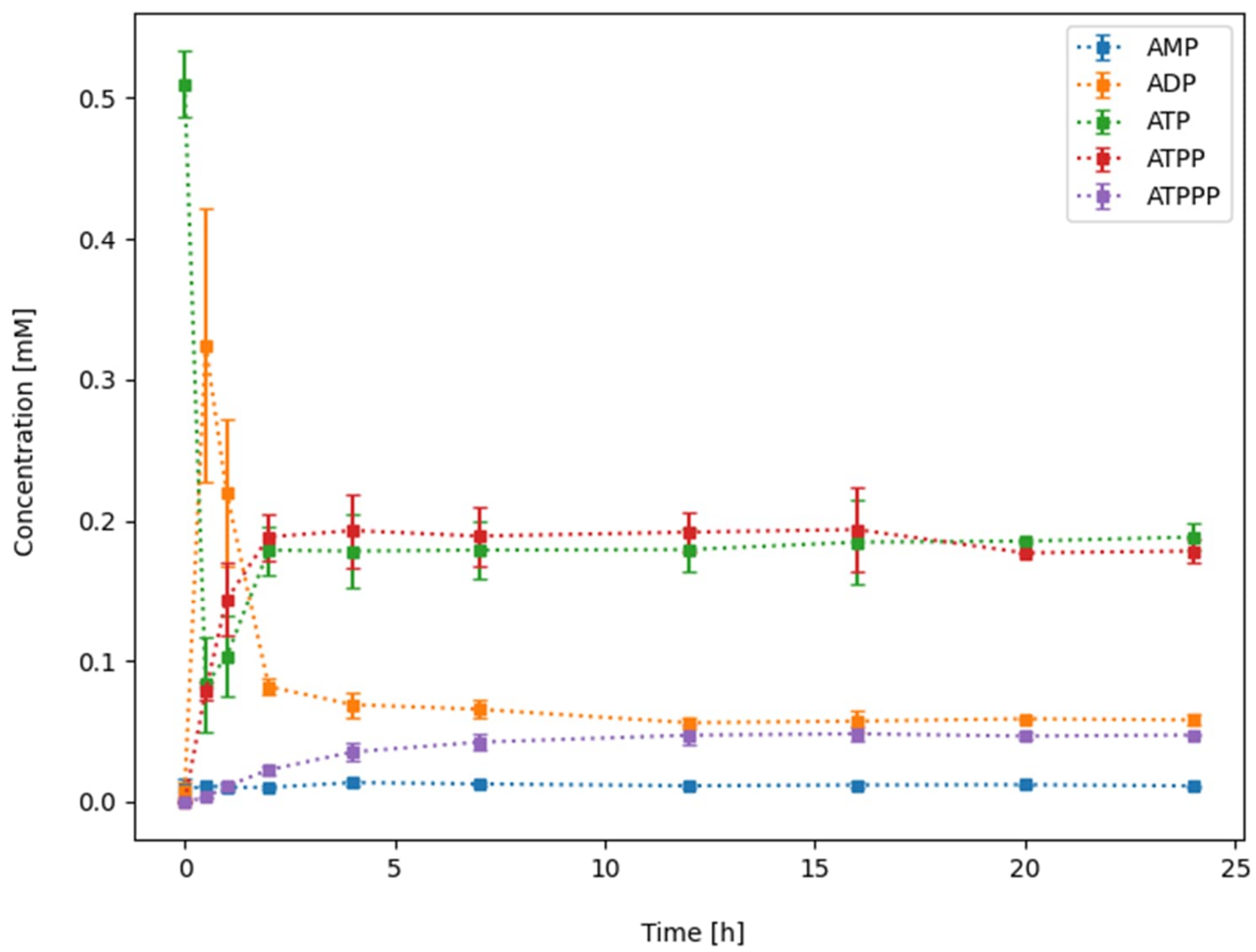

Experiment 2: UDP-GalNAc Conf. 36 (10 mM)

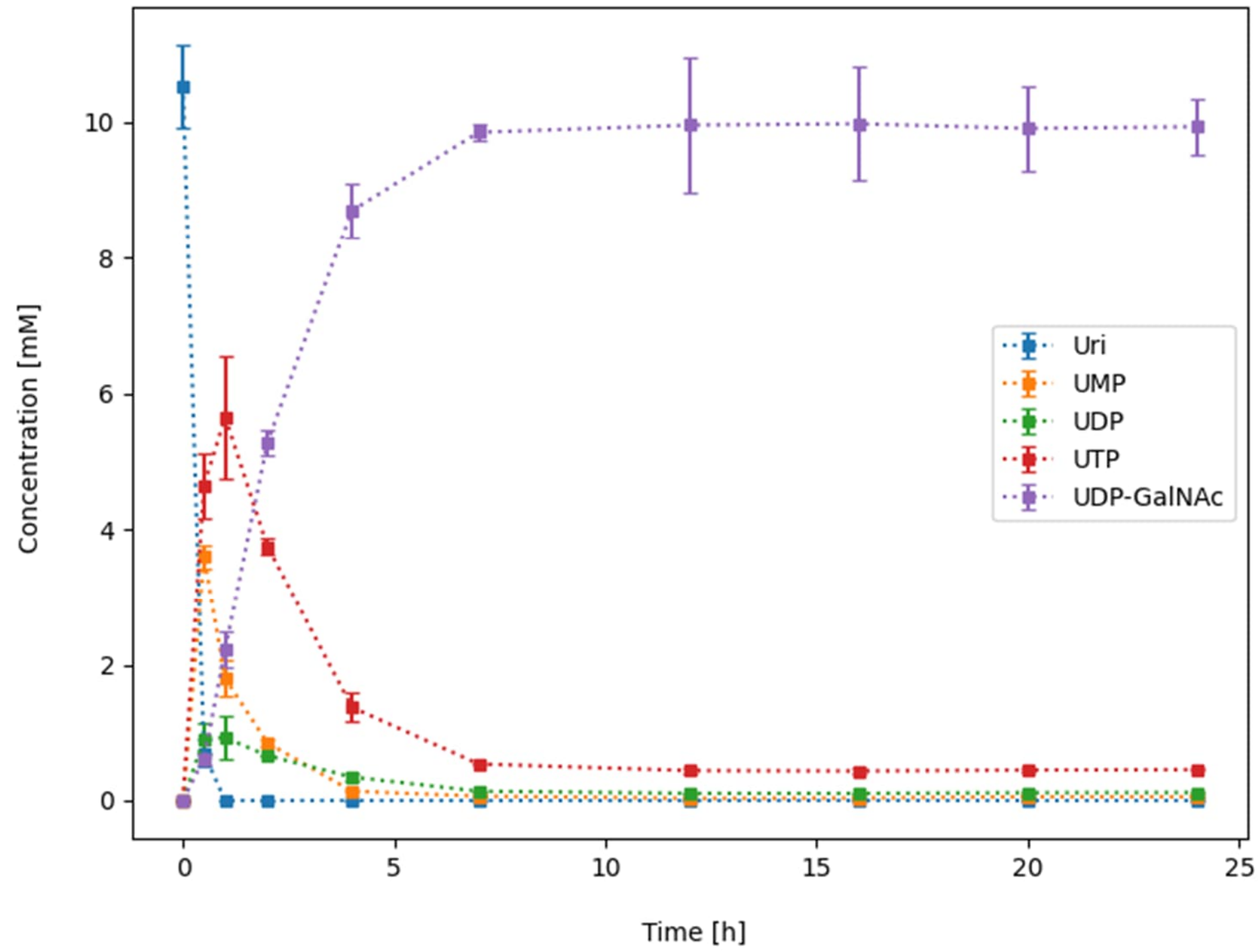

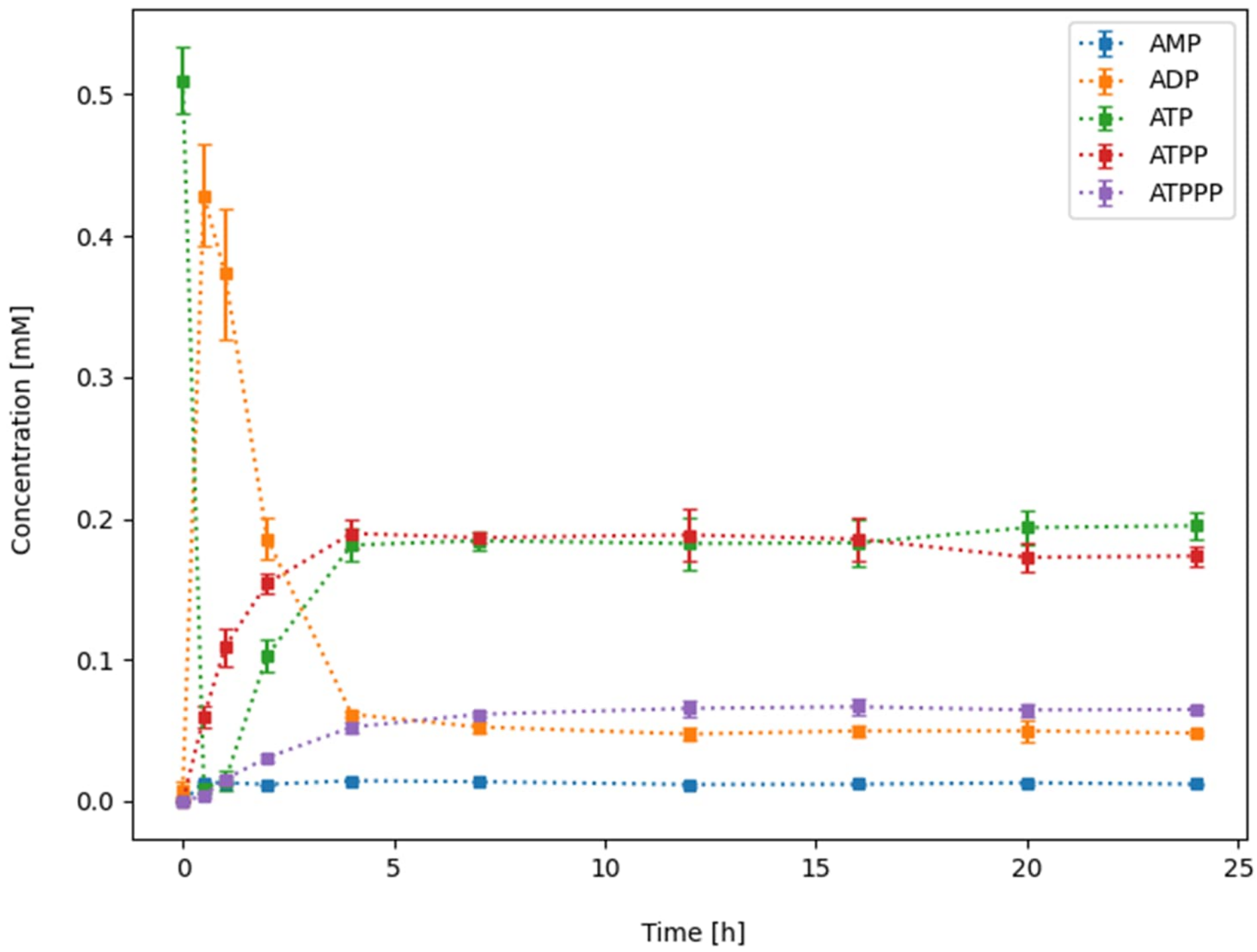

Experiment 3: UDP-GalNAc Conf. 36 (20 mM)

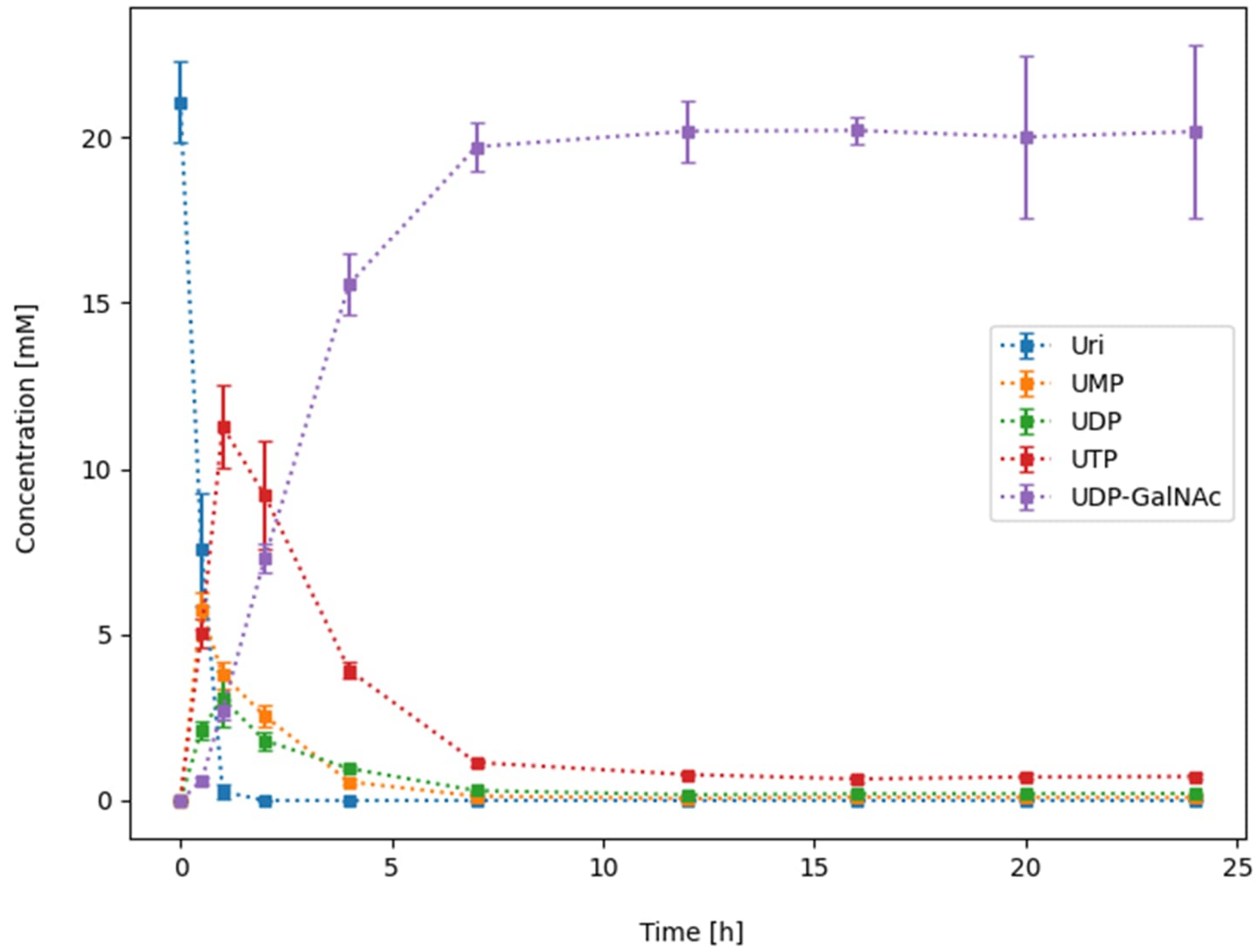

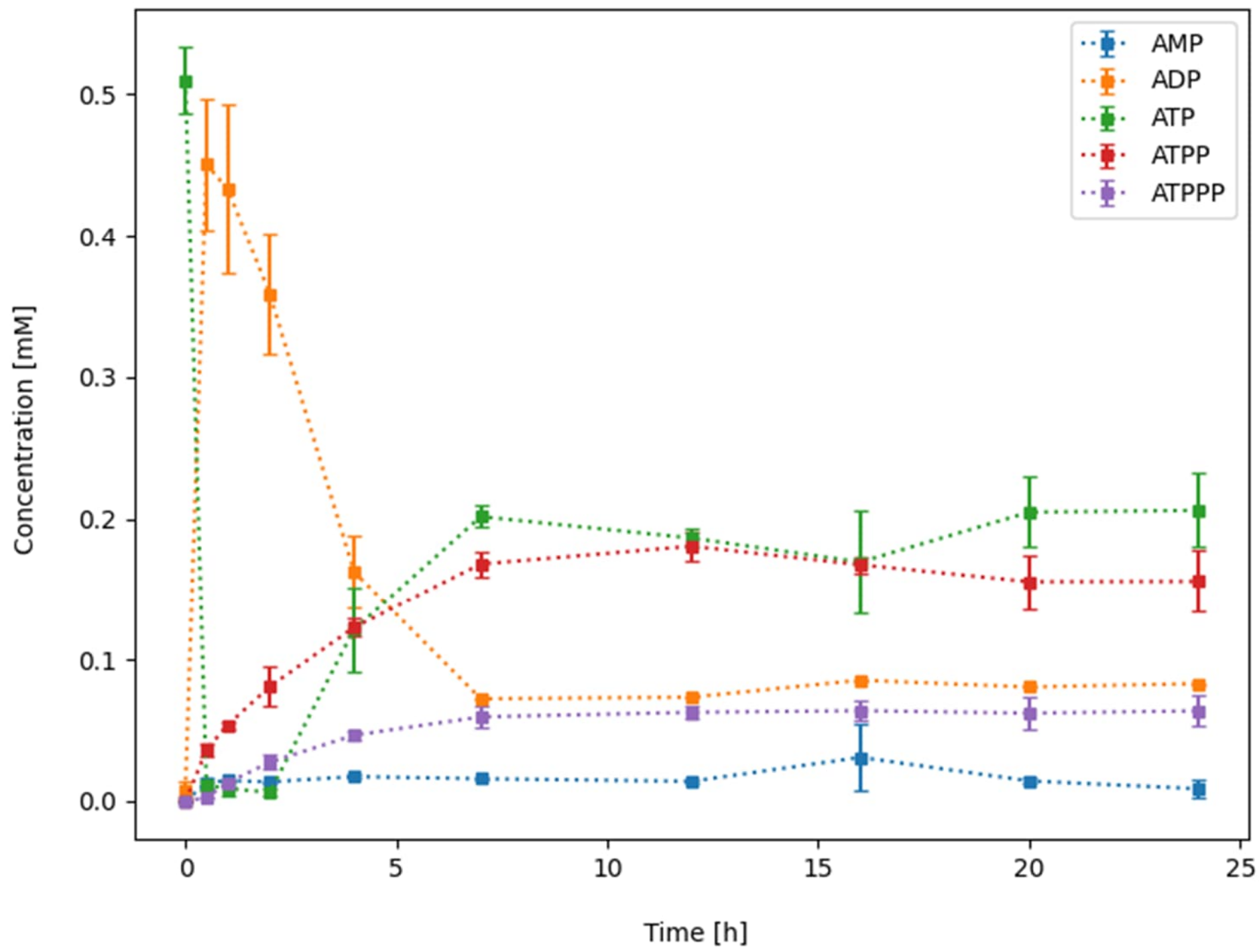

Experiment 4: UDP-GalNAc Conf. 36 (50 mM)

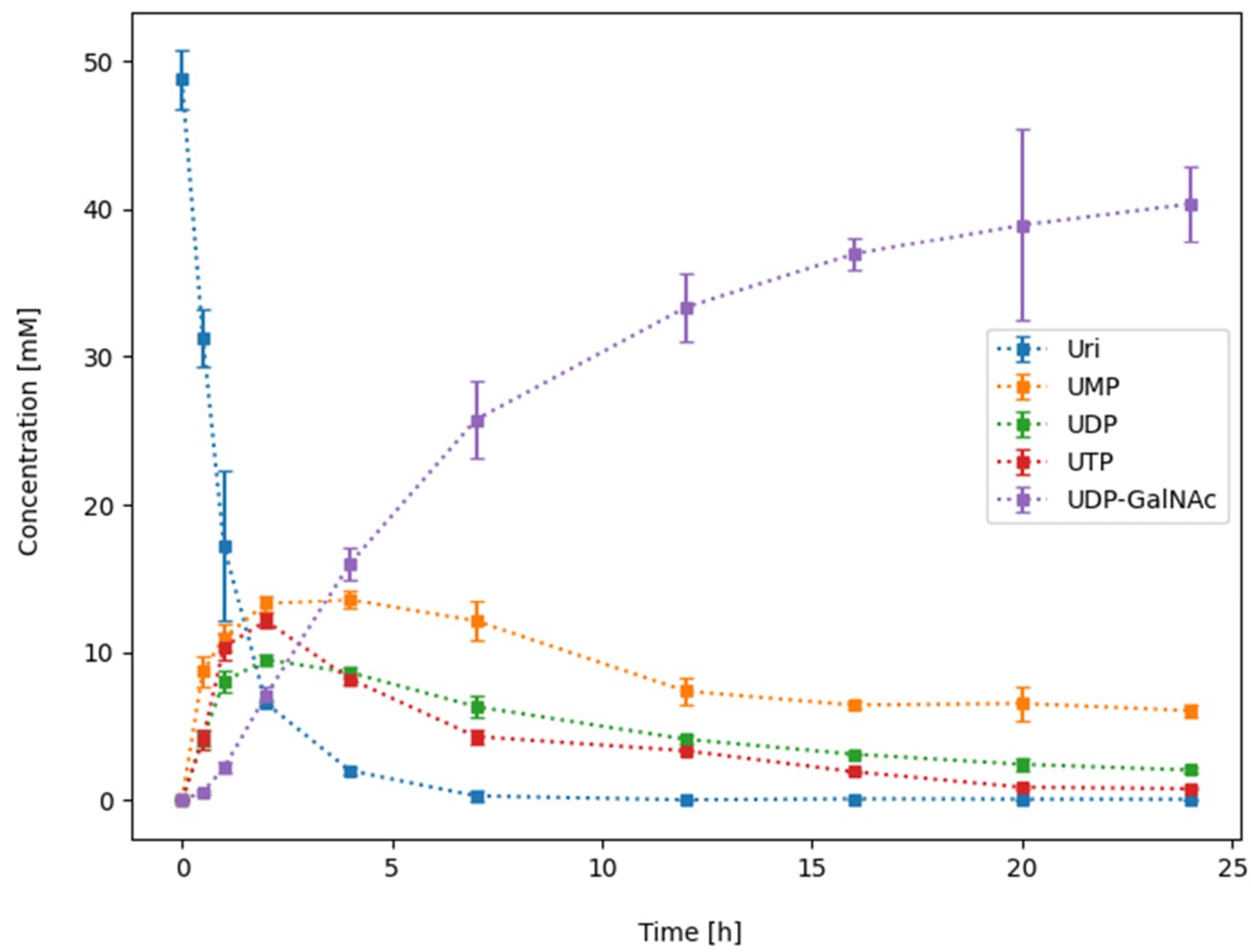

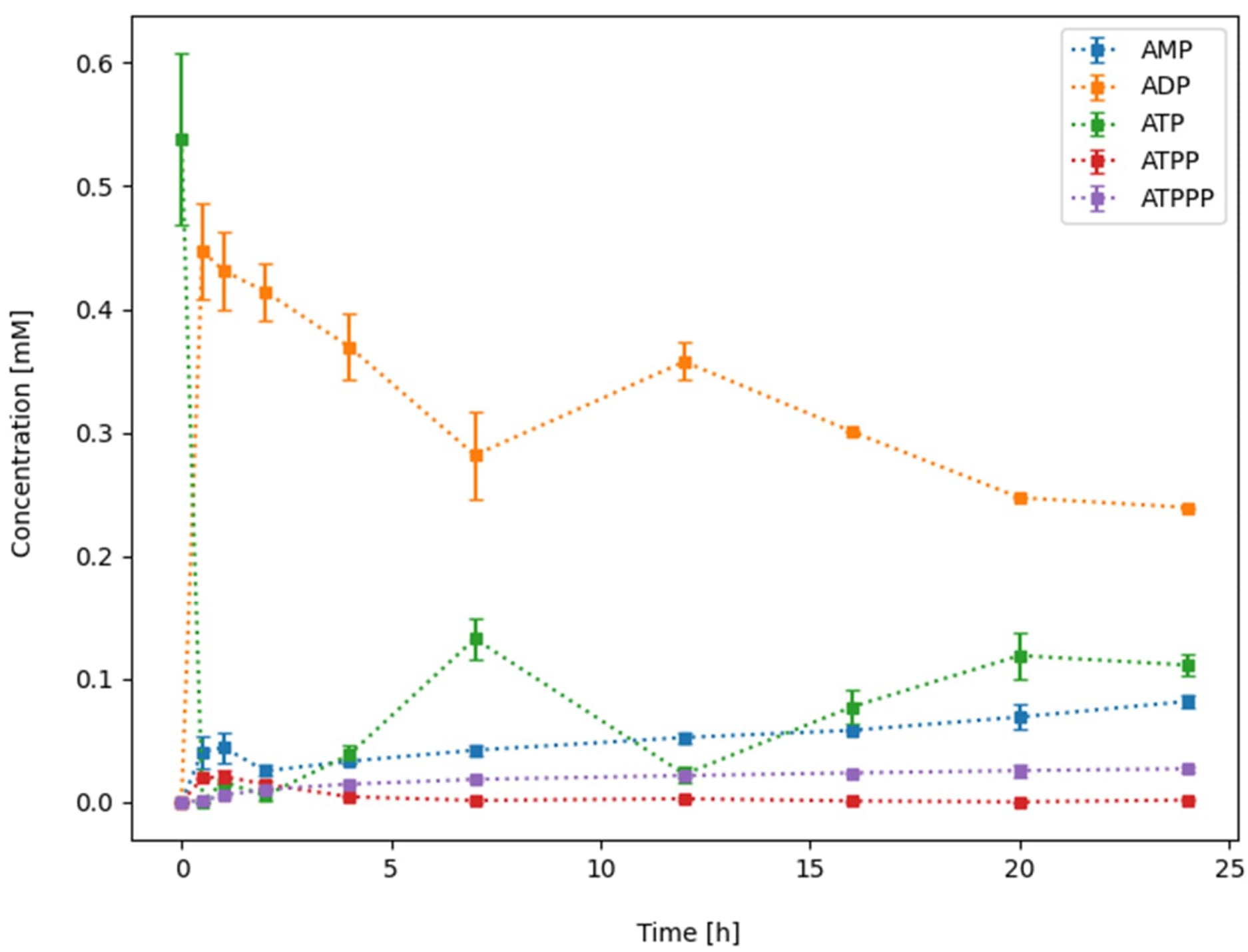

Experiment 5: UDP-GalNAc Conf. 37 (20 mM, A)

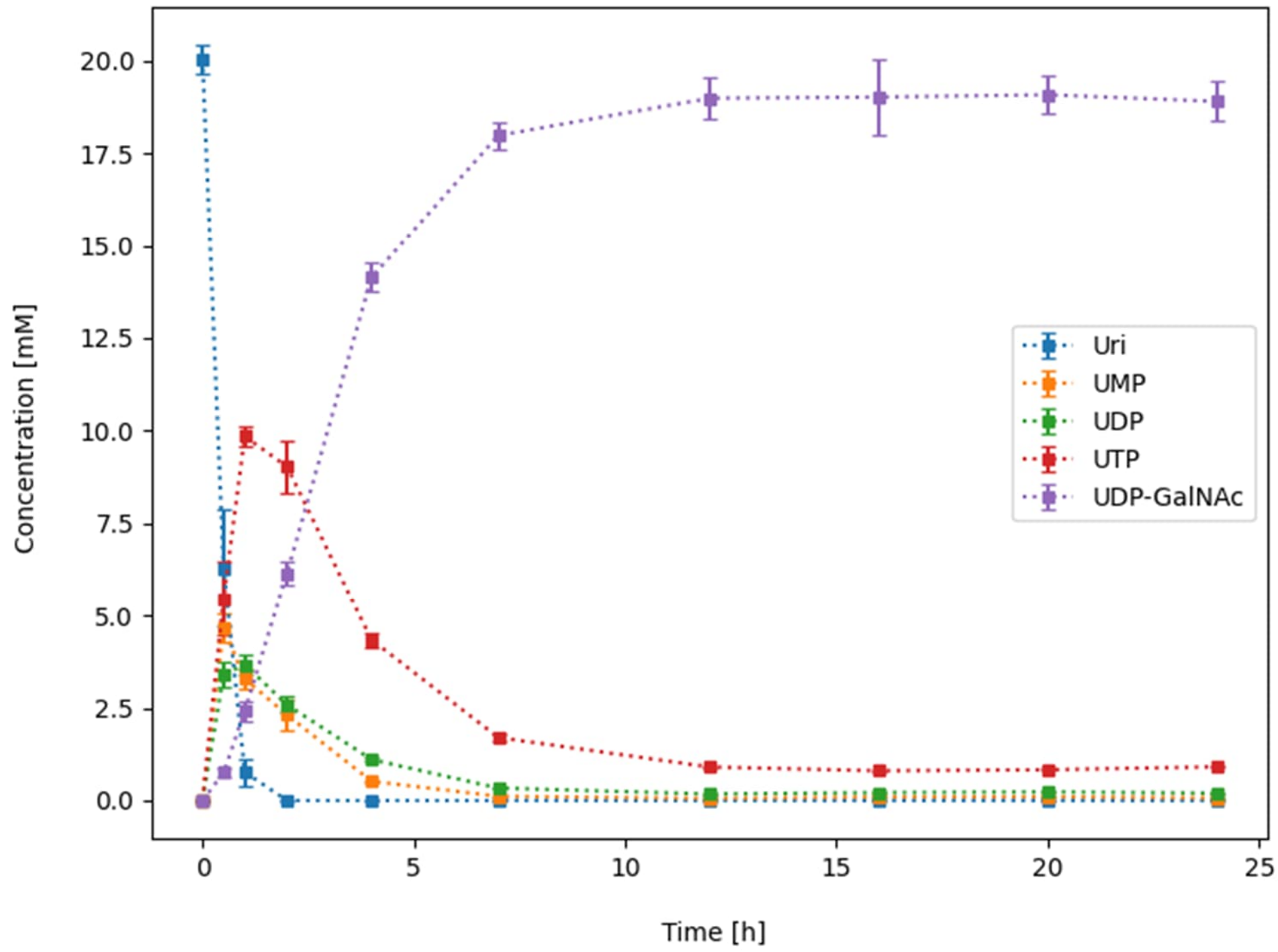

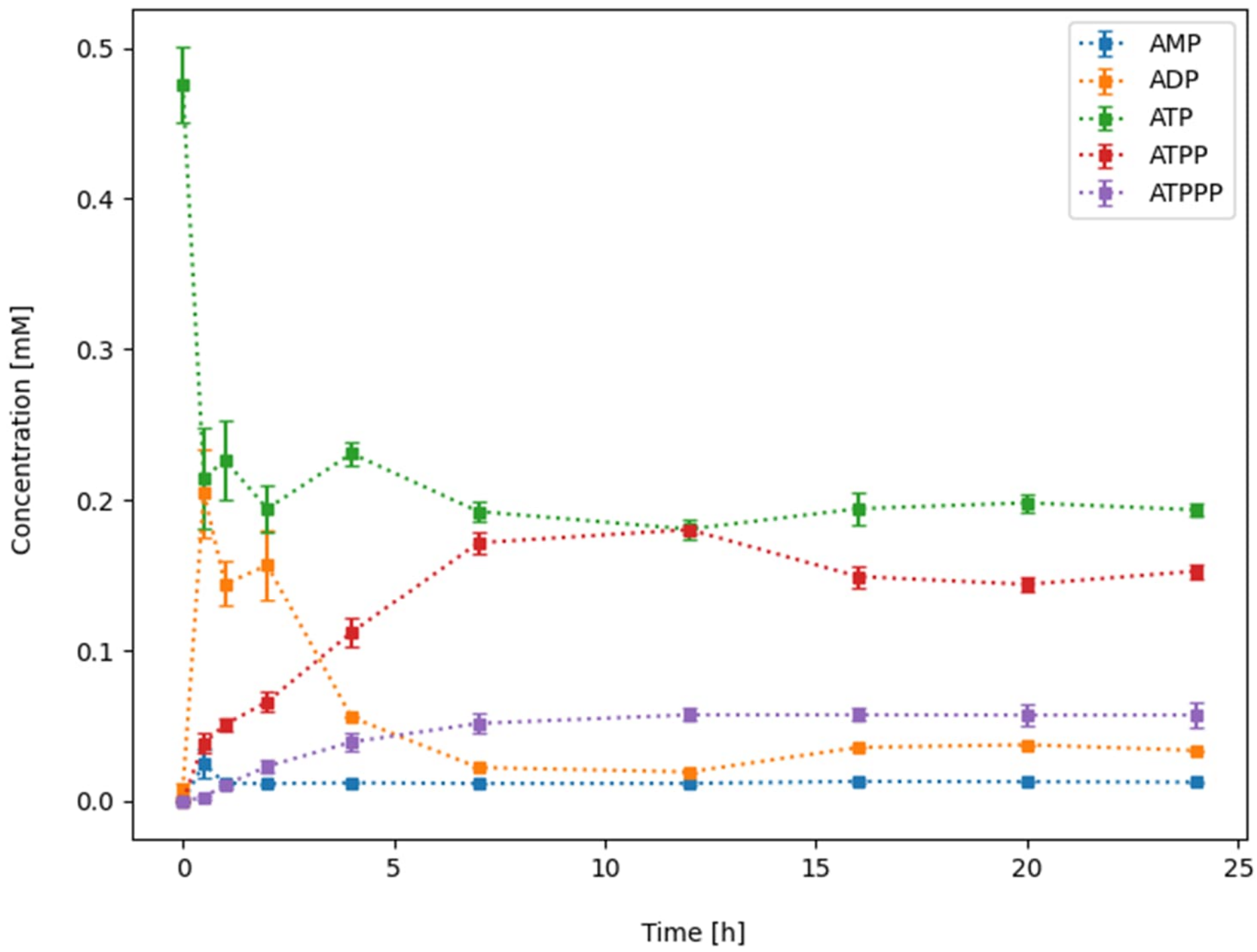

Experiment 6: UDP-GalNAc Conf. 37 (50 mM, A)

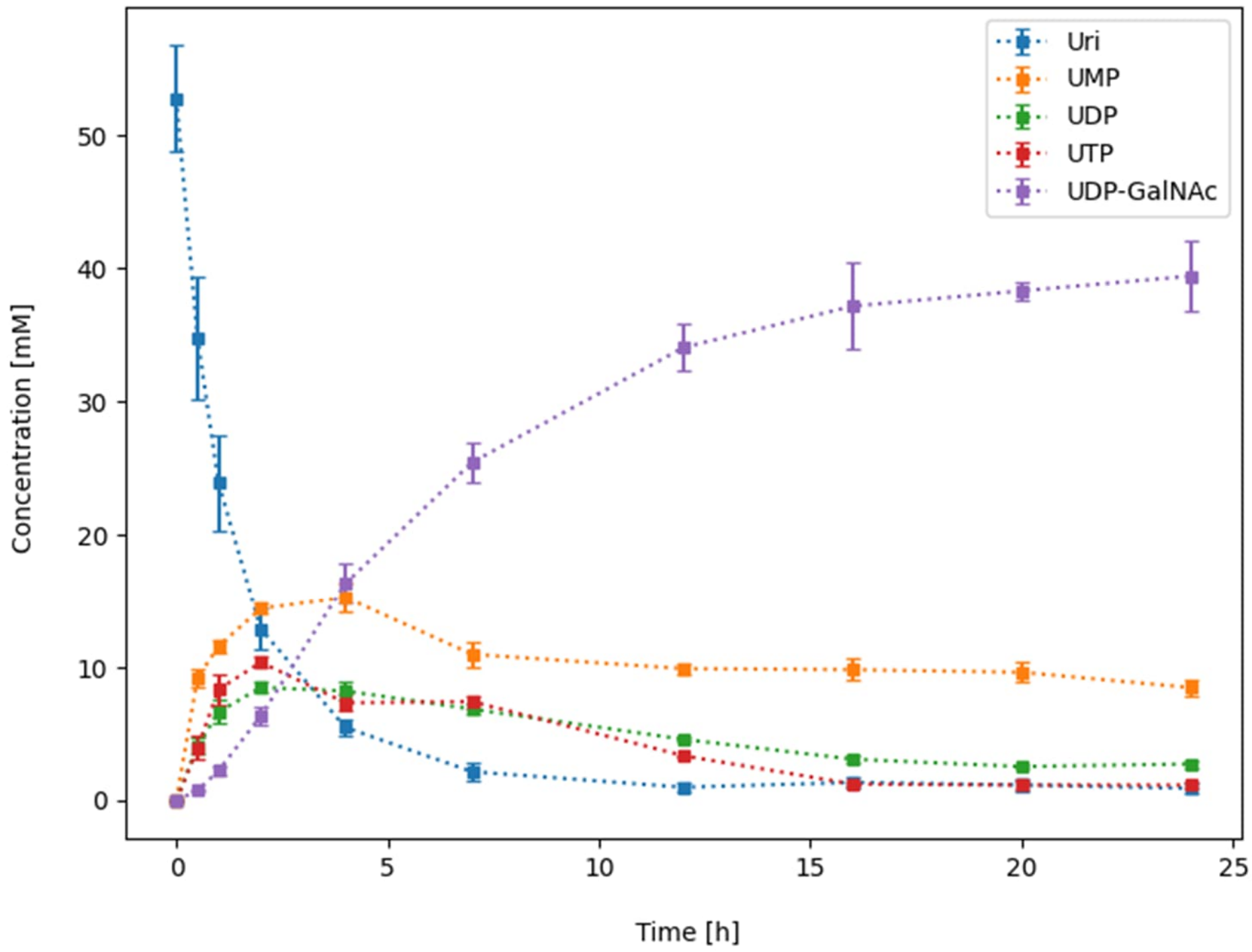

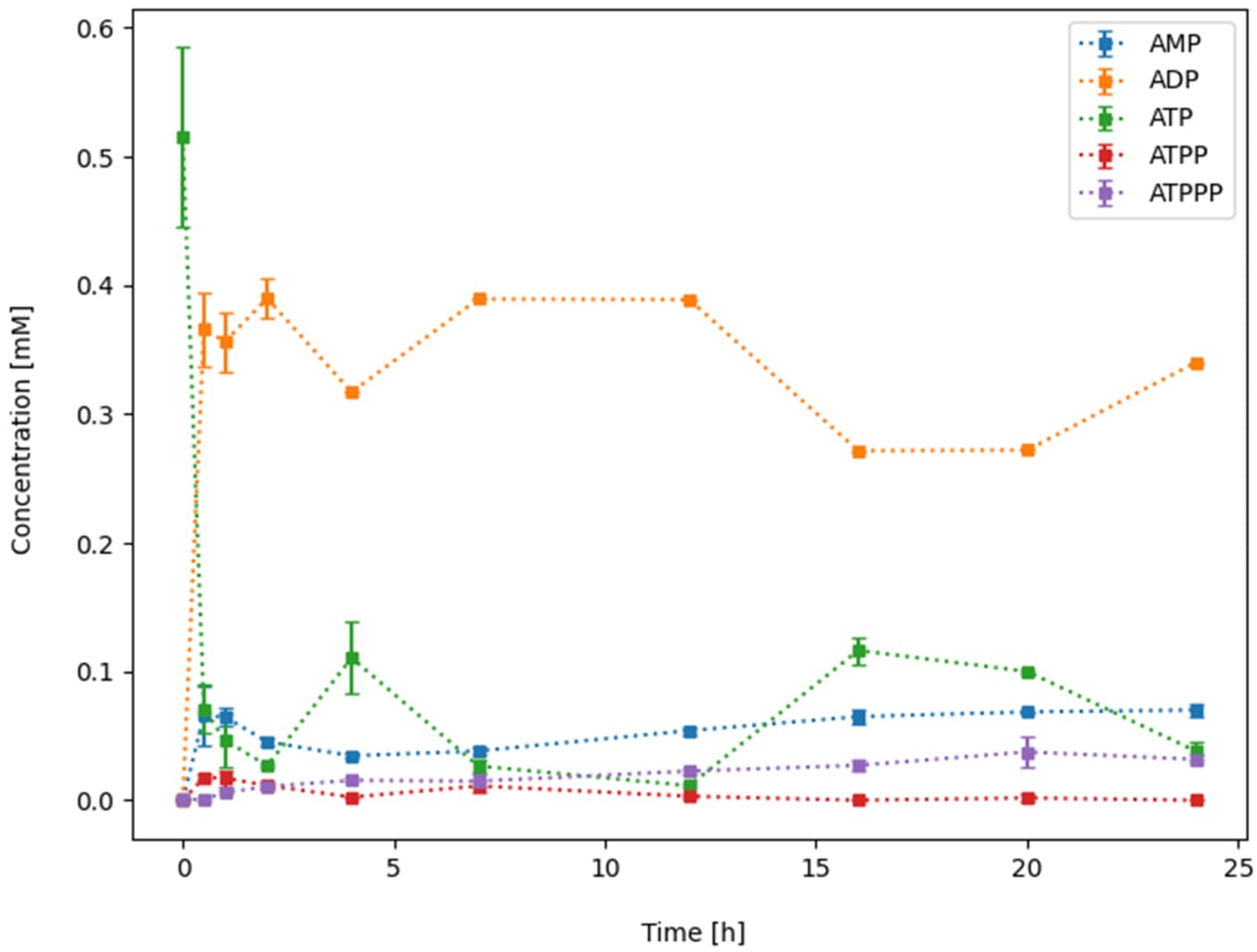

Experiment 7: UDP-GalNAc Conf. 37 (20 mM, B)

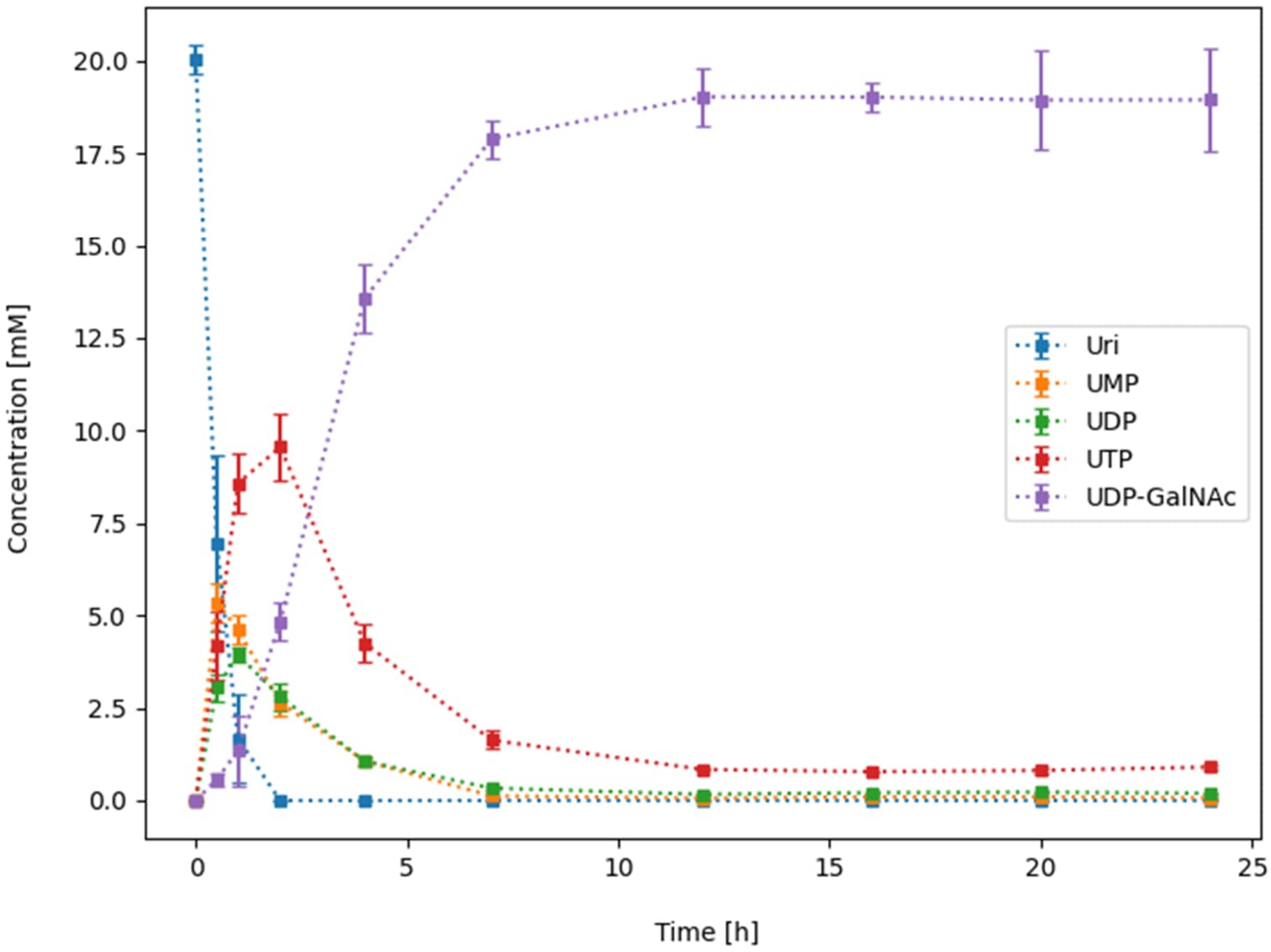

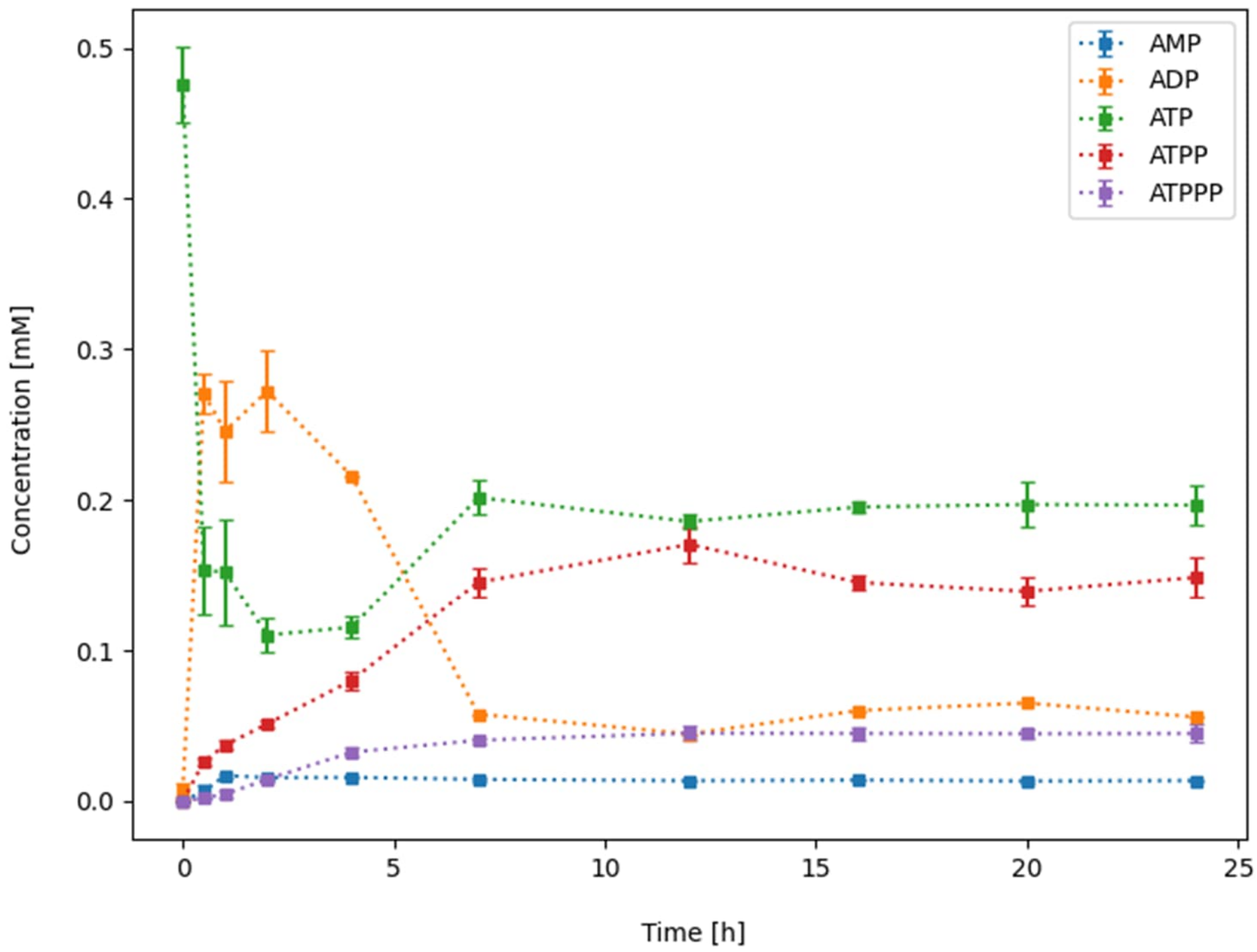

Experiment 8: UDP-GalNAc Conf. 37 (50 mM, B)

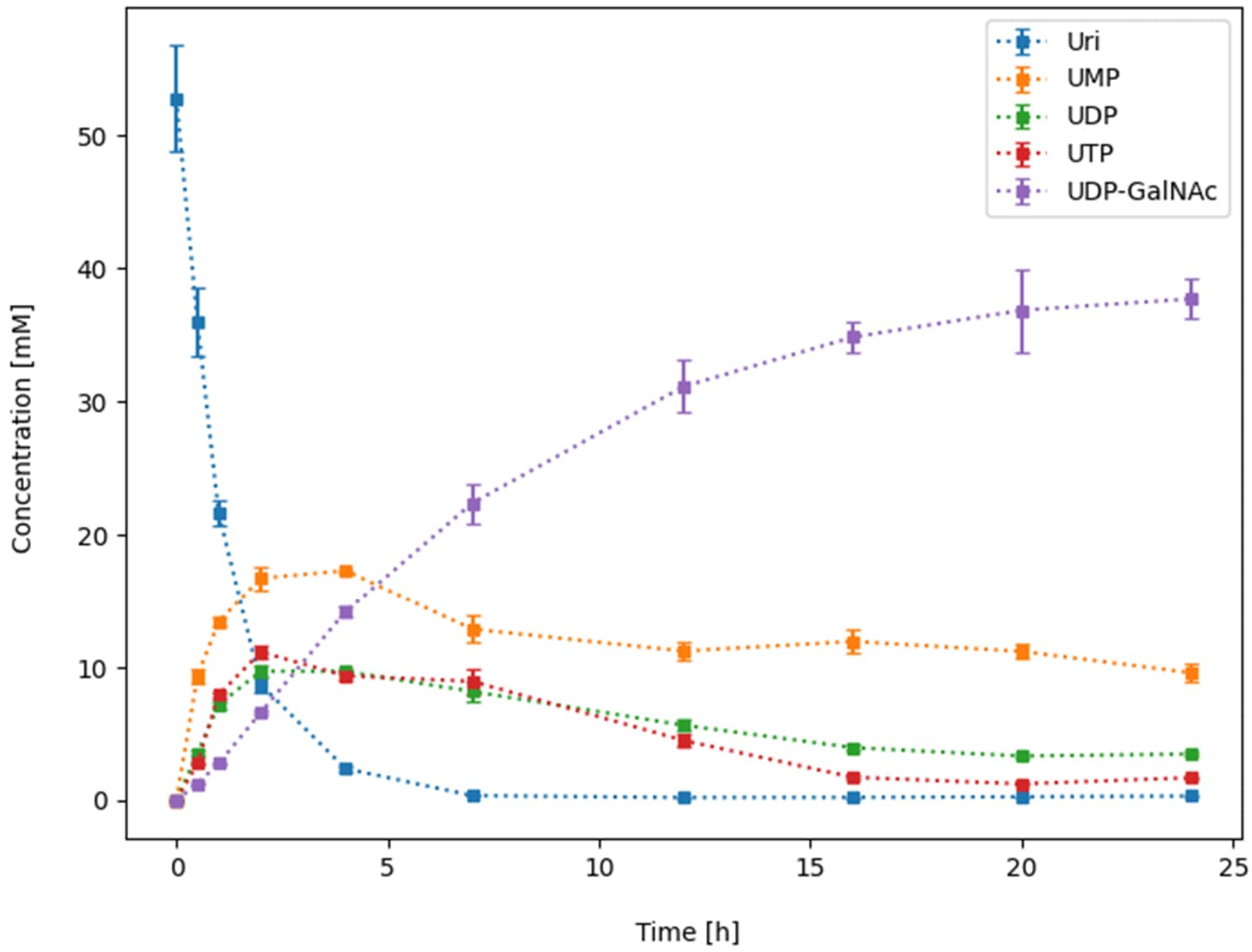

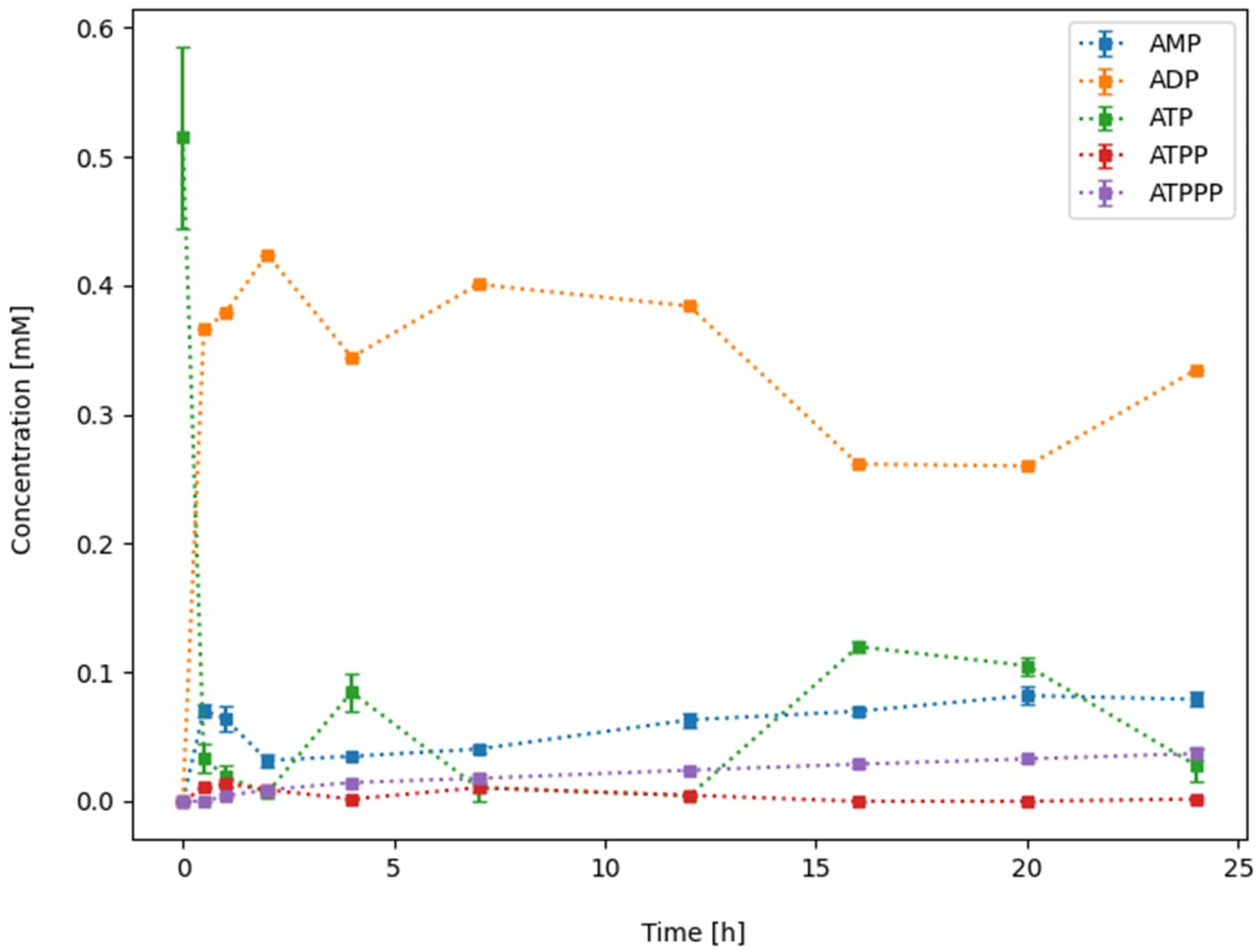

Experiment 9: UDP-GalNAc Conf. 39 (V1)

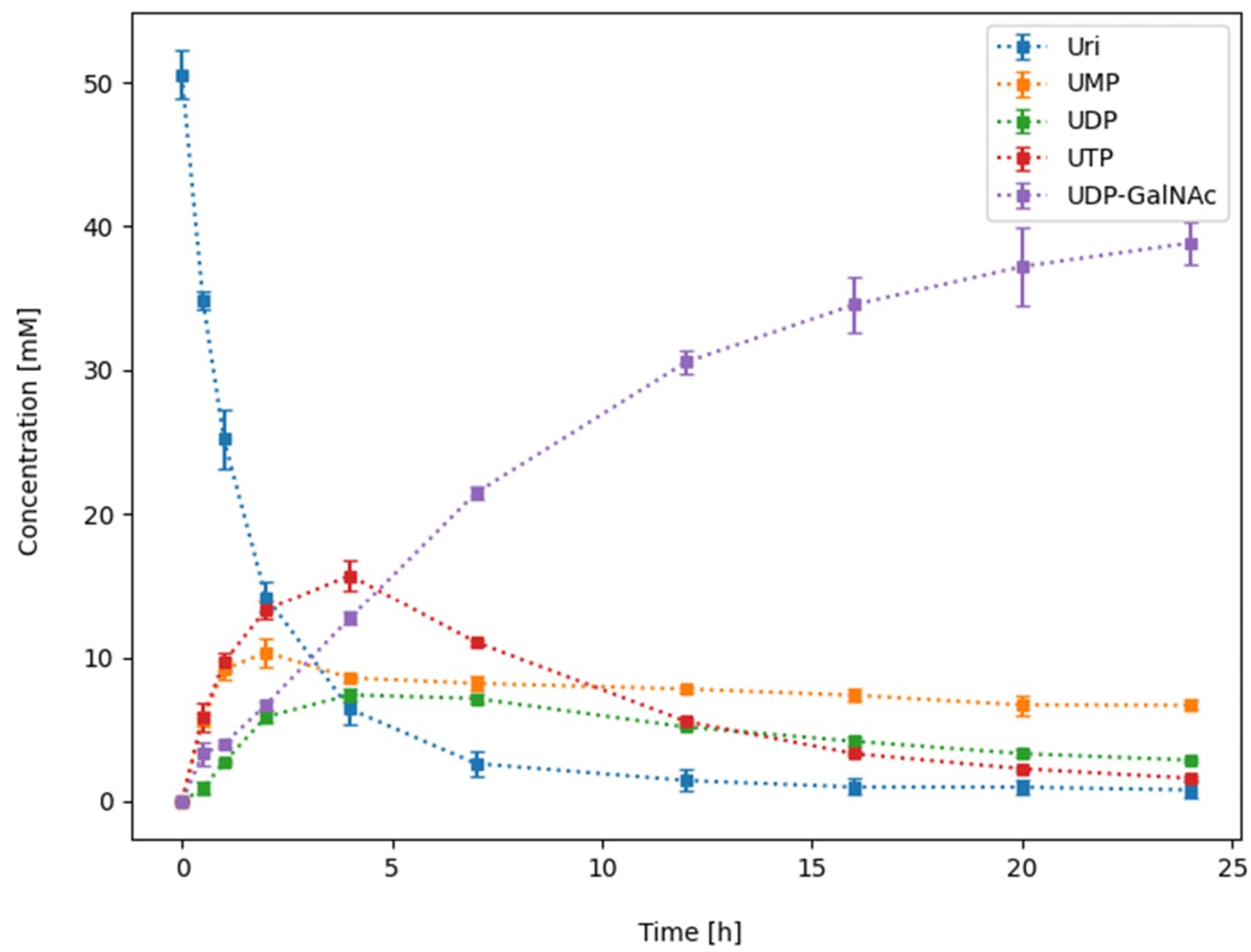

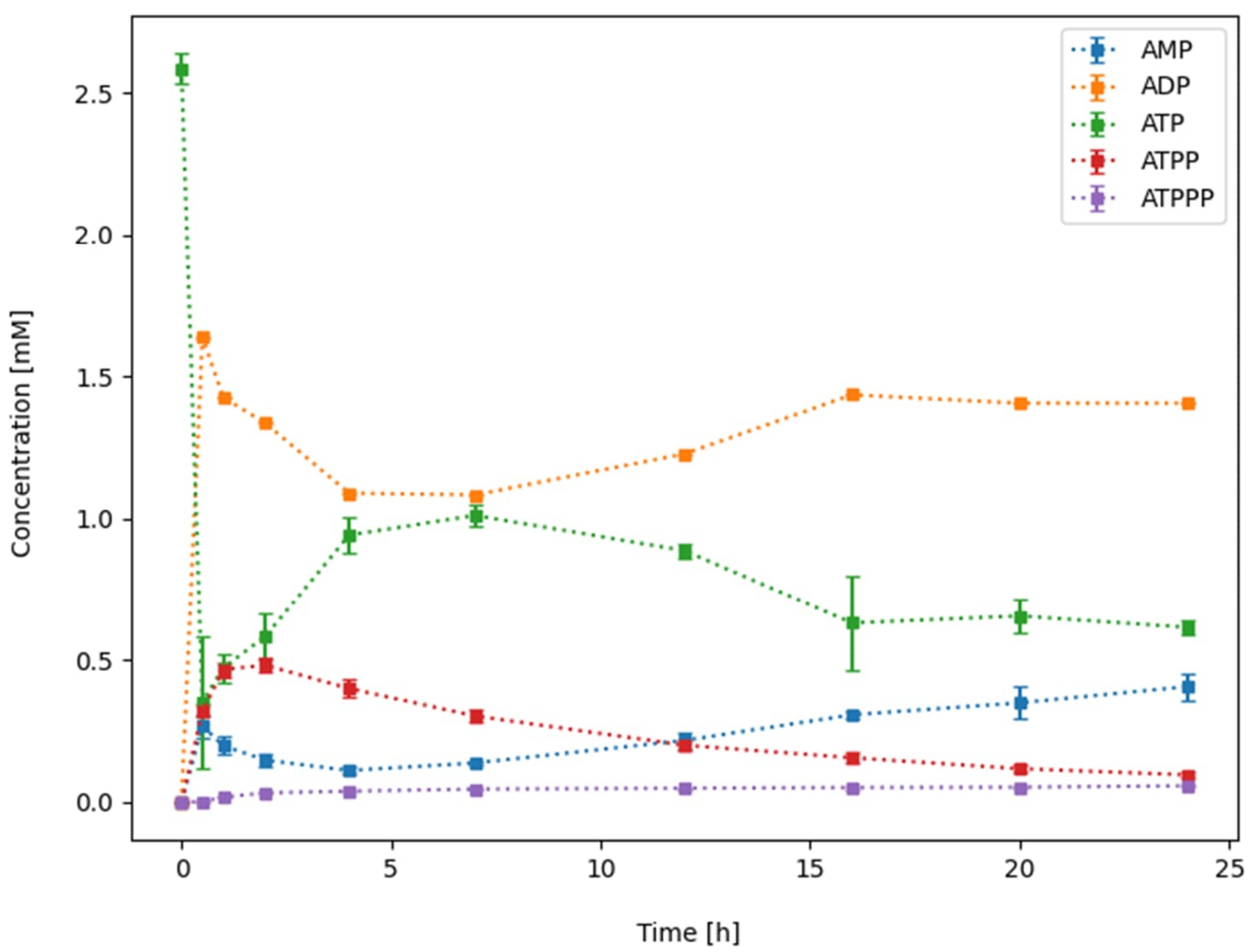

Experiment 10: UDP-GalNAc Conf. 39 (V2)

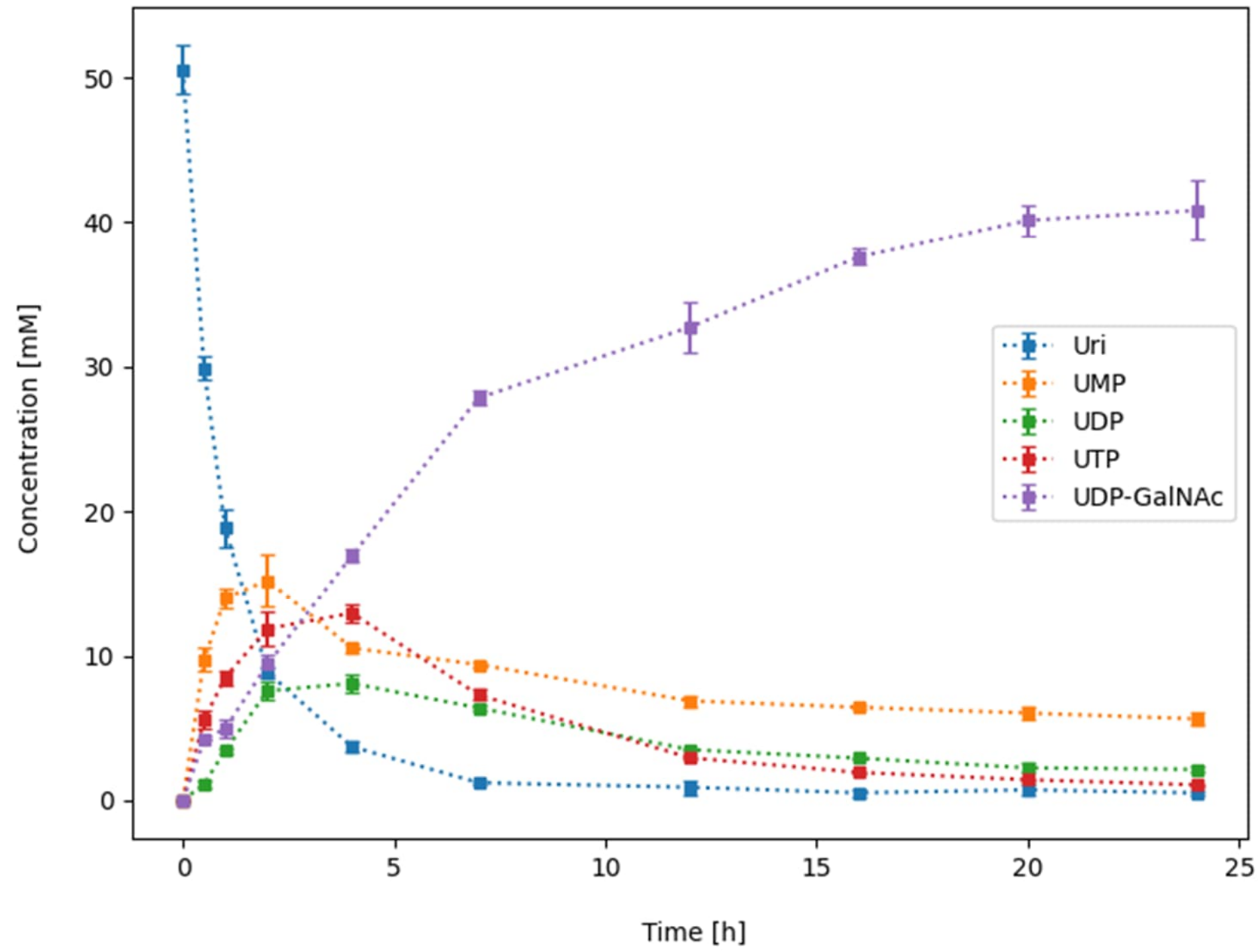

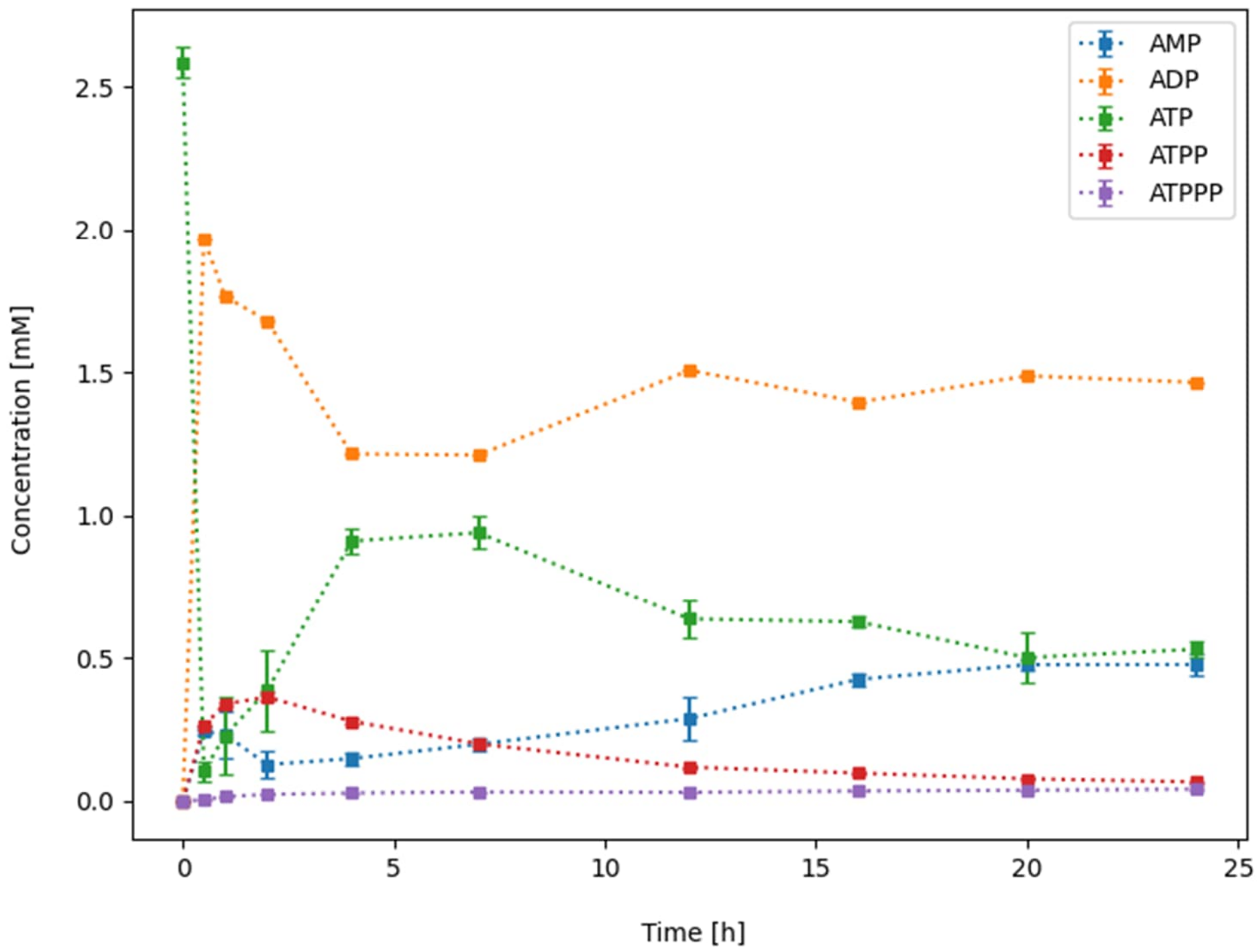

Experiment 11: UDP-GalNAc Conf. 39 (V3)

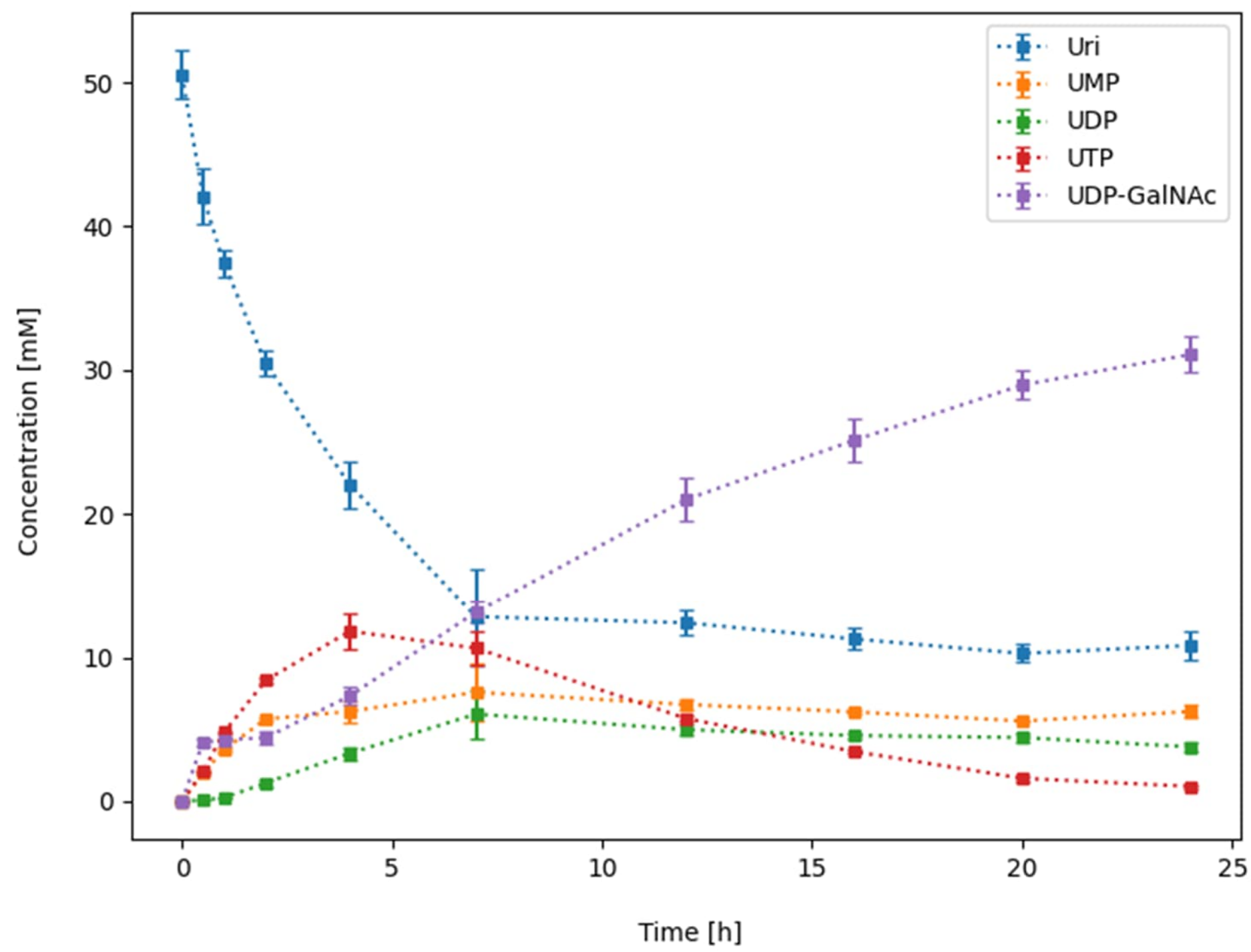

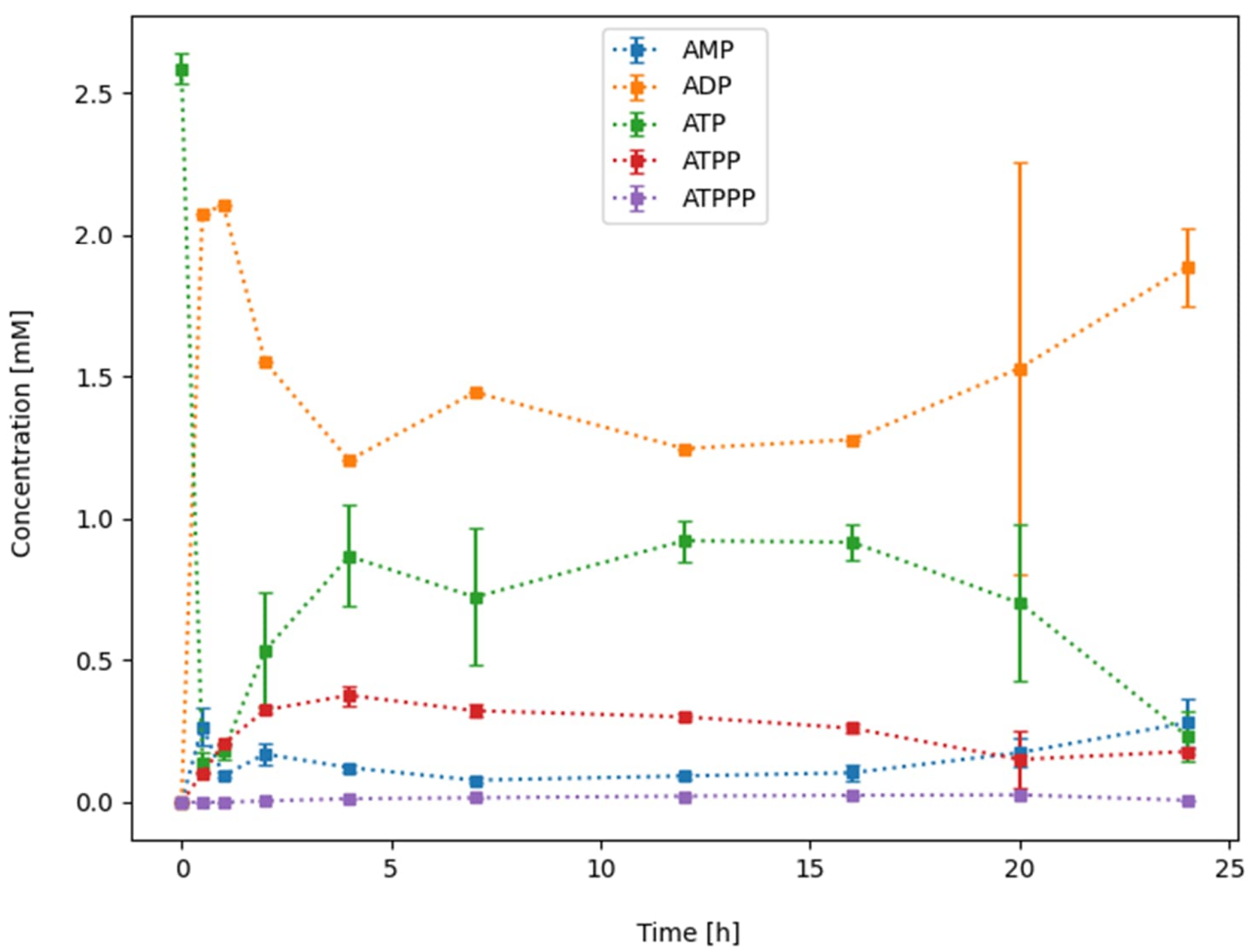

Experiment 12: UDP-GalNAc Conf. 39 (V4)

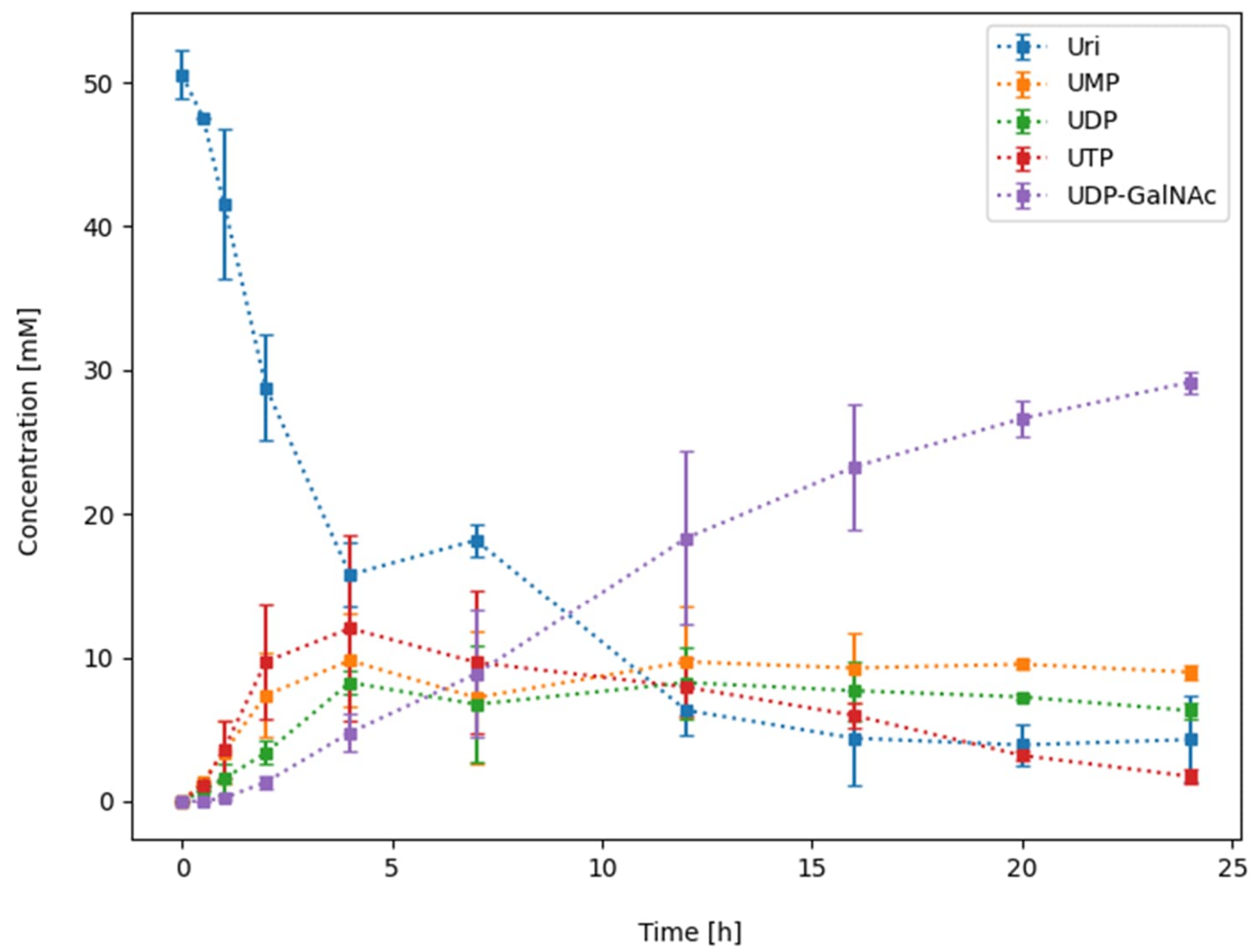

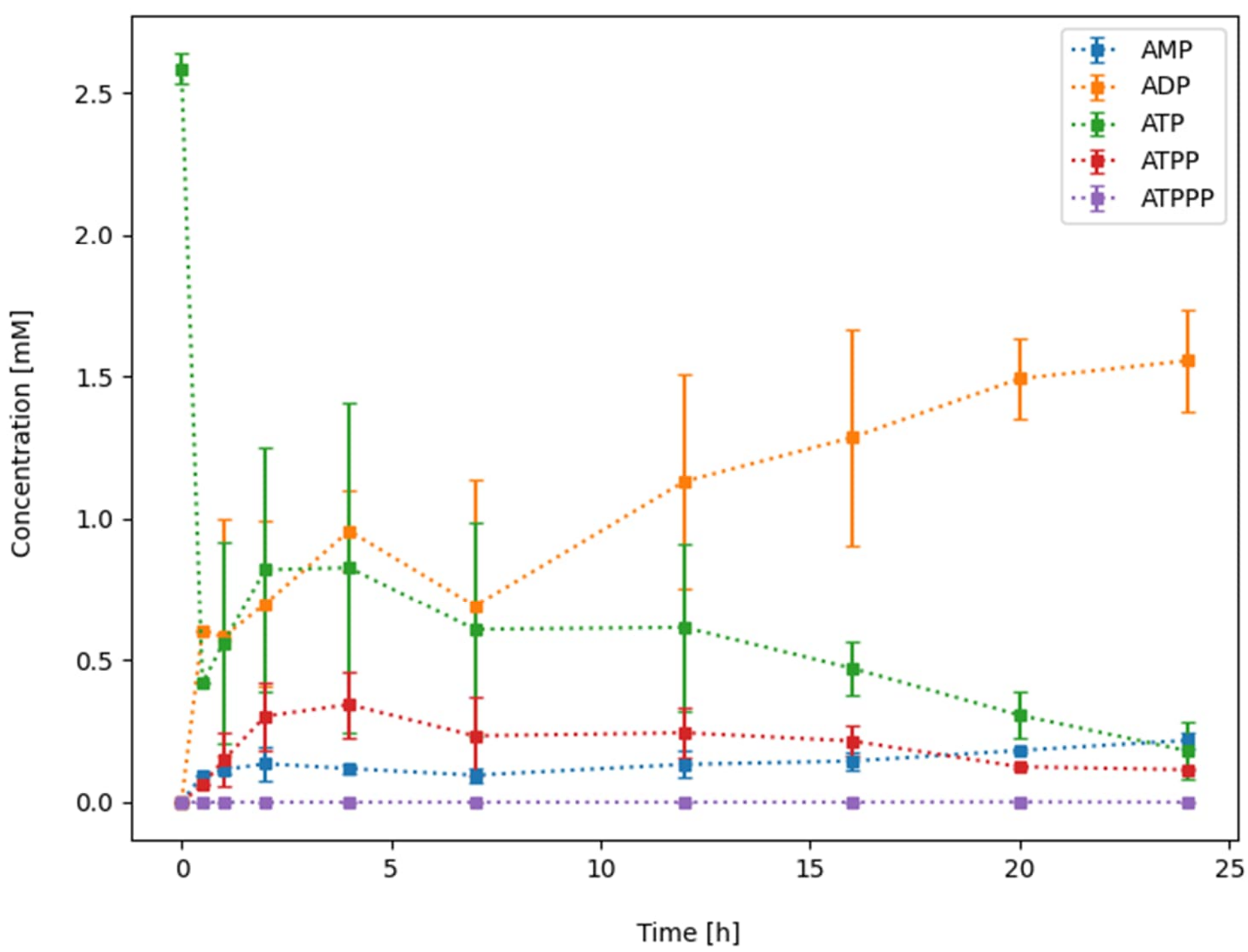

<u>Experiment 13: UDP-GalNAc Conf. 40 (TO, 7h)</u>

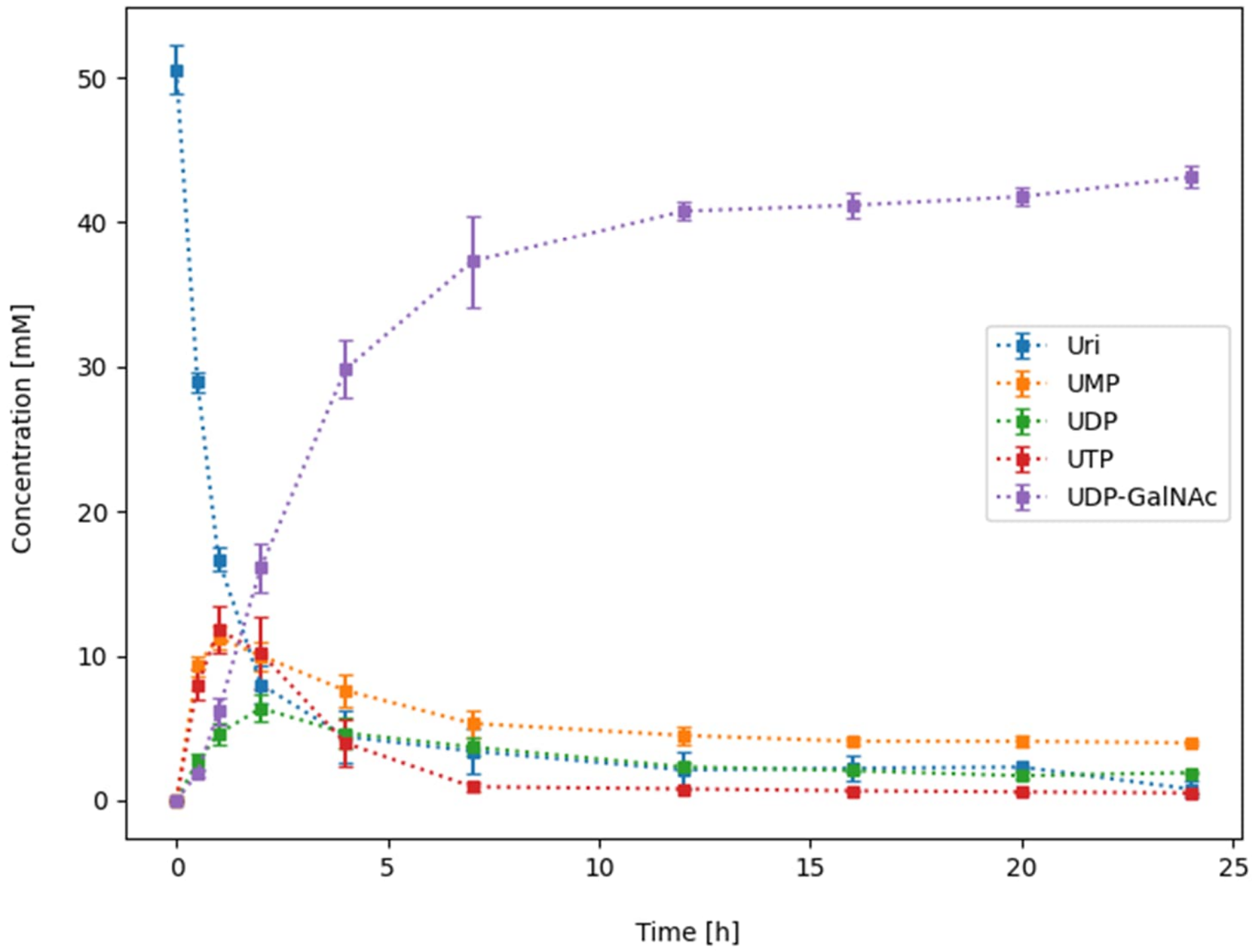

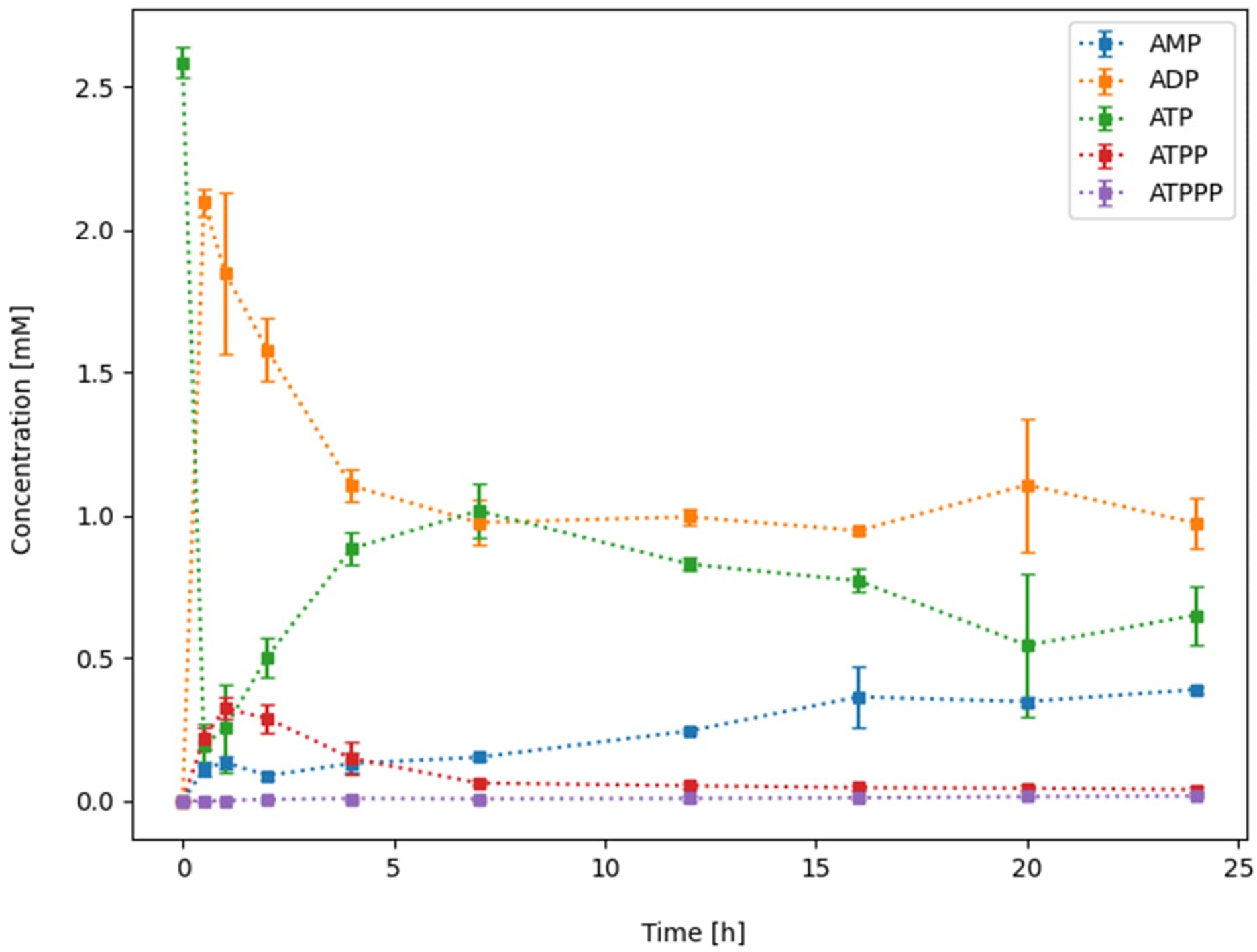

Experiment 14: UDP-GalNAc Conf. 40 (LO)

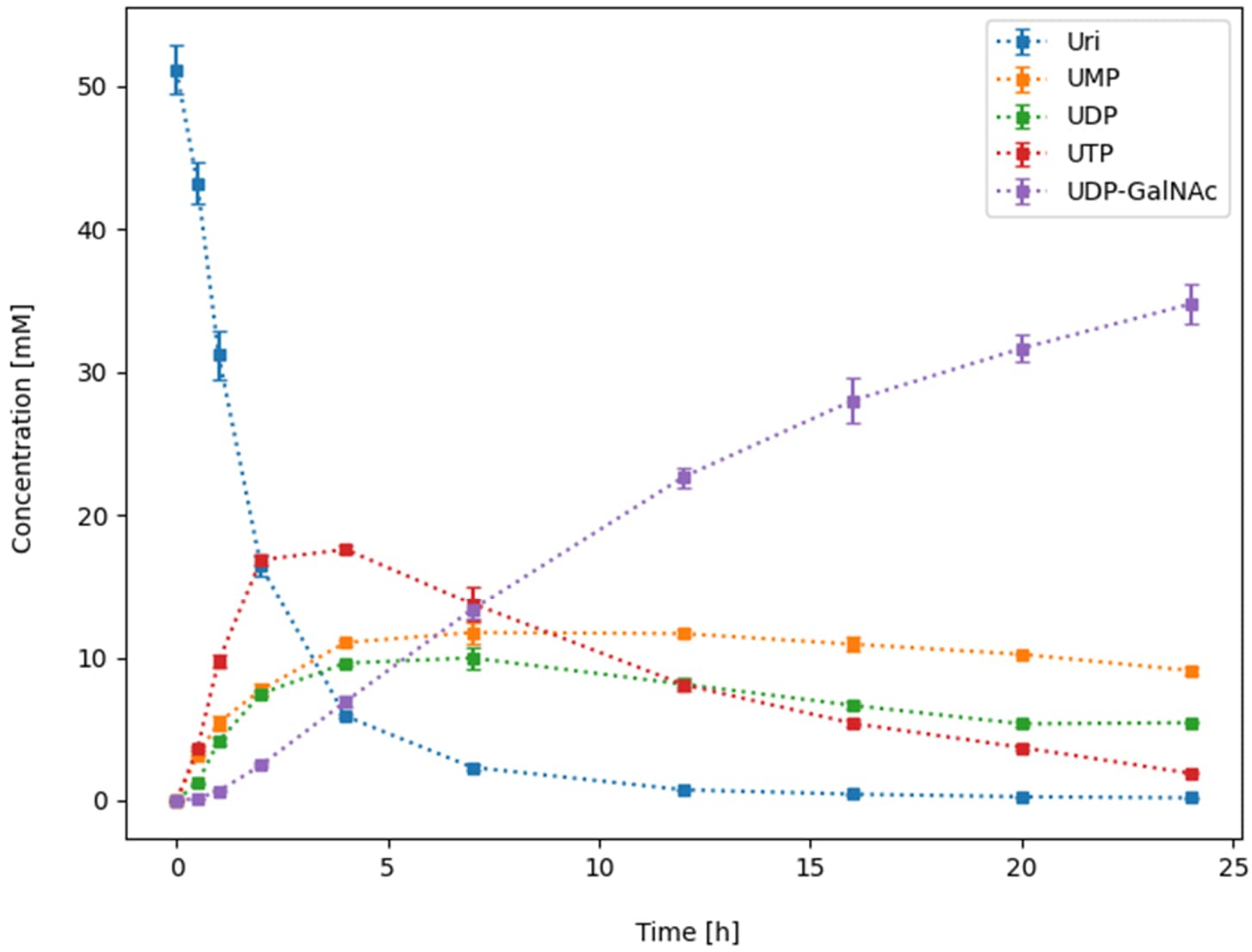

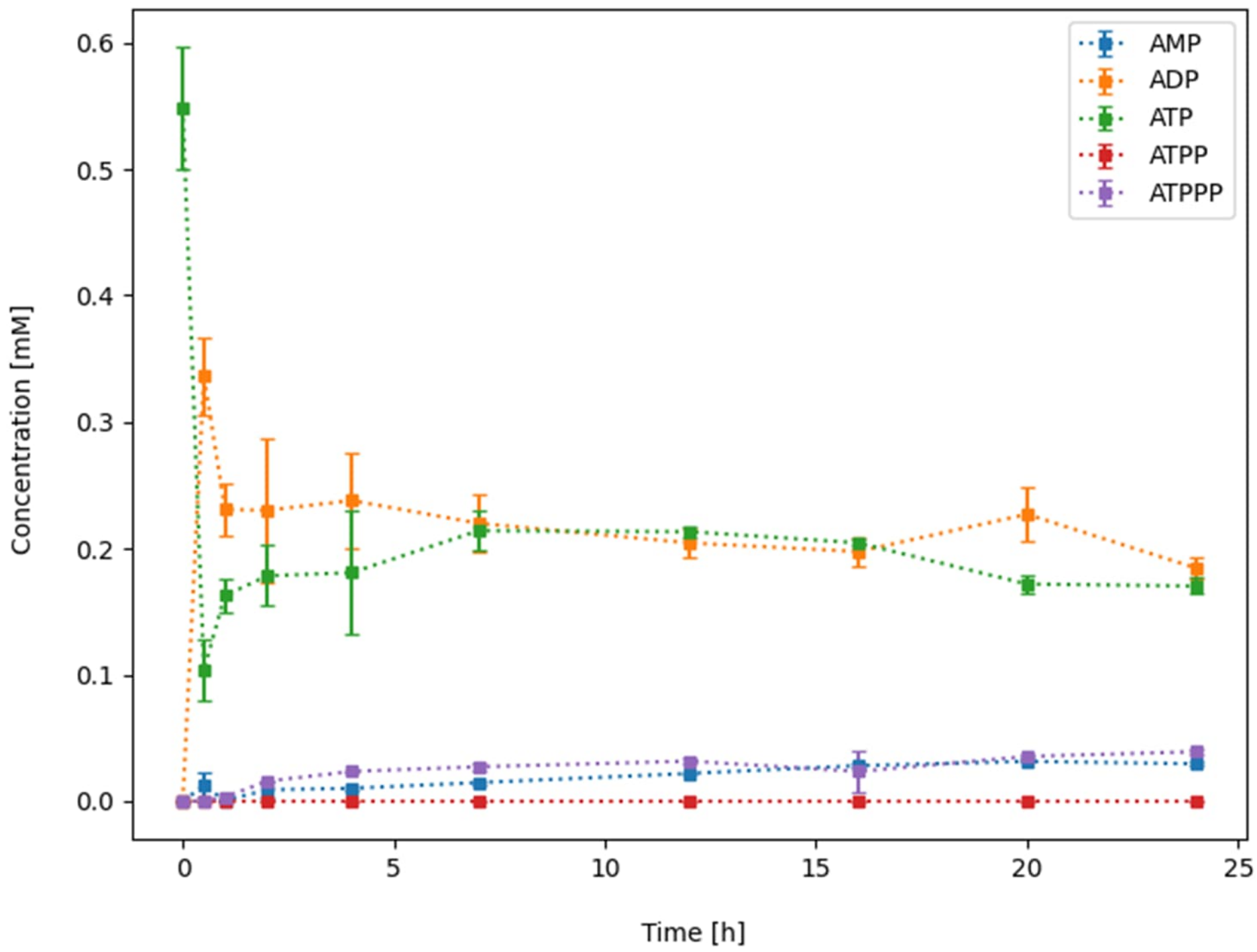

Supplement: Supplementary file 1 [file mmc1.pdf]
