# Supplementary material for: Combining automated kinetic model selection with robust optimization and application to maximize cell-free production of UDP-GalNAc
Source: Metab Eng Commun. 2026 Sep 8;23:e00287. doi: 10.1016/j.mec.2026.e00287 (PMC13634411; doi:10.1016/j.mec.2026.e00287)

Supplementary File S2

Combining Automated Kinetic Model Selection with Robust Optimization and Application to Maximize Cell-free Production of UDP-GalNAc

Nicolas Huber[1,#], Tuan Son Hoang[1,2, #], Sebastián Espinel Ríos[3], Thomas F.T. Rexer[1,4], Udo Reichl[1,2], Steffen Klamt[1, *]

[1] Max Planck Institute for Dynamics of Complex Technical Systems, 39106 Magdeburg, Germany

[2] Otto von Guericke University Magdeburg, 39106 Magdeburg, Germany

[3] School of Chemical and Bioprocess Engineering, University College Dublin, D04 V1W8, Dublin, Ireland

[4] eversyn, 39106 Magdeburg, Germany

*Corresponding author: klamt@mpi-magdeburg.mpg.de

#Equal contribution

Supplementary File S2. Comparisons of model fits between the initial core model and the selected model variant for all data sets

Trajectories (dashed lines: initial core model; solid lines: selected model variant) show the average behavior across all parameter sets of the ensemble generated for the respective model (n = 50), shaded regions show the 95 % confidence interval. Data points are from the respective experimental data sets. GalNAc and GalNAc1P could not be measured over time.

Comparison of model variants with data from experiment 1

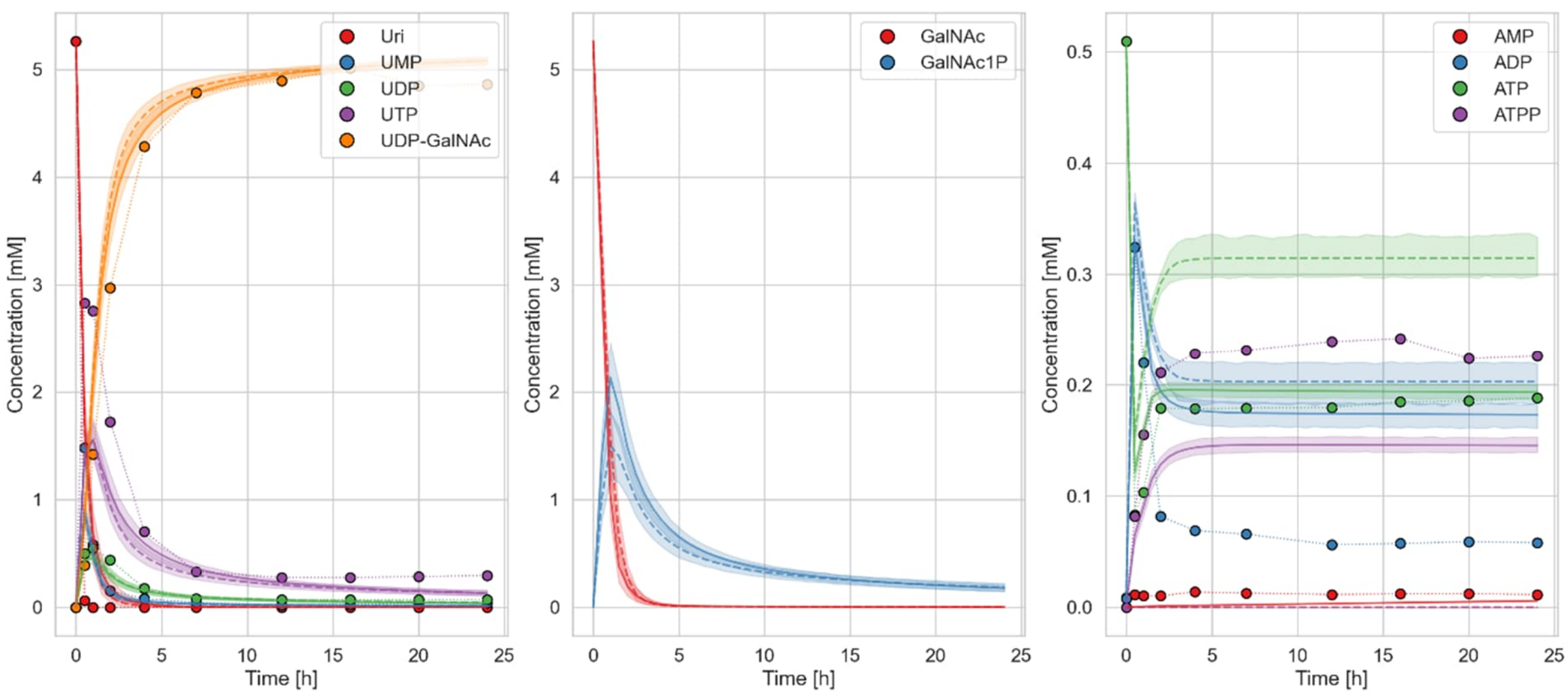

Comparison of model variants with data from experiment 2

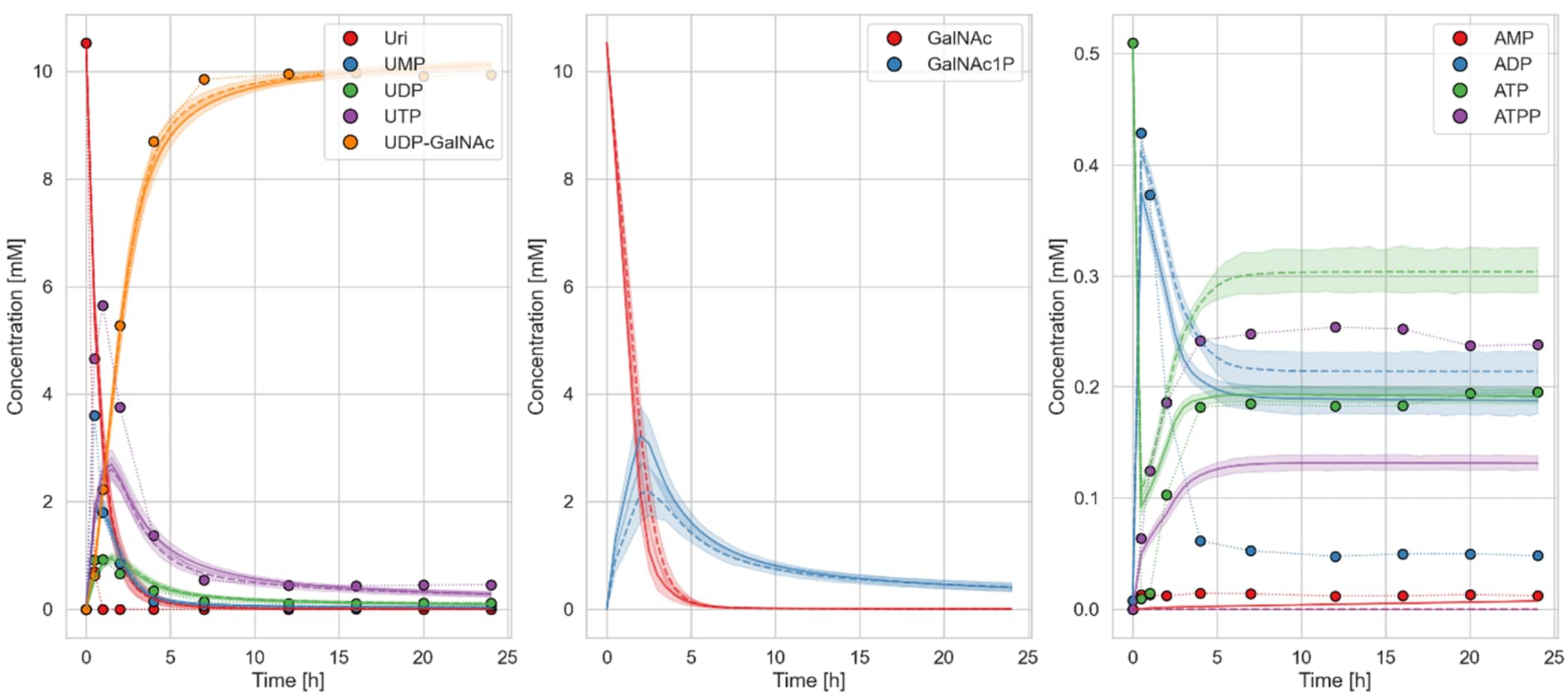

Comparison of model variants with data from experiment 3

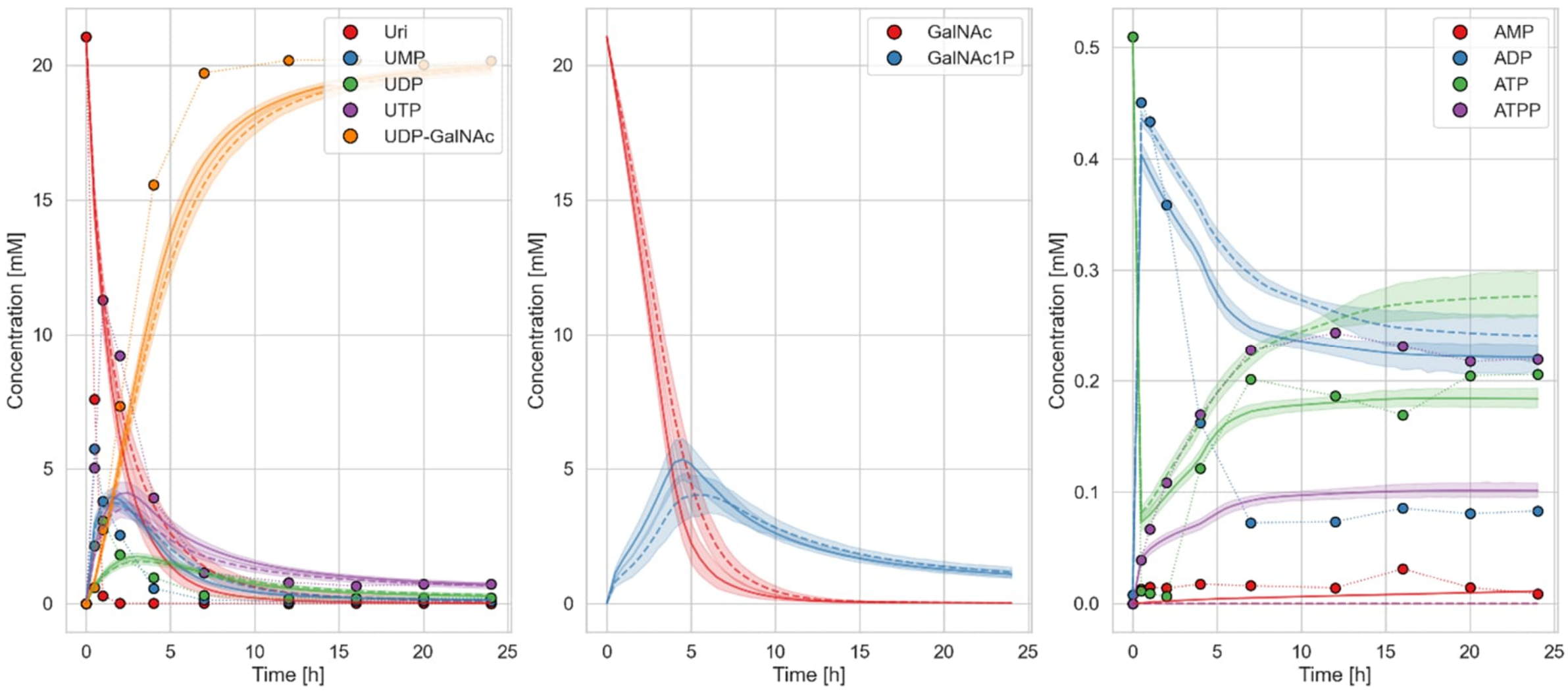

Comparison of model variants with data from experiment 4

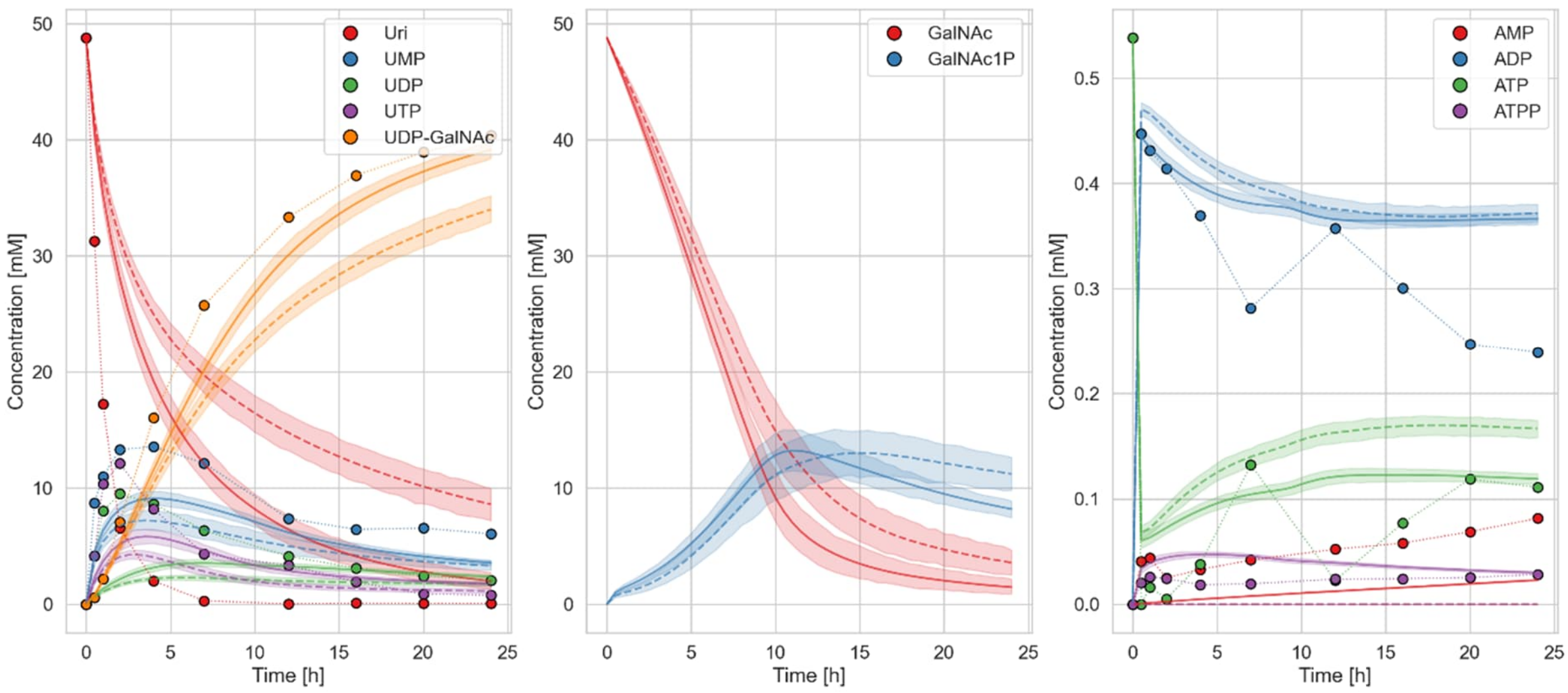

Comparison of model variants with data from experiment 5

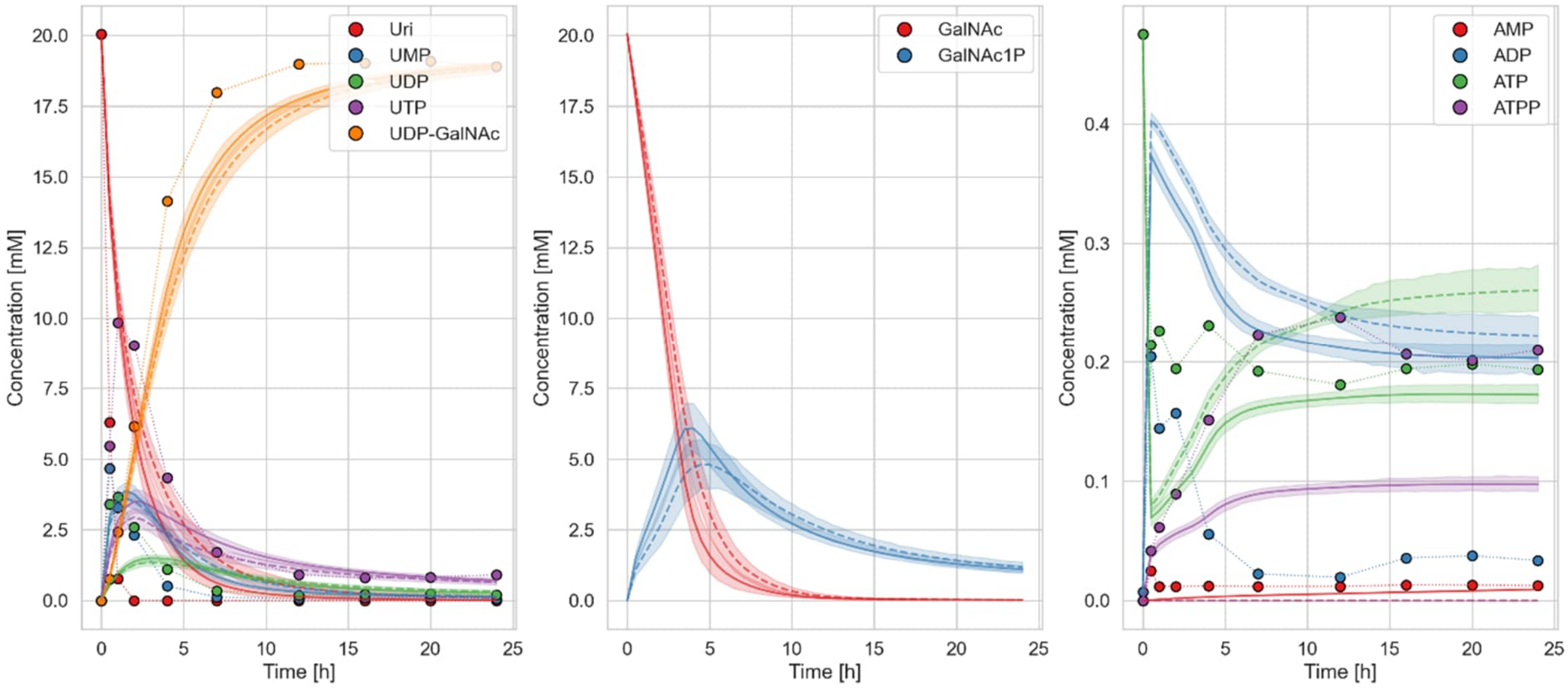

Comparison of model variants with data from experiment 6

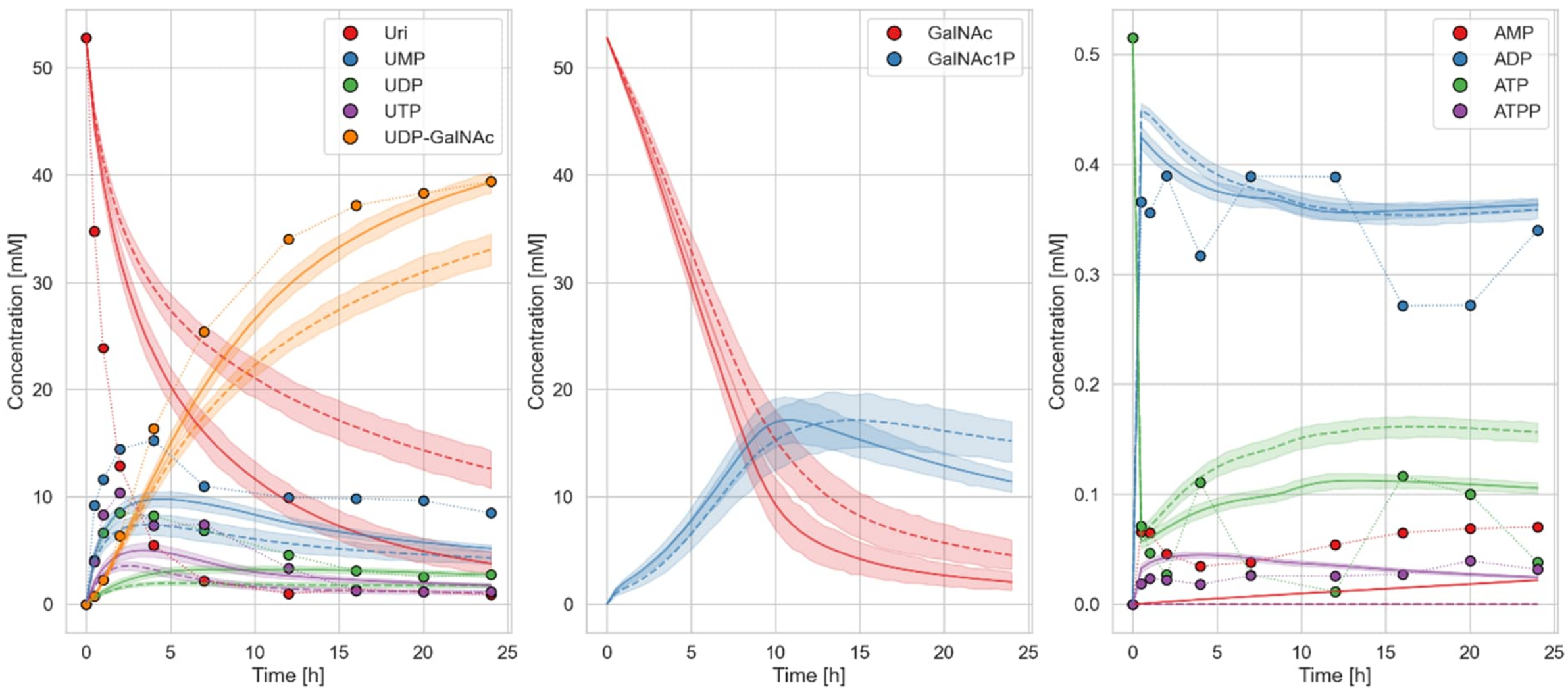

Comparison of model variants with data from experiment 7

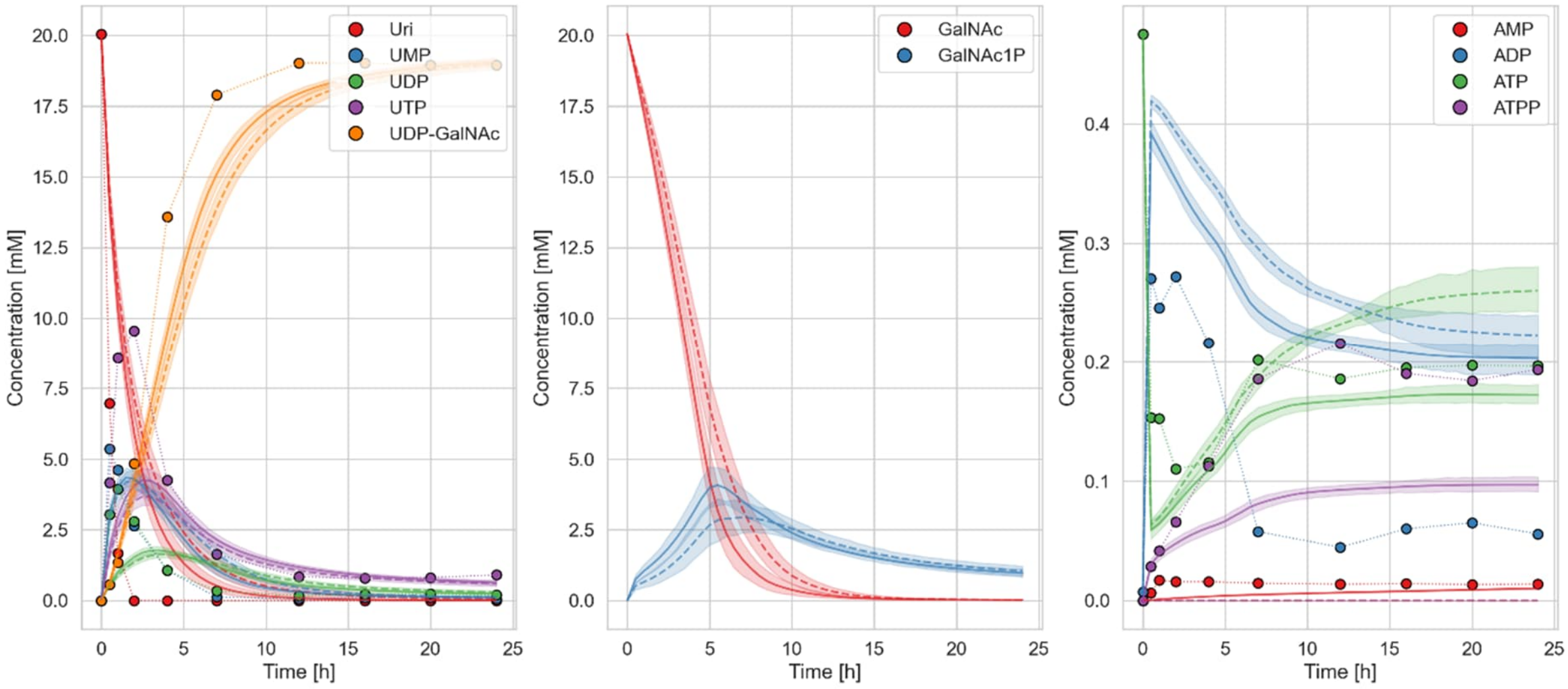

Comparison of model variants with data from experiment 8

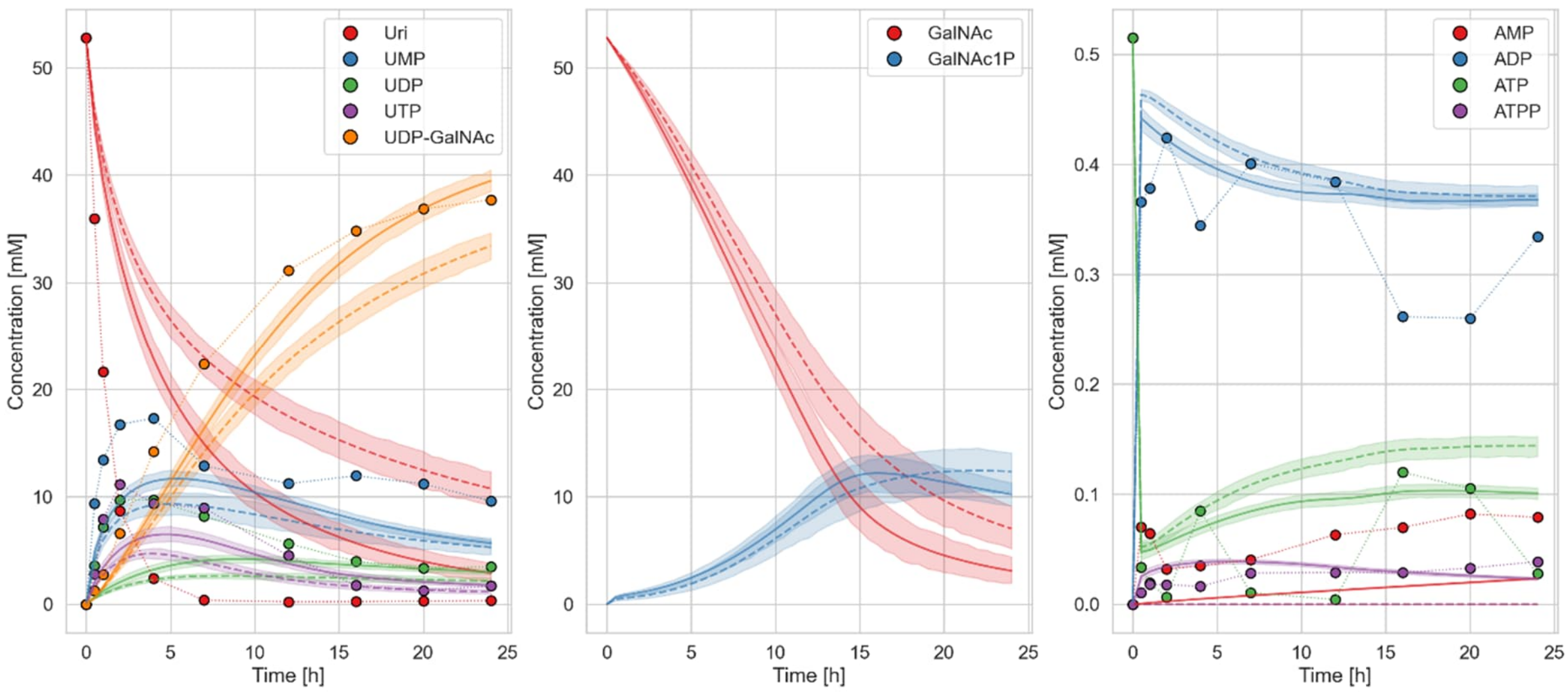

Comparison of model variants with data from experiment 9

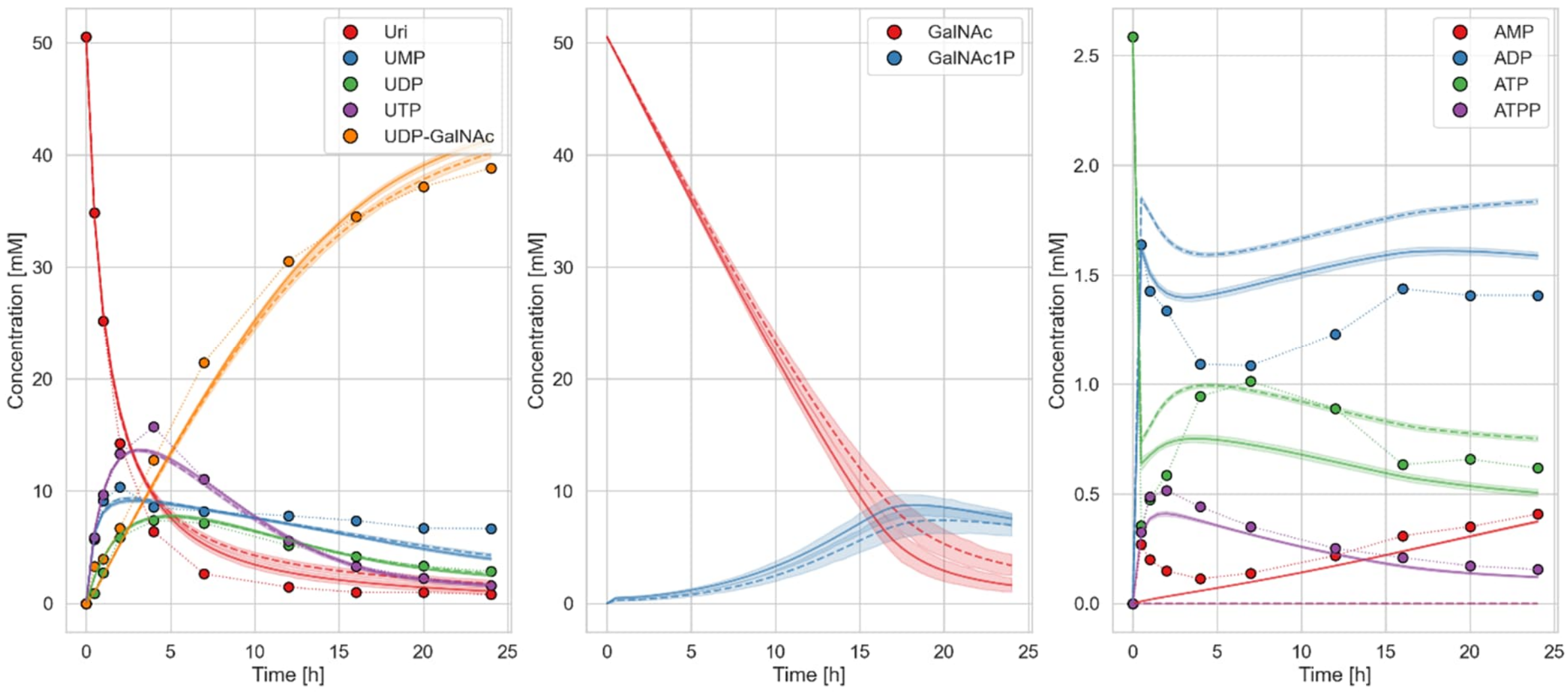

Comparison of model variants with data from experiment 10

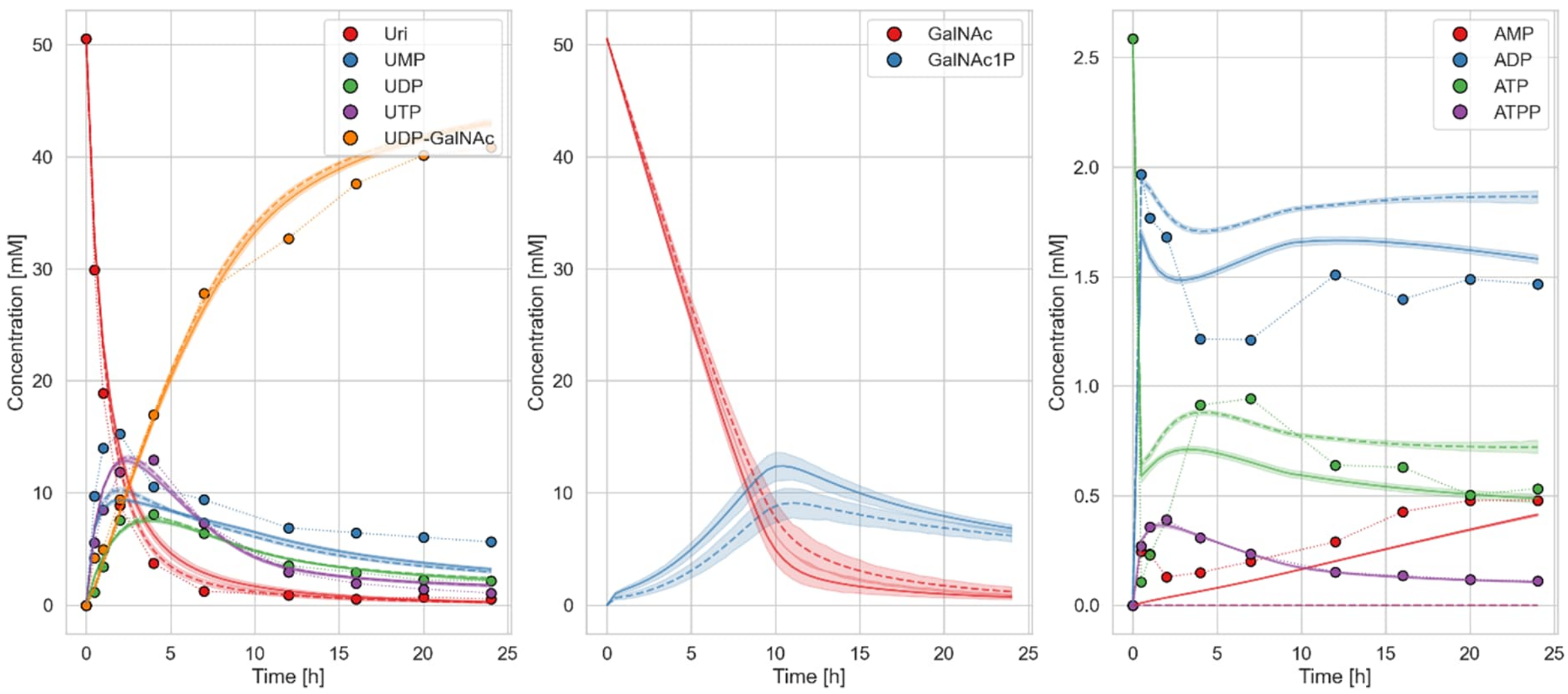

Comparison of model variants with data from experiment 11

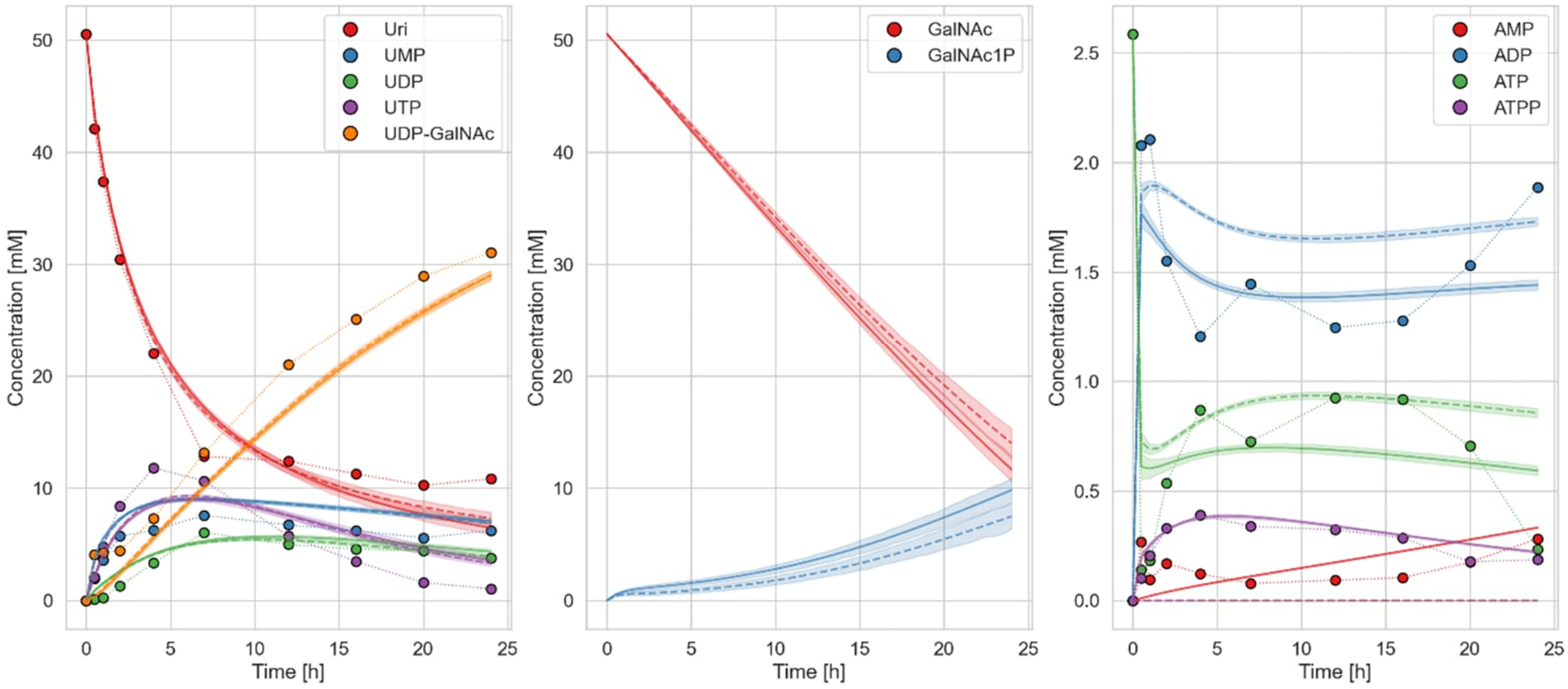

Comparison of model variants with data from experiment 12

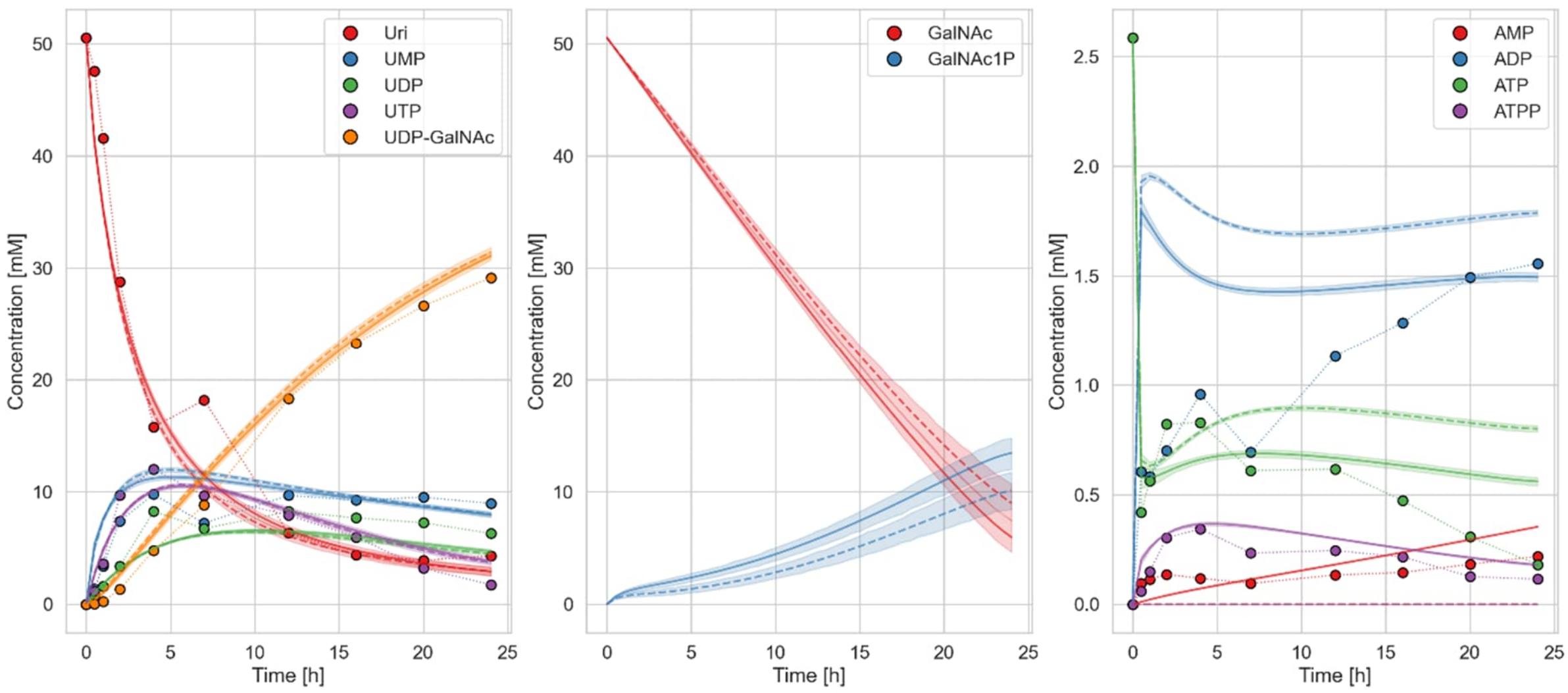

Supplement: Supplementary file 2 [file mmc2.pdf]
