# Supplementary material for: Combining automated kinetic model selection with robust optimization and application to maximize cell-free production of UDP-GalNAc
Source: Metab Eng Commun. 2026 Sep 8;23:e00287. doi: 10.1016/j.mec.2026.e00287 (PMC13634411; doi:10.1016/j.mec.2026.e00287)
Supplement: Supplementary file 3 [file mmc3.pdf]

Supplementary File S3

Combining Automated Kinetic Model Selection with Robust Optimization and Application to Maximize Cell-free Production of UDP-GalNAc

Nicolas Huber[1,#], Tuan Son Hoang[1,2, #], Sebastián Espinel Ríos[3], Thomas F.T. Rexer[1,4], Udo Reichl[1,2], Steffen Klamt[1, *]

[1] Max Planck Institute for Dynamics of Complex Technical Systems, 39106 Magdeburg, Germany

[2] Otto von Guericke University Magdeburg, 39106 Magdeburg, Germany

[3] School of Chemical and Bioprocess Engineering, University College Dublin, D04 V1W8, Dublin, Ireland

[4] eversyn, 39106 Magdeburg, Germany

*Corresponding author: klamt@mpi-magdeburg.mpg.de

[#]Equal contribution

Supplementary File S3: Results of process optimizations

<u>Distributions of optimal initial enzyme and substrate concentrations from the process optimization for the different parameter ensembles</u>

Box and scatter plots visualize the distributions of the identified optimal initial concentrations for each enzyme and substrate from all optimization calculations (repeated for each parameter set of the ensemble; 50 parameter sets). Red horizontal lines indicate the corresponding initial concentrations of the baseline experiment (UDP-GalNAc Exp.36, 50 mM). Boxes show the quartiles of the distributions, yellow horizontal lines indicate the medians, and whiskers cover the rest of the distribution except outliers (determined by a function of the inter-quartile range).

Optimization of the UDP-GalNAc titer

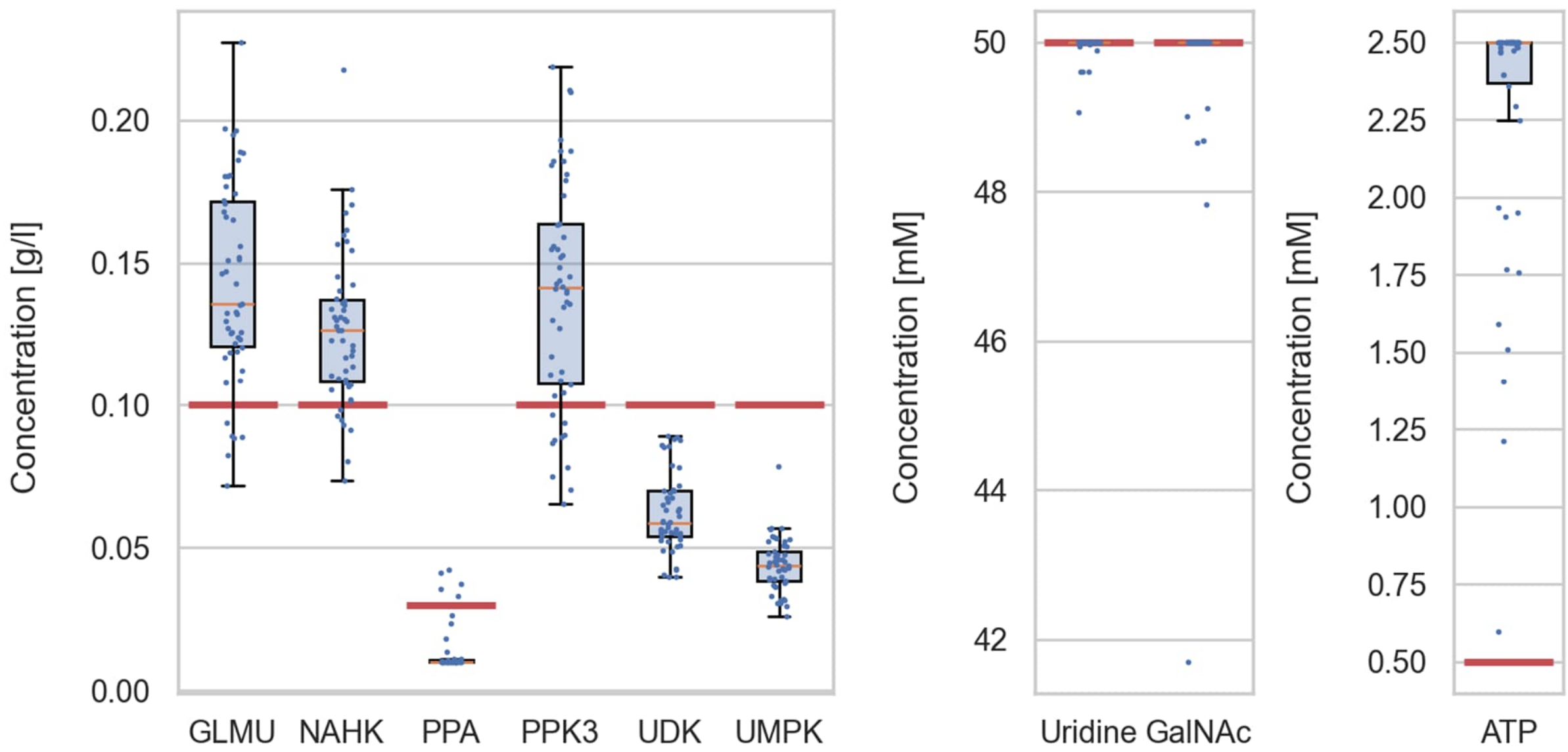

Optimization of the enzyme load

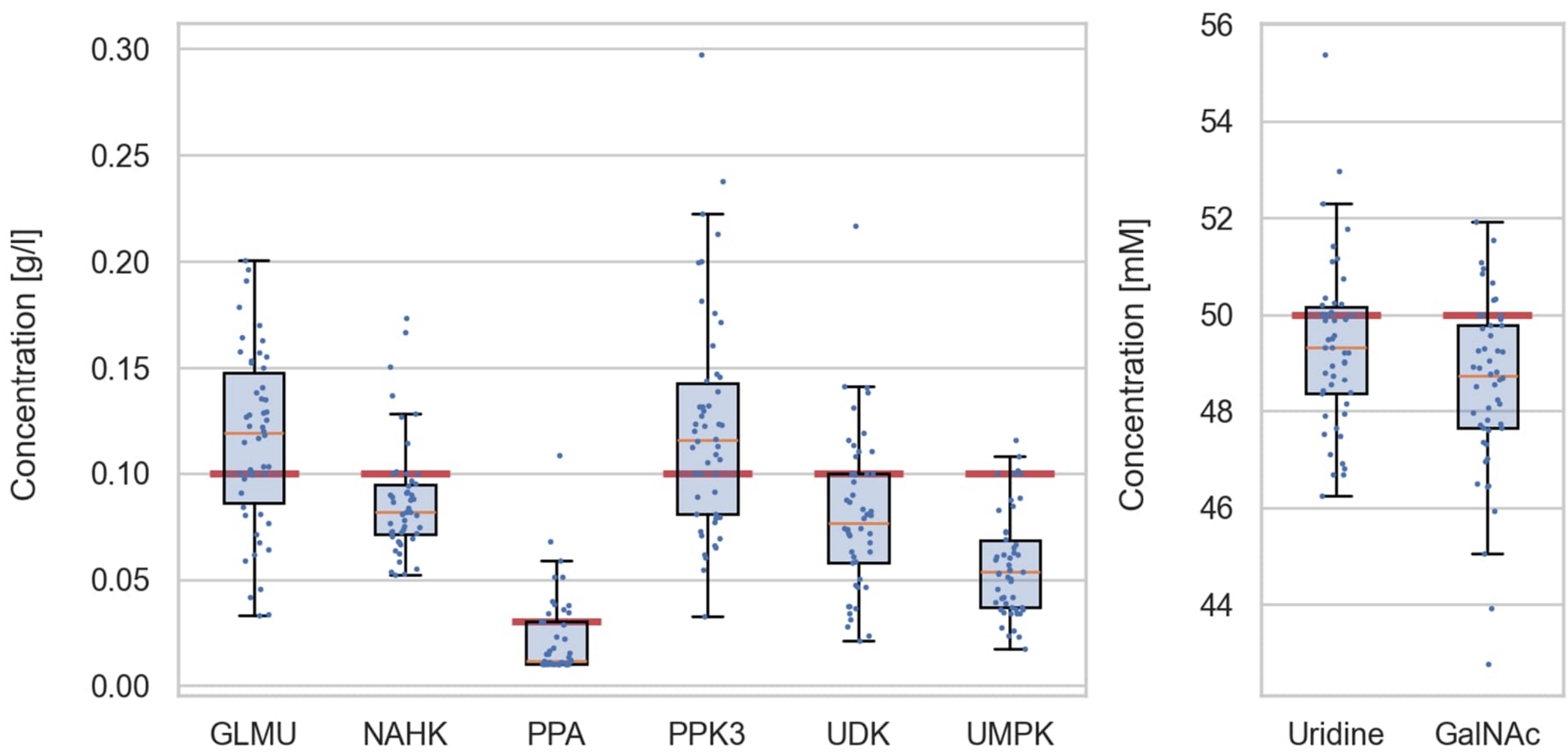

Cross-validation and scoring of the optimization results

Optimization of the UDP-GalNAc titer

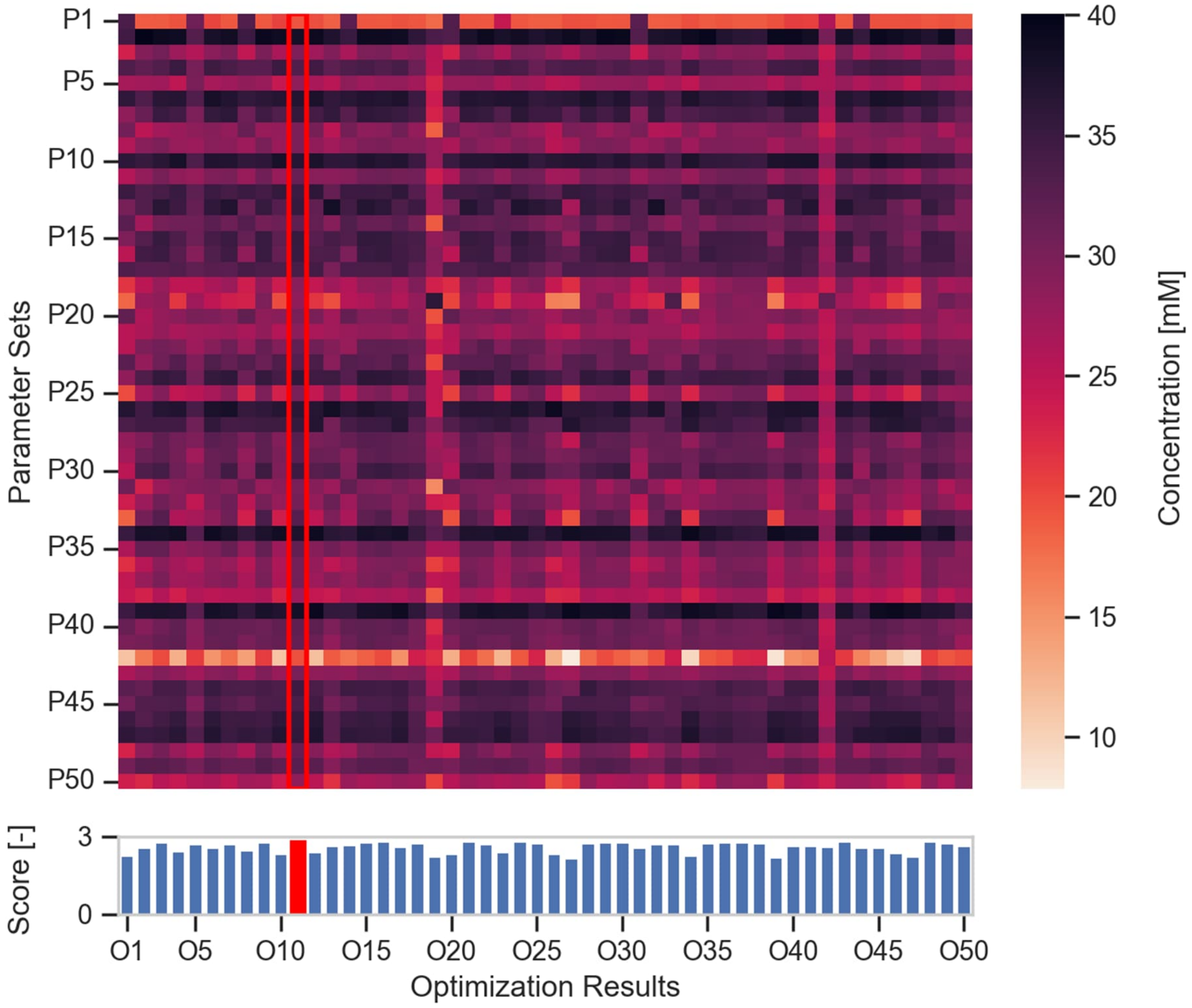

Cross-validation and scoring of all optimization results (O1 to O50 of the titer optimization) for all parameter sets of the parameter ensemble (P1 to P50). The cells of the heatmap are colored based on the UDP-GalNAc titer (at 7 h) that is reached by simulating the model with a certain combination of optimal initial concentrations (taken from a given optimization result; column index) and kinetic parameter values (taken from a given parameter set; row index). The diagonal of the square heatmap corresponds to all cases where the parameter set was used for the optimization while the off-diagonal elements were generated from additional simulations where the model was set to a new combination of optimization result and parameter set. For example, the array element P20/O30 refers to the predicted titer calculated by the model with its parameters set to those of P20 and its initial concentrations set to the optimization result O30. The bar chart below the heat map shows the score of each column (i.e., of each optimization result). The highest scoring optimization result is highlighted. The equation to calculate the score was: $score = \frac{col\_median}{best\_median} + \frac{col\_min}{best\_min} + \frac{col\_sum}{best\_sum}$ *where the numerators of each term refer to a statistical measure of a selected column while the denominators refer to the overall best value for this measure across all columns (highest median, highest minimum, highest sum). In this way, the value range of each term Is* [0,1] *resulting in an overall range for the score of* [0,3].

Optimization of the enzyme load

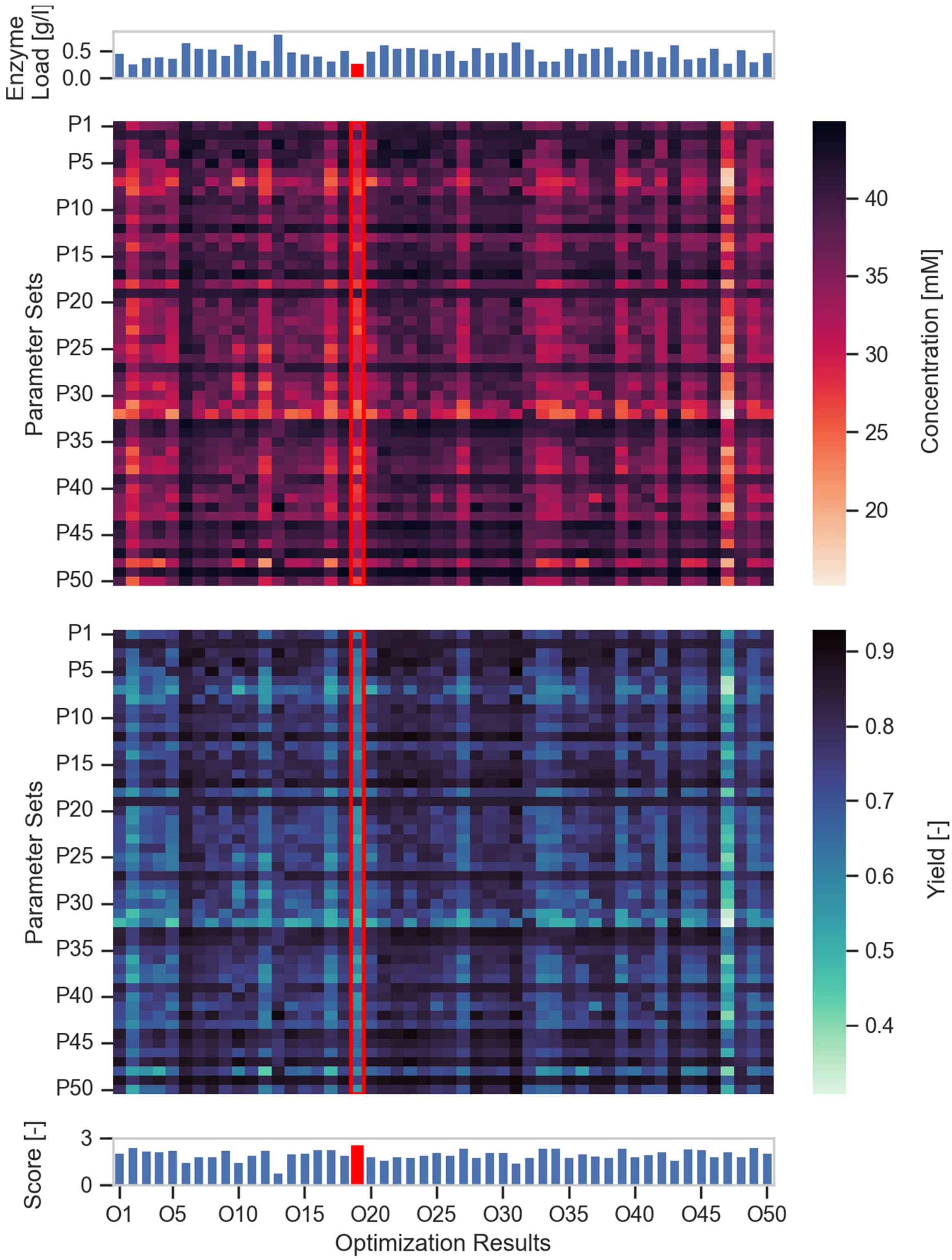

Cross-validation and scoring of all optimization results (O1 to O50) of the enzyme load optimization for all parameter sets of the parameter ensemble (P1 to P50). The cells of the heatmaps are colored based on (1) the UDP-GalNAc titer at the end of the process (upper heat map) and (2) based on the UDP-GalNAc yield (lower heat map) that are reached by simulating the model with a certain combination of optimal initial concentrations (taken from a given optimization result; column index) and kinetic parameter values (taken from a given parameter set; row index). The diagonals of the square heat maps correspond to all cases where the parameter set was used for the optimization while

the off-diagonal elements were generated from additional simulations where the model was set to a new combination of optimization result and parameter set. The upper bar chart shows the enzyme load of each optimization result. The bar chart below the heat maps shows the score of each optimization result. The highest scoring optimization result is highlighted. The equation to calculate the score was: $score = \frac{E\_tot\_col - 0.53}{E\_tot\_best - 0.53} + \min\left(1, \frac{titers\_col\_median}{40.37}\right) + \min\left(1, \frac{yields\_col\_median}{0.8274}\right)$*. The numerator of the first term is the enzyme load of a selected optimization result and the denominator is the overall best (lowest) enzyme load. Both values are compared to the enzyme load of the baseline (0.53). The second and third term are both capped at 1 and contain fractions that compare the median of the titer or yield values of a selected column to the corresponding titer or yield vale of the baseline experiment (which were included as constraints in the optimization problem). In this way, the value range of each term Is* [0,1] *resulting in an overall range for the score of* [0,3].
