# Supplementary material for: Combining automated kinetic model selection with robust optimization and application to maximize cell-free production of UDP-GalNAc
Source: Metab Eng Commun. 2026 Sep 8;23:e00287. doi: 10.1016/j.mec.2026.e00287 (PMC13634411; doi:10.1016/j.mec.2026.e00287)
Supplement: Supplementary file 4 [file mmc4.pdf]

Supplementary File S4

Combining Automated Kinetic Model Selection with Robust Optimization and Application to Maximize Cell-free Production of UDP-GalNAc

Nicolas Huber[1,#], Tuan Son Hoang[1,2, #], Sebastián Espinel Ríos[3], Thomas F.T. Rexer[1,4], Udo Reichl[1,2], Steffen Klamt[1, *]

[1] Max Planck Institute for Dynamics of Complex Technical Systems, 39106 Magdeburg, Germany

[2] Otto von Guericke University Magdeburg, 39106 Magdeburg, Germany

[3] School of Chemical and Bioprocess Engineering, University College Dublin, D04 V1W8, Dublin, Ireland

[4] eversyn, 39106 Magdeburg, Germany

*Corresponding author: klamt@mpi-magdeburg.mpg.de

[#]Equal contribution

Supplementary File S4: SDS-PAGE of the purified enzymes

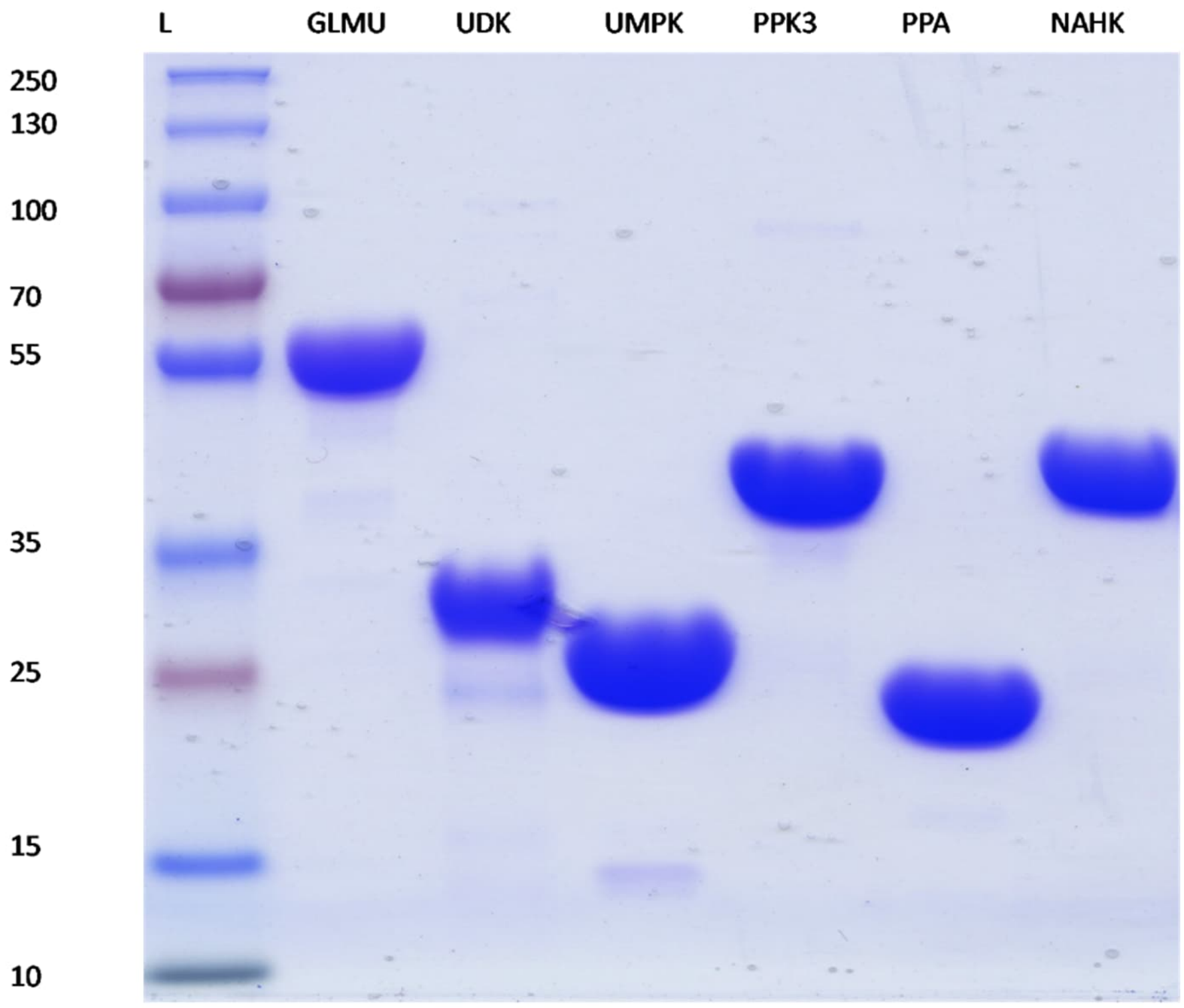

Figure A: SDS-PAGE of purified enzymes used for synthesis of UDP-GalNAc. About 2 µg of protein was loaded per well. Theoretical mass of enzymes (from left to right): GLMU, 49 kDa; UDK, 24 kDa; UMPK, 22 kDa; PPK3, 35 kDa; PPA, 19 kDa and NAHK, 40 kDa.
